# An Unsupervised Machine Learning Approach for the Automatic Construction of Local Chemical Descriptors

**DOI:** 10.1021/acs.jcim.3c01906

**Published:** 2024-03-18

**Authors:** Miguel Gallegos, Bienfait Kabuyaya Isamura, Paul L. A. Popelier, Ángel Martín Pendás

**Affiliations:** † Department of Analytical and Physical Chemistry, University of Oviedo, Oviedo E-33006, Spain; ‡ Department of Chemistry, The University of Manchester, Oxford Road, Manchester M13 9PL, U.K.

## Abstract

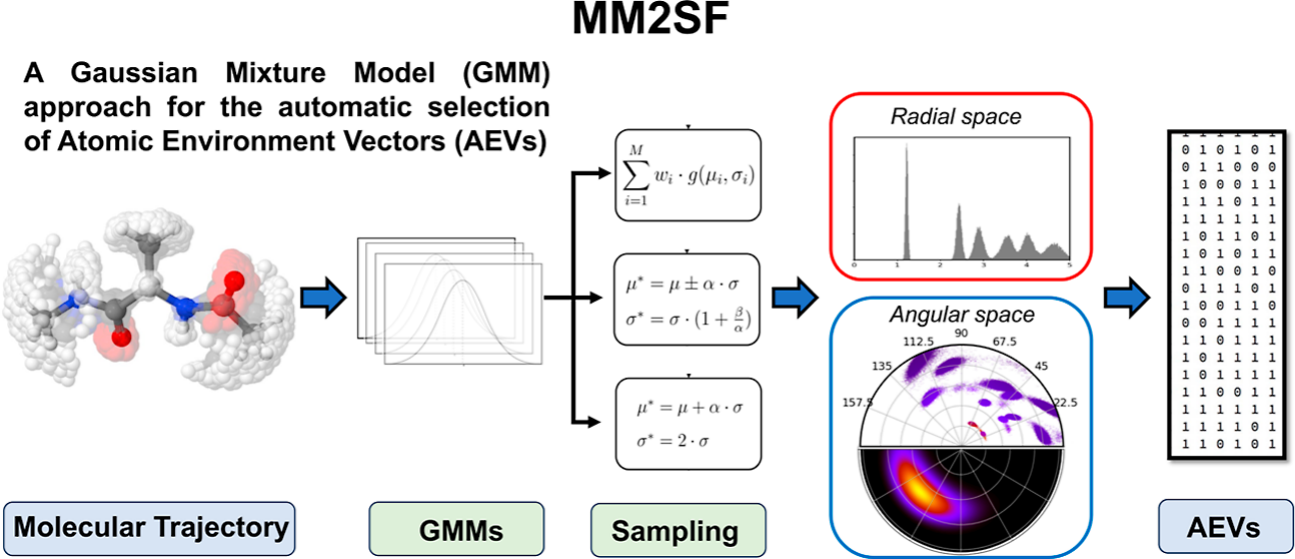

Condensing the many physical variables defining a chemical system into a fixed-size array poses a significant challenge in the development of chemical Machine Learning (ML). Atom Centered Symmetry Functions (ACSFs) offer an intuitive featurization approach by means of a tedious and labor-intensive selection of tunable parameters. In this work, we implement an unsupervised ML strategy relying on a Gaussian Mixture Model (GMM) to automatically optimize the ACSF parameters. GMMs effortlessly decompose the vastness of the chemical and conformational spaces into well-defined radial and angular clusters, which are then used to build tailor-made ACSFs. The unsupervised exploration of the space has demonstrated general applicability across a diverse range of systems, spanning from various unimolecular landscapes to heterogeneous databases. The impact of the sampling technique and temperature on space exploration is also addressed, highlighting the particularly advantageous role of high-temperature Molecular Dynamics (MD) simulations. The reliability of the resulting features is assessed through the estimation of the atomic charges of a prototypical capped amino acid and a heterogeneous collection of CHON molecules. The automatically constructed ACSFs serve as high-quality descriptors, consistently yielding typical prediction errors below 0.010 electrons bound for the reported atomic charges. Altering the spatial distribution of the functions with respect to the cluster highlights the critical role of symmetry rupture in achieving significantly improved features. More specifically, using two separate functions to describe the lower and upper tails of the cluster results in the best performing models with errors as low as 0.006 electrons. Finally, the effectiveness of finely tuned features was checked across different architectures, unveiling the superior performance of Gaussian Process (GP) models over Feed Forward Neural Networks (FFNNs), particularly in low-data regimes, with nearly a 2-fold increase in prediction quality. Altogether, this approach paves the way toward an easier construction of local chemical descriptors, while providing valuable insights into how radial and angular spaces should be mapped. Finally, this work opens the possibility of encoding many-body information beyond angular terms into upcoming ML features.

## Introduction

Research and development of numerous scientific disciplines, including Chemistry,^1,2^ have recently undergone a major paradigm shift, motivated by the emerging application of Artificial Intelligence (AI) tools.^3^ In the particular case of chemical sciences, different AI fields, such as Machine Learning (ML) or Deep Learning (DL), now play an absolutely pivotal role owing to their ability to efficiently solve complex problems in feasible time scales. The latter advantage has become especially notorious within computational chemistry,^4−8^ which commonly requires the execution of a vast number of resource-consuming tasks, often lacking explicit analytical solutions. Indeed, the synergy between AI and computational methods has crystallized into a wide range of contributions to the field including drug discovery,^9^ quantum chemistry,^10^ property prediction,^11^ or material sciences,^12^ to name just a few. Despite the success of the application of ML/DL approaches in the computational chemistry realm, its implementation is far from trivial. In fact, the application of AI in general faces multiple challenges. High-quality and voluminous data sets, often expensive to compute or obtain, are required to allow the ML models to distill meaningful underlying patterns within the reference data. Creating robust AI architectures meeting the accuracy and efficiency requirements for practical use demands a labor-intensive design and coding process. This is especially pronounced in their application to chemistry, where precision is of paramount importance, since many phenomena are driven by the delicate balance among numerous individual quantities, each with small values. Beyond that, the resultant accurate models tend to behave as black boxes, deprived of any interpretability and explainability, which is, moreover, coupled to the absence of reliable uncertainty metrics in the case of neural networks.

In this regard, building adequate input features that hold valuable information to predict the target output property under study, commonly termed the chemical featurization problem, still remains one of the major hurdles. On the one hand, condensing a molecular structure down to a finite set of features inevitably comes at the expense of some loss in the structural information that is captured. Indeed, the complexity of the chemical space, comprising countless compounds, makes it impossible to account for every chemical feature, often resulting in field-specific descriptors. Moreover, certain properties, such as stereoisomerism, cannot be naively described with the use of plain geometric features, adding an additional level of complexity. On the other hand, increasing the quality of the descriptor generally increases the cost involved in their computation, which may restrict their scalability and wide applicability. While a graph-like molecular model, with atoms and bonds corresponding to nodes and edges, respectively, provides a unique and recognizable representation in the chemist’s mind, the latter must be transformed into a machine-compatible description. Experimentally or computationally derived properties (e.g., p*K*_a_, partition coefficients or atomic charges) of the systems can sometimes be directly used for certain applications. However, theoretically grounded chemical descriptors are often the alternative of choice. The latter are commonly classified in terms of the extent of the dimensionality with which the chemical information on the system is recovered: 0D descriptors do not make explicit use of any molecular structure information, while their 1D analogs (relying on the chemical composition of the system) only capture certain functional groups or patterns of interest, which are usually stored in the form of a binary array. On the other hand, 2D representations emerge from the bidimensional molecular graph, usually in the form of pairwise features between neighboring atoms, such as the well-known Wiener branching indices.^13^ Going beyond simple molecular graphs paves the way toward capturing structural information on the molecules in the space resulting in 3D descriptors, which are at the same time classified in electronic, volume, and shape-based features. The simplest of these, also being the common starting point of more complex descriptors, are the plain *XYZ* Cartesian coordinates, which are widely employed in cheminformatics. Finally, grid-based features account for an additional physical property of the system as a fourth dimension, which can be relevant to predicting the target output property. We note in passing that, generally speaking, high-dimensional features (i.e., 3D or 4D) will increase the quality of the resultant descriptor at the expense of a higher computational cost. A thorough review of some of the most extended chemical descriptors along with their performances and applications is available to the reader in the literature.^14−17^

Among all of the aforementioned approaches, 3D-based descriptors offer a good compromise between computational cost and accuracy while relying on the exact physical variables determining a molecular system: composition, *Z*, and atomic positions, *R*. Additionally, structural features have been proven quite versatile, as evidenced by their successful application in many relevant fields such as QSAR,,^18^ quantum chemistry,^19^ and molecular docking,^20^ among others. In fact, these and similar features were already widely employed in computational chemistry way before the outburst of modern AI, with the circular Morgan features^21^ or the so-called atom pairs,^22^ relying on a discretized collection of atom paths between non-H atoms, dating back to 1965 and 1985, respectively. Since then, a great deal of effort has been put into developing more efficient ways to capture the chemistry of a system. Ideally, the resultant descriptor must provide a one-to-one mapping between the chemical and representation spaces, yielding a unique fingerprint for each molecule while responding to changes in the structure, composition, and environment. Furthermore, the inherent symmetries of the physical variables characterizing the system must be present in the chemical fingerprints, which should obey the corresponding permutational, rotational, and translational invariances. Finally, the featurization array must be intensive to the size of the system, while having moderate lengths to prevent the curse of dimensionality, which can have detrimental effects on the AI models. Therefore, it is not surprising that much research has been carried out in recent years in the quest for robust structure-based fingerprints, which has crystallized in a wide-variety of descriptors such as the 3D-MoRSE,^23^ EVA,^24^ WHIM,^25^ Coulomb matrices,^19^ ALF,^26^ ACSF,^27^ SOAP,^28^ BOB,^29^ or, more recently, SchNet.^30,31^

Within the latter, ACSF (Atom Centered Symmetry Functions), while relying on the Gaussian decomposition of the radial and angular environments, is one of the simplest and most intuitive ways to encode the local chemical environments of a given reference atom. Indeed, a plethora of ACSF flavors has emerged throughout the years to exploit their full potential,^32−35^ which has crystallized in countless ML applications.^36−40^ However, unlike end-to-end approaches such as SchNet,^30,31^ hand-crafted descriptors require careful fine-tuning of their underlying hyperparameters. Although some attempts have been proposed to aid this feature selection procedure,^34,41^ the work is still scarce and most of the approaches require a large evaluation of uniformly distributed symmetry functions. Besides requiring some prior knowledge of the system, which is not always obvious, the latter is a particularly tedious task that can hinder the development of ACSF-based models. Even worse, the suboptimal selection of these features can severely affect the potential performance of the predictive kernel. Therefore, a notable gap exists in the field, as very accurate descriptions can be obtained only after delicate and expensive optimization of the underlying kernel parameters.

To ameliorate such inconvenience, we introduce a novel approach for the automatic selection of the tunable ACSF descriptor parameters, relying on the exploration of the molecular energy surface. An unsupervised ML strategy, based on a Gaussian Mixture Model (GMM) clustering of the vibrational and conformational landscapes, is employed to isolate the most relevant domains of the sampled space. The latter are then used to construct chemical features tailor-made to adequately describe the relevant radial and angular domains that allow a clear-cut distinction between the local chemical environments found across the database. This workflow, implemented in a Python code, is put to the test with the prediction of the Quantum Theory of Atoms in Molecules (QTAIM)^42^ atomic charges of a prototypical model of biological relevance: peptide-capped alanine. Our results demonstrate the validity and wide applicability of this approach, which efficiently explores the radial and angular spaces to extract highly specific chemical features. Not only does our approach effectively encode the environment with high quality but also it simplifies the process, opening the door to incorporating higher-order features beyond angular terms whose complexity has hindered their application in common ML descriptors. The manuscript is organized as follows: first, a brief overview of the theoretical background behind our approach, including ACSF, GMM, and QTAIM, is provided. Then we delve into the basic workflow of the code and its algorithmic details, followed by a thorough analysis of the performance of the optimized radial and angular ACSF in the prediction accuracy of the models, tested under different ML architectures. The impacts of the sampling technique and temperature used to explore the space are also discussed, showcasing how our approach is generally applicable across widely different molecules. Also, a comparison of the performance and efficiency of our method, with respect to more conventional approaches is included using the NNAIMQ^37,43^ database as a testbed scenario. The final section contains the conclusions drawn from this work.

## Theoretical Background

### Atom Centered Symmetry Functions

Atom Centered Symmetry Functions (ACSFs), introduced by Behler in 2011,^27^ are one of the most widely employed descriptors in computational chemistry, owing to their simplicity, built-in invariance, easy-implementation, and readily available analytical gradients. In fact, ACSFs have been extensively used in the field for the construction of ML models targeted at the prediction of countless properties including the electronic energy,^44^ atomic forces,^39^ or partial charges,^37^ to name just a few. ACSFs employ a collection of Gaussian kernels to encode the local chemical environment of the individual constituents of a chemical system. Being a local descriptor, only those particles belonging to the immediate vicinity of a reference atom will contribute to the chemical fingerprint of the latter. Usually, two-body (2B) and three-body (3B)-dependent ACSF functions are used to capture the radial and angular neighborhoods, while higher order terms (e.g., dihedrals) are not explicitly included for the sake of simplicity.

**Figure 1:**
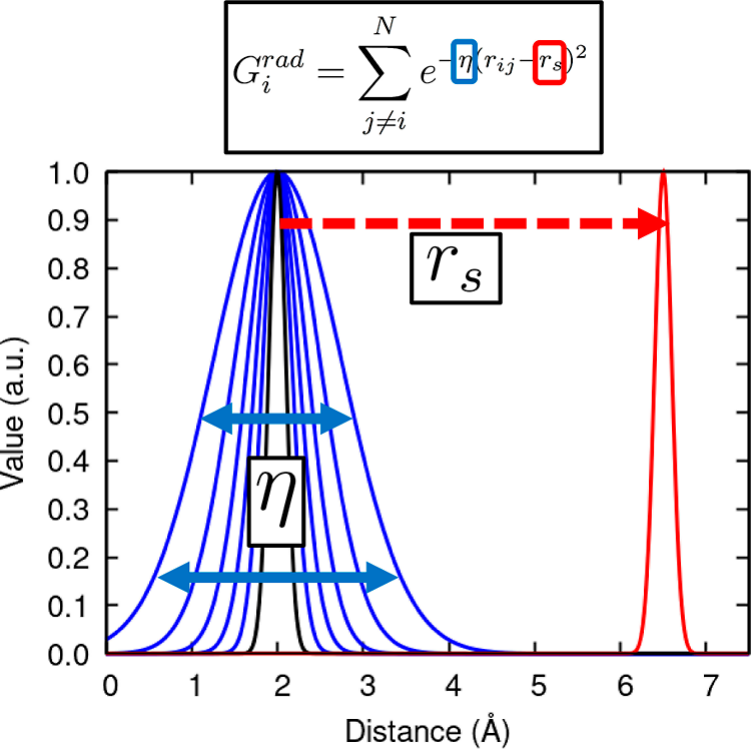
Effect of the *r*_s_ (red) and η (blue) parameters in the spatial distribution of a standard radial symmetry function. The following values were employed, *r*_s_ = {2.0, 6.5} Å and η = {0.90, 2.0, 4.0, 8.0, 17.3, 50.0} Å^–2^.

**Figure 2:**
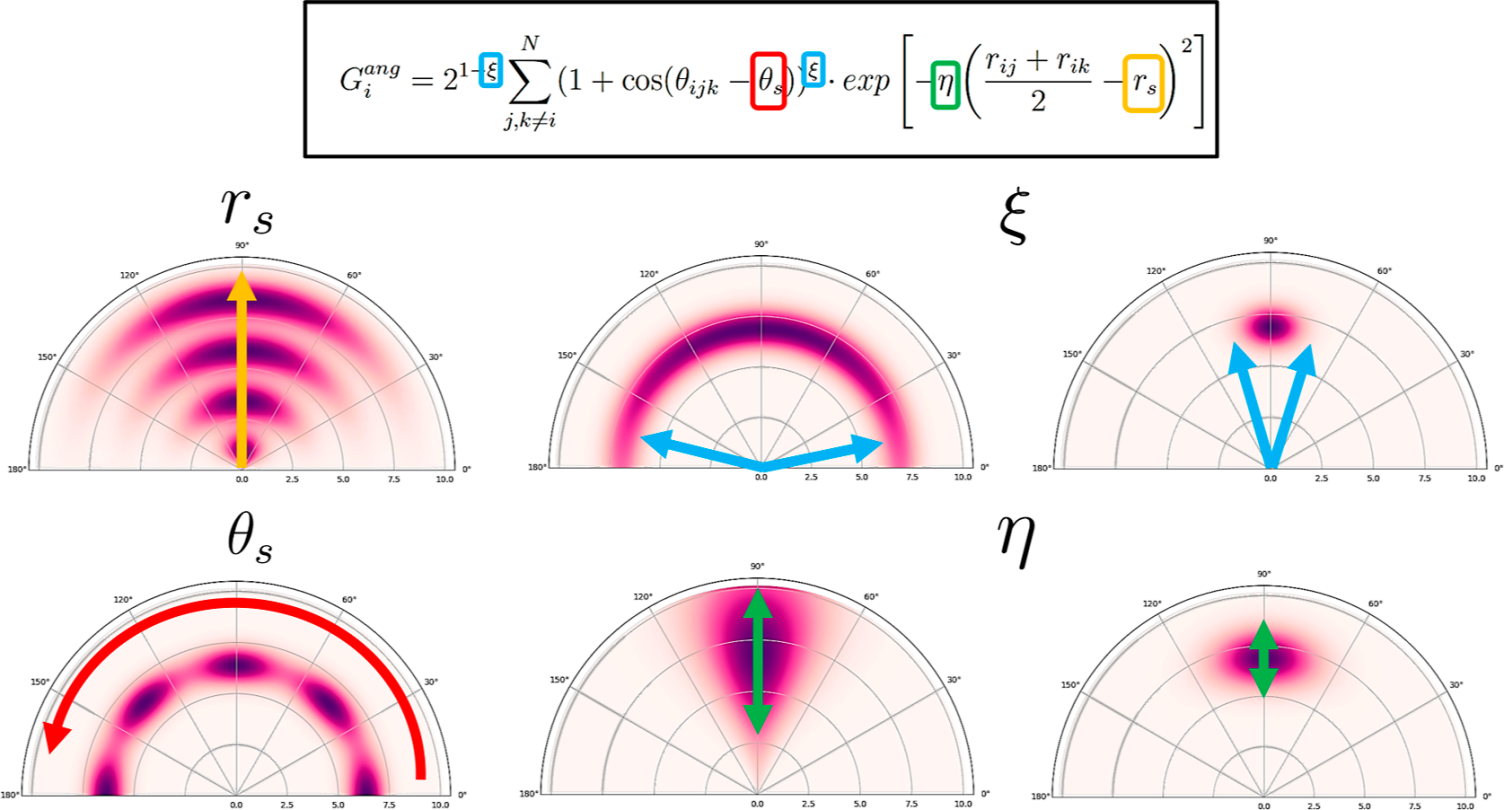
Effect of the *r*_s_ (orange), θ_s_ (red), ξ (blue), and η (green) parameters in the spatial distribution of a heavily modified angular symmetry function. The following values were employed *r*_s_ = {1.0, 3.5, 6.0, 8.5} Å, ξ = {1, 90}, θ_*ijk*_ = {0, 45, 90, 135, 180°}, and η = {0.05, 0.50} Å^–2^.

Equation 1 describes the functional form of a standard radial symmetry function evaluated at a reference atom *i* embedded within a N-atom system, given in terms of the pairwise distances *r*_*ij*_
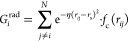
1where *r*_s_ and η define the center and width of the exponential function, respectively, and *f*_c_ represents the cutoff function used to impose the aforementioned locality. As exemplified in Figure 1, increasing the *r*_s_ values shifts the function further away from the reference atom, whereas larger η values reduce the radial domain covered by the latter. By the same token, the simplest angular function can be written as
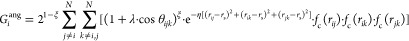
2where θ_*ijk*_ is the angle formed by reference particle, *i*, and the two neighboring atoms, *j* and *k*. As shown in Figure 2, parameters ξ and η define the width of the function in the angular and radial dimensions, respectively. As such, increasing these variables enhances the specificity of the angular ACSF function within a narrower range of angles and distances. Just as in the case of the radial ACSF functions, the *r*_s_ parameter controls the actual center of the angular function in the radial domain. In a similar way, λ, with values of ±1, shifts the center of the angular distribution at 0 or 180°, thereby imposing a symmetric behavior around the angular maxima. Alternative formulations to the latter have also been proposed to obtain flexible kernels. For instance, the modified angular symmetry function,^27^ removes the *jk* cutoff constrain in the radial counterpart so that medium-distance configurations can still be captured
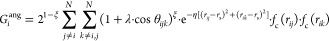
3

Analogously, the heavily modified angular ACSF,^44^ substitutes the previous λ parameter for a more versatile angular offset, θ_s_
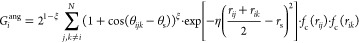
4which allows to shift the center of the function to any arbitrary position in the angular domain, something key to obtain a more accurate description of the system, as evidenced from the bottom left panel of Figure 2. We also note in passing that additional approaches, such as weighted ACSF,^32^ have also been proposed with an eye on reducing the number of specific terms required to describe a given environment.

From a practical point of view, a relatively large group of different ACSFs targeted at describing particular chemical features must be used to achieve a faithful reconstruction of the corresponding local atomic environments, collected in the so-called Atomic Environment Vectors (AEVs). Furthermore, the hyperparameters of each of the radial and angular terms constituting the latter must be finely tuned to adequately encode the molecular geometry of the system. However, such a task is far from trivial owing to the combinatorial growth of possible atomic groups with chemical diversity. Although prior knowledge of the system can guide the selection of the ACSF parameters, their fine-tuning often requires tedious and expensive manual optimization. In fact, such a dependence on a predefined set of radial and angular basis functions is often considered as one of the major limitations of ACSFs. Furthermore, ACSFs are relatively short-sighted, being unable to explicitly capture changes in the chemical environment beyond their cutoff radius.

### Gaussian Mixture Models

Generally speaking, a Mixture Model (MM) can be understood as a statistical approach (i.e., a probabilistic model) that describes an intricate data population as a multicomponent system arising from the combination of a finite number of simple distributions. In this way MMs, and in particular Gaussian MMs (GMM), for which the single-component functions are assumed to follow a normal distribution, are one of the most widely employed data clustering techniques.^45^ Indeed, and owing to their ability to expose the underlying patterns found in complex, unlabeled, sample populations coupled to a relatively easy implementation and use, GMMs have been found useful in countless applications within the field of unsupervised ML.^46−49^ One of the main strengths of GMM is its capacity to analyze intricate distributions due to the inherent flexibility of its components, which can take on various shapes and sizes. Besides decomposing distributions and identifying clusters, GMMs act as versatile model generators, capable of creating new data instances that align with the observed features of the reference clusters. These features make GMM particularly well-suited for exploring extensive and intricate data distributions, explaining its widespread use in ML and data analysis. GMMs also suffer from known limitations, such as the sensitivity of the clustering efficiency with the number of components. Indeed, determining the number of clusters giving rise to the observed distribution is a challenge lacking a unique solution. Moreover, as discussed later, GMMs depend on certain mathematical assumptions that may not hold true for all types of data distributions.

Under the GMM perspective, each of the points of the real distribution arises from the weighted combination of its underlying Gaussians, such that the actual probability distribution is the weighted sum of the latter, as shown in eq 5
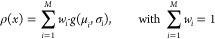
5where *w*_*i*_ represents the weight of the Gaussian g(μ_*i*_, σ_*i*_)
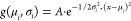
6to the whole mixture of *M* normal distributions. Each of the components of the GMM model is characterized by three basic parameters: μ (the center of the distribution), σ^2^ (the variance of the distribution), and *w* (its contribution to the whole sample pool). Having an equivalent functional form to that already discussed for the radial ACSF, shown in eq 1, the meaning of the parameters appearing in the exponential kernel is readily available: while μ displaces the centroid of the Gaussian,  determines its effective width in the space, as shown in Figure 1. Thus, performing a GMM clustering involves a basic numerical optimization task to find the means, variances, and weights of each of the *M* components to the total mixture, something that is achieved following the so-called expectation-maximization (EM) strategy:

- The μ and σ are (randomly) initialized for each of the *M* clusters in which the sample population will be divided. Similarly, equal weights are set initially for all the single-component normal distributions, .
- Expectation: for each of the points constituting the actual sample population (*x*_*i*_), the likelihood of being a member of any of the *M* clusters of the model is computed. These probabilities, *r*_*i*_^c^, can be estimated from the corresponding probability density functions of each component, which take the following form, for the simplest case of a unidimensional distribution

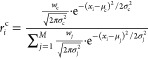
7

It is worth noting that such probabilities represent the relevance of a given Gaussian function to the existence of each of the data points, making GMM a soft clustering technique.

- Maximization: the μ, σ and *w* features of each GMM cluster (c) are updated according to the previously computed probabilities, as

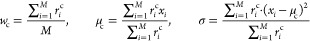
8

The updated parameters are used to rerun the expectation step, and this process is repeated iteratively until convergence is achieved in the GMM hyperparameters. From a practical point of view, different constraints can be imposed on the covariance matrix, leading to a wide variety of GMM flavors with different flexibilities in the shape and distribution of the individual components of the clusters. Furthermore, the final result may slightly vary depending on the starting conditions used to initialize the GMM runs due to the numerical nature of the optimization procedure.

As previously mentioned, a GMM will decompose a system in as many clusters as the components given to the model. Although prior knowledge of the system under study may help identify the number of constituents giving rise to the observed data, this task may be far from trivial in more intricate scenarios. To ameliorate this problem, automatic approaches for the determination of the optimum number of GMM components have been developed throughout the years. Usually, the clustering algorithm is run for a progressively increasing number of cluster (components) and a scoring metric is evaluated at each point to determine the optimum value, see Supporting Information Section S4.2 for further details.

### Quantum Theory of Atoms in Molecules

The Quantum Theory of Atoms in Molecules (QTAIM),^42^ employs an exhaustive partitioning of the real-space, dictated by the topology of the electron density ρ(**r**), to obtain a detailed and robust picture of a chemical system from a rigorous and minimalist perspective. Each of the regions (Ω), corresponding to the attraction basins of ρ(**r**), in which *R*^3^ is exhaustively decomposed, is well-defined, allowing the exact decomposition of global expectation values as a sum of their local (e.g., (intra)atomic or interatomic) contributions.

For instance, the number of electrons of a molecular system, *N*, can be written as the combination of local electron counts, *N*_A_
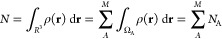
9from which the rigorous definition of partial QTAIM charges emerges naturally
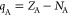
10where *Z*_A_ is the atomic number of the atom attributed to the atomic basin Ω_A_. Moreover, statistical analysis allows to further disguise the total electron count in its localized, λ_A_, and delocalized, δ_A,B_, counterparts
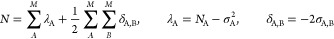
11being σ_A_^2^ and σ_A,B_ the variance and covariance of Ω_A_ and between Ω_A_ and Ω_B_, respectively. All of the aforementioned indices have proven to be very valuable metrics to study a wide variety of phenomena thus playing a crucial role in the rationalization of chemical intuition.^50−55^

## Algorithmic Details

The current section comprises a brief summary of the computational workflow involved in the self-optimization of radial and angular symmetry functions. Further details about the code and its underlying scheme can be found in Section S4 of the Supporting Information.

**Figure 3:**
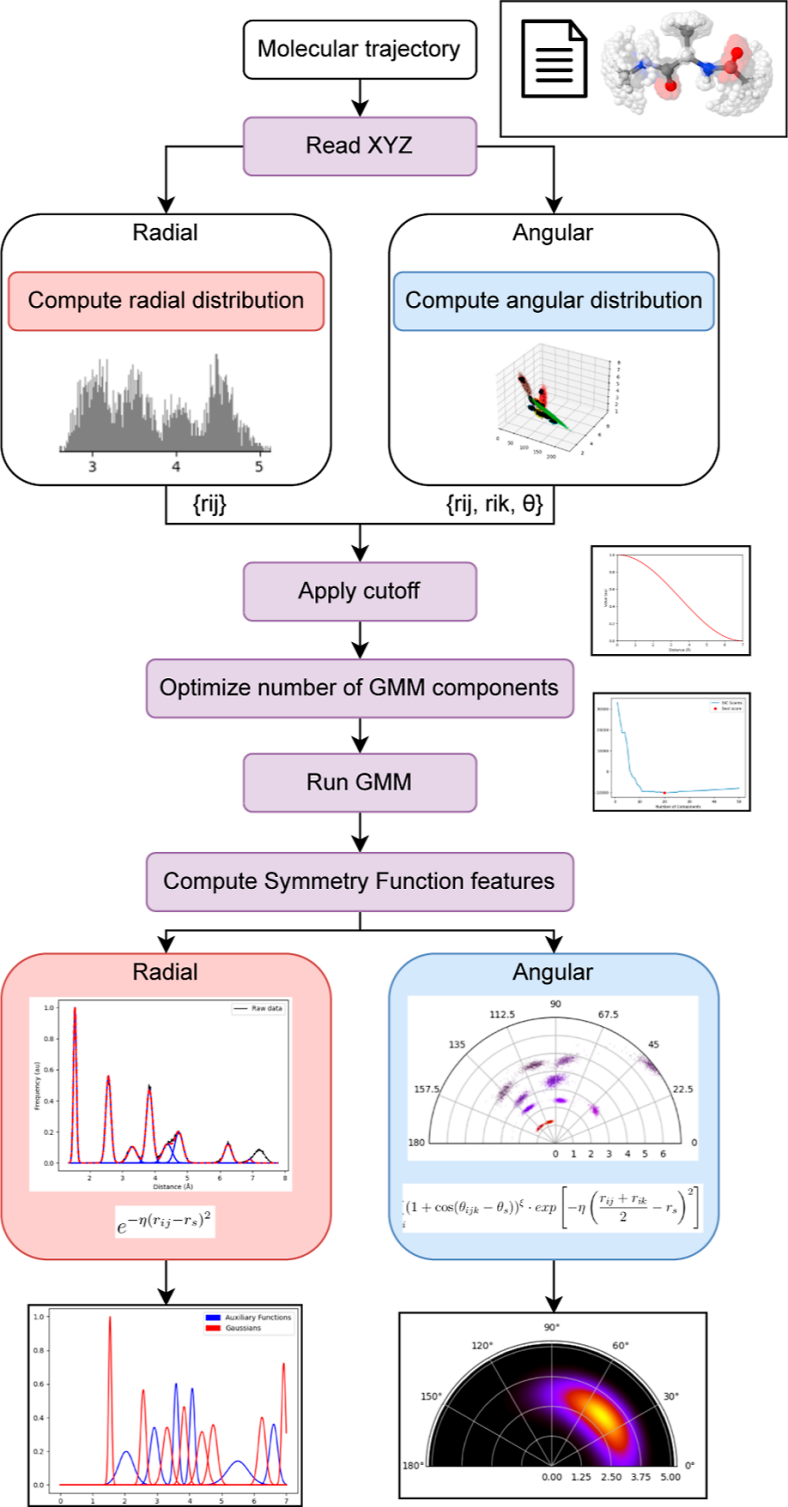
Schematic architecture of the code, showing the main procedure involved in the optimization of the radial and angular symmetry functions. The common functions are shown in violet, whereas the radial/angular-specific are highlighted in red and blue, respectively. The labels *r* and θ are used to refer to the pairwise distances and angles, respectively.

Figure 3 shows a schematic representation of the architecture of the code. The fine-tuning of the symmetry function hyperparameters requires access to a moderately large collection of molecular snapshots. In order to obtain a robust and reliable picture of the local chemical environments of the systems under study, these environments should arise from a representative and unbiased sampling of the corresponding energy landscape, as such that provided by Normal Mode Sampling (NMS) or Molecular Dynamics (MD) simulations. Additional information on the NMS technique is provided to the reader in Section S8 of the Supporting Information. In the case of MD, medium to high temperatures should be used to ensure a thorough scanning of the potential energy surface (PES), something that can be monitored with the aid of the mist plots (see Supporting Information’s Section S2 for further details). From a practical standpoint, representative samples are those that offer a comprehensive assessment of the molecular energy landscape. Consequently, they should account for the various conformers that may arise from exploring the rotational and vibrational degrees of freedom whenever feasible. Unfortunately, achieving this goal can be far from straightforward, especially for chemically diverse and large systems, as the intricacy and dimensionality of the PES tend to explode with molecular size. In these challenging scenarios, running a collection of simulations starting from different seed structures is notoriously advantageous, especially for the NMS approach, which is inevitably confined to exploring the immediate vicinity of the starting potential well. Likewise, and as mentioned above, high temperature simulations are often desirable, as they will generally result in better behaved ACSF features, capable of covering a wider range of the radial and angular domains. Ultimately, the choice of the sampling strategy depends on the system’s complexity and the specific target application. For instance, if the resulting features are intended primarily for describing the equilibrium domains of a molecule, then a less thorough sampling (shorter and lower temperature simulations) may suffice.

These molecular frames, collected in a standard molecular trajectory file, are read by the code from which the *XYZ* coordinates are retrieved. The variable trj_step controls the sampling frequency, which allows subsample of the whole molecular trajectory to save computational time. The radial, {*r*_*ij*_}, and angular, {*r*_*ij*_, *r*_*ik*_, and θ_*ijk*_}, distributions are then computed for all of the frames. Only the upper-triangular tensor is used in the particular case of the latter to account for the potential redundancies arising from the three equivalent descriptions of a given atomic trio, owing to the origin dependence of the inner angle (further information about this is available in Supporting Information’s Section S4.4.1). Since symmetry functions are local chemical descriptors, the radial and angular data can be truncated up to a given cutoff radius (*r*_c_). Currently, the code implements two different cutoff approaches: a soft scheme, relying on the application of cosine kernel as shown in eq 12, and a hard (truncation) scheme, which discards all the data beyond *r*_c_
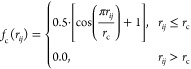
12where *r*_*ij*_ and *r*_c_ represent the pairwise distances and the cutoff radius, respectively.

As previously mentioned, the GMM model requires the prior specification of the number of components giving rise to the observed data distribution. Being a task far from trivial, the best-performing number of clusters is optimized with an internal routine. Since multidimensional data distributions are prone to model overfitting, different score metrics and optimization criteria (e.g., Bayesian Information Criterion, BIC) have been implemented for such a purpose (see Section S4.2 in theSupporting Information). Using the previously optimized number of clusters, the radial or angular data distributions are fitted to a GMM model, from which the {*w*, μ, and σ} parameters of each of the clusters are obtained. For the sake of simplicity, the *M* GMM components are sorted by decreasing weights such that the top fraction, gathering the most relevant contributions to the observed chemical features of the system, can be selected. We note in passing that this is particularly relevant when dealing with very sparse data distributions, which can often result in an impractically large number of GMM normal distributions and, in turn, too intricate AEVs.

The parameters of the radial and angular ACSF functions are finally obtained by mapping the GMM features, {*w*, μ, and σ}, to the corresponding functional forms as dictated by eqs 1 and 4, respectively. In the particular case of the two-body terms, the radial ACSF parameters *r*_s_ and η can be straightforwardly obtained from the mean (μ) and standard deviation (σ) of each of the GMM components
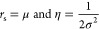
13

Likewise, the width and the center of the angular ACSF kernel in the radial space, respectively denoted η and *r*_s_, are obtained following a similar proxy accounting (however, see eq 4 for the use of a single radial grid for the *r*_*ij*_ and *r*_*ik*_ pairwise distance dimensions)
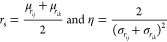
14where {μ_*r*_*ij*__,μ_*r*_*ik*__} and {σ_*r*_*ij*__, σ_*r*_*ik*__} are the mean and standard deviation of the three-dimensional GMM in the *r*_*ij*_ and *r*_*ik*_ domains. Unfortunately, finding the remaining parameters for the angular dimension of the three-body GMM cluster is considerably more challenging, as the exponential and cosine kernels of the GMM and heavily modified ACSF lack an exact analytical correspondence. As such, the angular width (given by the ξ parameter) is found through a simple numerical (iterative) optimization routine while imposing a constraint in the angular shift, θ_s_ = μ_θ_, being μ_θ_ the centroid of the GMM cluster in the angular distribution. Further details on the procedures employed for the radial and angular ACSF feature mapping are provided in Supporting Information, Sections S4.3 and S4.4. After doing so, a collection of optimized radial and angular ACSF functions is obtained for each atom type and neighboring atomic or interatomic environments (as shown in the bottom panels of Figure 3). Incidentally, in certain scenarios the distributions will show a prominent discretization, which generally results in unevenly distributed ACSF functions. Although the latter observation emerges from the intrinsic chemistry of the system, it may be convenient to include additional functions to avoid gaps in the description of the local chemical environment. For such a purpose, we introduce the possibility of accounting for additional ACSF, referred to as auxiliary functions, built to ensure a minimum (not null) overlap between contiguous distributions (see Supporting Information’s Section S4.3).

**Figure 4:**
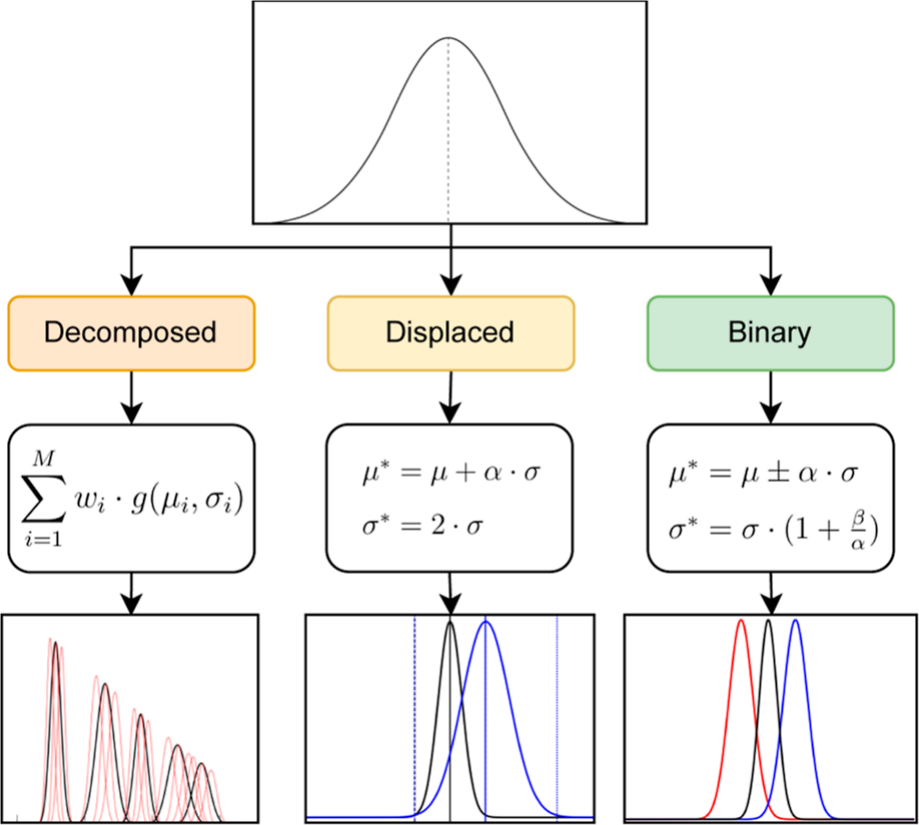
Alternative sampling schemes for the optimization of the Gaussian components in the radial space: decomposed Gaussians (left), displaced Gaussians (center), and binary Gaussians (right).

Altogether, the latter collection of functions, which we call tailor-made, is distributed in the space to match the exact shape of the observed chemical environment distributions. As discussed later throughout the manuscript, modifying the spatial arrangement of these functions may increase the resolution of the resultant description. In this context, and trying to optimize the accuracy of the AEVs, we have implemented three different resampling schemes, as summarized in Figure 4. In the decomposed approach, each cluster is expressed as a collection of *K* additional Gaussian functions, called subcomponents, which will increase the resolution with which that region of the potential energy landscape is described. On the other hand, the two remaining strategies try to break the symmetry of the functions with respect to the centroid of the cluster. This guarantees an unambiguous description of the lower and upper bounds of the cluster (e.g., the bond compression and lengthening regimes in the particular case of the radial symmetry functions). In the displaced Gaussians, this is achieved by introducing an offset so that the whole cluster [μ – α·σ, μ + α·σ] is uniquely covered by the lower tail of the symmetry function. In contrast, binary Gaussians use a pair of functions to describe the lower and upper bounds of each cluster so as to ameliorate the uneven resolution achieved with the previous approach. Note that the slope of the displaced Gaussian is not uniform throughout the cluster resulting in an uneven sensitivity of the symmetry functions to a perturbation of its chemical environment along the space covered by the cluster. As forthcoming discussions will elucidate, distributing the functions according to the latter scheme is proven beneficial, affording particularly high-quality features. As expected, these approaches boost the descriptor resolution at the expense of increasing its dimensionality. Thus, special care should be taken to prevent the deterioration of the generalization abilities of the models. Additional details on the different sampling schemes are provided in Section S4.5 of the Supporting Information.

## Computational Details

The training and evaluation of the Feed Forward Neural Network models (FFNN), used to check the performance of the descriptors optimized here, were performed within a standard CONDA environment, running under a Python 3.8 interpreter on an Intel(R) Xeon(R) Silver 4114 CPU at 2.20 GHz computer equipped with 40 cores distributed over 2 nodes and 128 GB RAM. Unless otherwise specified, all tests were run in parallel, using 20 distributed cores. All FFNN models were trained in an element-wise way; that is, a single ML model was built for each element type in the data set. Besides FFNNs, atom-wise Gaussian Process Regression (GPR) models, or GPs for short, were also built to check and assess the performance of our features in drastically different architectures. All GP models were trained by FEREBUS,^56,57^ an in-house GPR engine written in modern Fortran, accelerated via OpenMP and significantly improved very recently.^58,59^ These models were trained and tested in parallel using 20 cores on compute nodes with Cascade Lake Xeon Gold 6230 CPUs of 2.10 GHz clock speed each. Additional details on the architecture and training parameters of the GP and FFNN models are available in Sections S3 and S7 of Supporting Information. Generally speaking, the performance of the ML models presented here will be reported in terms of the Mean Absolute Error (MAE) and Root Mean Squared Error (RMSE) metrics, which have also been used to monitor the quality of the models during their training. Further details on these performance metrics can be found in Supporting Information, Section S3. Unless otherwise specified through the text, the self-tuned ACSF features introduced here were used to construct the input vector fed to the ML models. For the sake of convenience, all the features were normalized with respect to the training subset; see Supporting Information Section S3 for more details.

The structure of peptide-capped alanine was optimized in the gas phase without dispersion or relativistic corrections at the B3LYP/6-311G level of theory with the aid of the Gaussian09^60^ quantum chemistry package. The latter was also employed to obtain the corresponding wave functions along with the eigenvalues of the Hessian matrix used to characterize the nature of the stationary points found. In order to thoroughly sample the Potential Energy Surface (PES), Molecular Dynamics (MD) simulations were performed at 500 K starting from the previously optimized geometry using AMBER22.^61^ A time step of 1.0 fs was employed, and the temperature was controlled with the aid of a Langevin thermostat with a 0.7 coupling factor. A total of 20,000 individual frames, uniformly sampled, was extracted from the MD trajectory. For each frame, single point calculations were performed at the B3LYP/6-31+G(d,p) level of theory in the gas phase to obtain the corresponding wave functions. The same methodology was used to ensure the quality of the reference data that will be used to evaluate the performance of the descriptors. On the other hand, the QTAIM analysis of each of the latter snapshots was performed with the AIMAll^62^ code.

Since the quality of the reference data can have a noticeable impact on the actual performance of the models, the previous data were filtered on the basis of energetic and electronic criteria resulting in roughly 19,000 valid molecular instances. Further details on the construction of the database employed here can be found in Section S1 of Supporting Information.

Unless otherwise specified, all the QTAIM metrics will be reported in electrons (e) throughout this manuscript. All molecular representations appearing in the figures were rendered with the Jmol suite.^63^

## Results and Discussion

**Figure 5:**
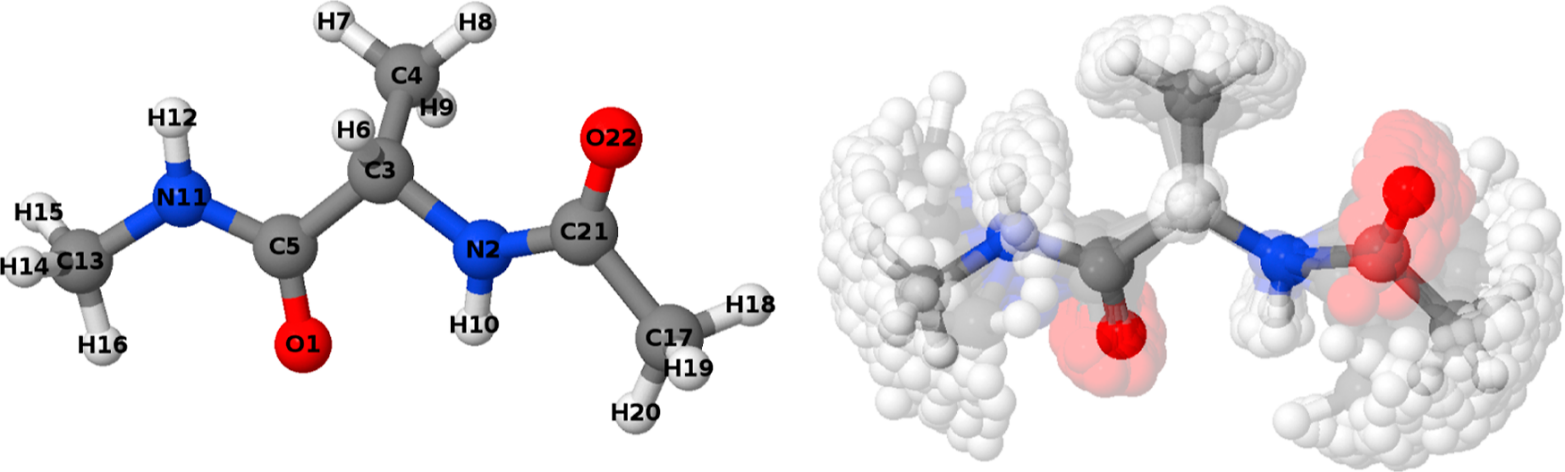
Optimized geometry of capped alanine at the B3LYP/6-311G level of theory in the gas phase (left) along with a representation of the sampled conformational space throughout the simulation (right). Molecular representations have been rendered with Jmol.^63^

Figure 5 shows a representation of the chemical environments sampled throughout the MD simulations of (peptide-)capped alanine; additional representations are provided in Section S2 of the Supporting Information for the sake of clarity. As can be seen, fairly decent sampling is achieved for the main functional groups in the system, something that is particularly pronounced for the more flexible moieties in the skeleton such as CH_3_ or OH. These trends become even more evident from the analysis of the mean displacements of the atomic positions and their corresponding deviations displayed in Table 1. In contrast, the peptidic bonds impose the well-known geometrical constraints limiting the sampling around these domains. We note that this is not a flaw of the simulation but a result of the restricted motion of the system. Although different techniques could be used to further sample the more rigid regions of the PES, real systems at moderate temperatures are unlikely to visit these nonfluxional areas.

**Table 1:** Mean (μ) and Standard Deviation (σ) of the Atomic Displacements of Capped Alanine throughout the MD Trajectory^a^

atom	μ	σ	Frg	atom	μ	σ	Frg
1O	0.44	0.36	C=O	12H	0.55	0.43	NH
2N	0.19	0.11	NH	13C	0.43	0.25	CH_3_–NH
3C	0.00	0.00	ref	14H	1.52	0.70	CH_3_–NH
4C	0.22	0.12	CH_3_–CH	15H	1.31	0.60	CH_3_–NH
5C	0.18	0.11	C=O	16H	1.41	0.66	CH_3_–NH
6H	0.18	0.09	C–H	17C	0.42	0.24	CH_3_–NH
7H	1.27	0.69	CH_3_–CH	18H	1.34	0.60	CH_3_–NH
8H	1.28	0.70	CH_3_–CH	19H	1.50	0.68	CH_3_–NH
9H	1.28	0.68	CH_3_–CH	20H	1.36	0.63	CH_3_–NH
10H	0.36	0.21	NH	21C	0.32	0.18	CO
11N	0.29	0.22	NH	22O	0.54	0.35	CO

a All values are reported relative to the starting geometry and in Å. The Frg label indicates the most relevant functional group or chemical moiety to which each atom belongs to.

**Figure 6:**
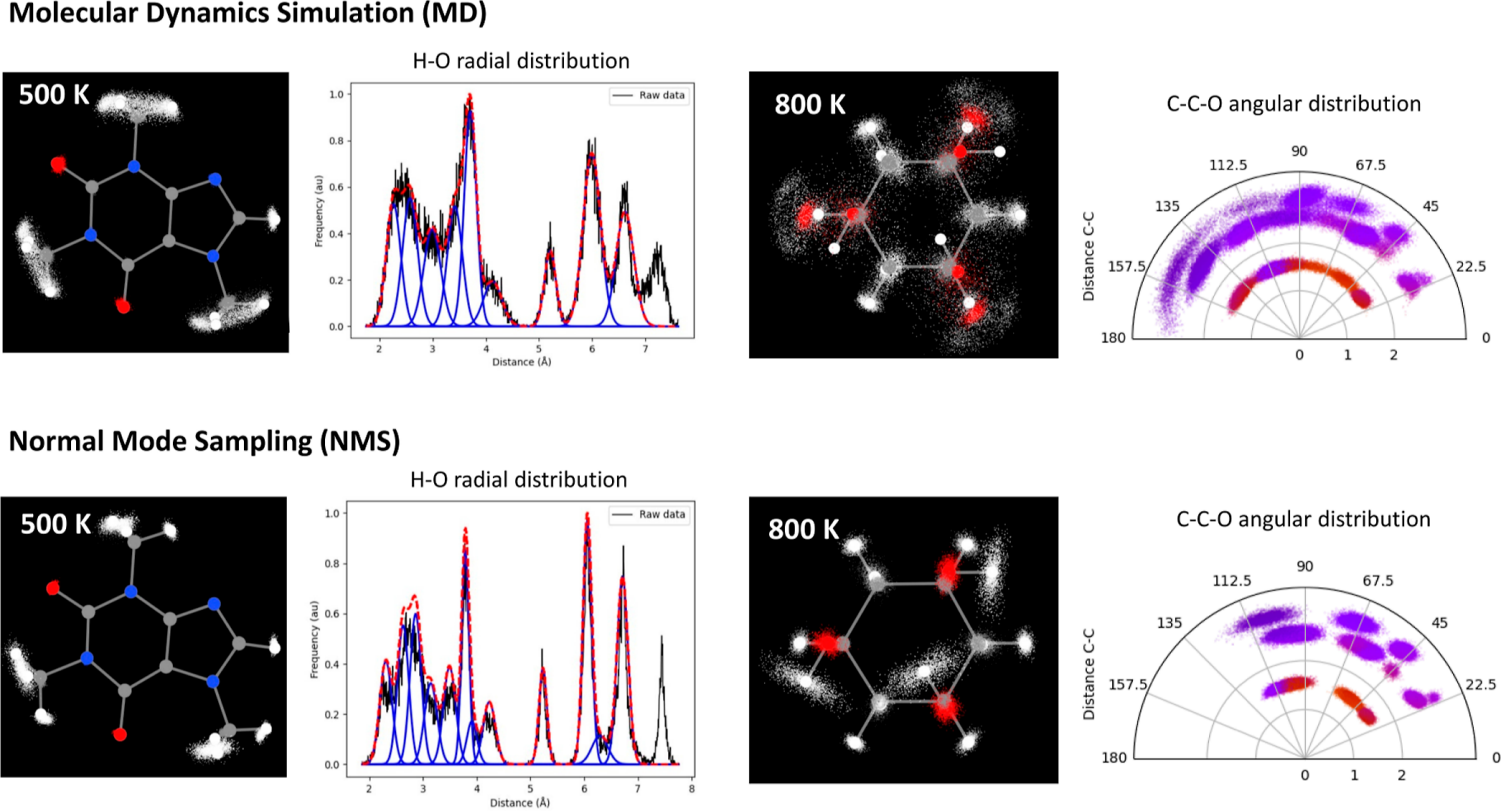
O–H radial and C–O–O angular distributions of the caffeine and 1,3,5-cyclohexanetriol molecules, respectively, as sampled by the NMS and MD techniques. The temperature used in each case is also reported in kelvin. Further details on the sampling procedure can be found in the Supporting Information, Section S8.

Furthermore, we also studied the impact of the sampling technique on the extent with which the angular and radial spaces are explored. Figure 6 compares the angular and radial spaces covered by the NMS and MD samplings of 1,3,5-cyclohexanetriol and caffeine, selected from a larger collection of testbed systems (Supporting Information Section S8). Our findings reveal that despite both techniques exploring similar regions of conformational space, MD appears to provide a more extensive sampling, particularly around rotatable bonds and flexible scaffolds. Consequently, MD tends to offer wider radial and angular clusters spanning more space, as evidenced by Figure 6. This discrepancy, which is less pronounced for a more rigid molecular backbone (e.g., caffeine), arises from the NMS limitation to oscillate around the initial equilibrium position, hindering its ability to capture degenerate or quasi-degenerate wells in the potential energy surface.

Increasing the temperature leads to larger atomic displacements for both techniques, leading to a more thorough exploration of the energy landscape while widening the gap between the NMS and MD explorations of the space. With enough thermal energy, MD simulations can explore additional degrees of freedom, being able to overcome certain energetic barriers, and thus inducing major conformational changes. This is clearly reflected in the results found for 1,3,5-cyclohexanetriol, which undergoes a significant structural shift between the two chair conformations at 800 K. This effect is clear from the mist plots collected in the top panel of Figure 6 and is exclusively captured by MD simulations. In contrast, the NMS trajectory continues to sample the immediate vicinity of the starting species, as has been outlined before. However, it should be noted that in the particular case of intermolecular systems, high temperature MD can overcome the energy barrier present and result in a partially unbounded system which provides a not very relevant sampling of the space. This is clearly evident in the case of the water dimer (see Supporting Information Section S8), where running the MD trajectory at 500 K results in two barely non bonded isolated water monomers. Conversely, the NMS technique, constrained by a harmonic potential around the well, proves valuable for roughly exploring the equilibrium PES, even for weakly bound intermolecular systems. In this way, the exploration of intermolecular systems presents a distinct challenge due to the inherent complexities associated with adequately navigating their conformational spaces.

All things considered, and although the selection of the sampling technique ultimately depends on the specific application and system under consideration, the use of high temperature MD is deemed particularly beneficial owing to its ability to properly explore various PES regions beyond the starting equilibrium. Finally, it is worth mentioning that the unsupervised clustering technique presented here is able to efficiently decompose the radial and angular domains of very diverse molecular scaffolds regardless of their chemical composition, connectivity, or rigidity, beyond the capped alanine used as a prototypical example in this work. More importantly, the success of our approach in the meticulous decomposition of local chemical environments suggests the potential to extend it beyond angular domains. Such expansion may encompass many-body effects beyond three-body terms, which could lead to even more precise chemical fingerprints and potentially better-performing models.

We will now discuss the performance of the optimized symmetry functions in the prediction accuracy of the atomic charges of capped alanine. For the sake of simplicity, the models will at first be solely fed with radial information. Once the ideal conditions for the construction of the symmetry functions have been found, three-body information will be included to obtain a reliable picture of the real-case performance of the descriptors presented here. We will generally report the training error metrics instead of the most conventional testing performance as we aim to explore the quality of the self-optimized features rather than producing models for specific applications. While never-seen data sets provide a more rigorous assessments of a model’s generalization abilities, training metrics offer clearer insights into the limit in performance of a model. Such an intentional choice allows for a more distilled and unbiased examination of how different factors influence the features under optimal conditions, offering valuable insights for our specific objectives. Furthermore, as will be shown in the upcoming discussions, nearly identical training and testing performances are found, with common discrepancies of ≤0.001 electrons.

### Tailor-Made vs Evenly Distributed Radial ACSF

The selector of radial symmetry functions was run on the whole 20,000 sample pool in combination with a (hard) cutoff radius of 7.00 Å. The minimum BIC metric was employed to find the optimum number of clusters (searching for up to 15 different components). Given the large number of samples, GMM clustering was applied on the raw data without any smoothing.

**Figure 7:**
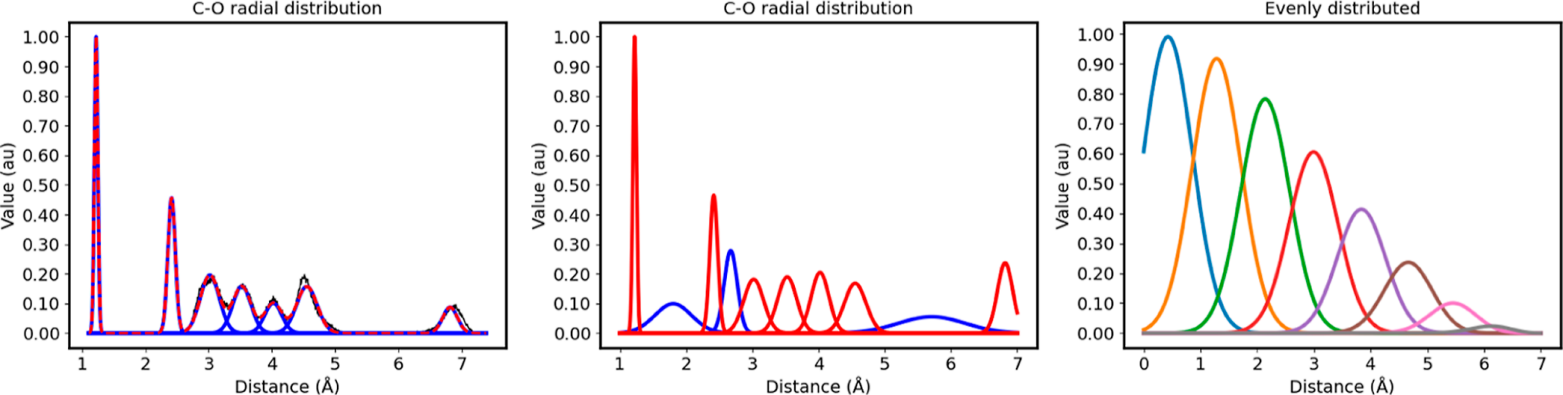
(Left) Observed C–O radial distribution of capped alanine along with the individual clusters found by the GMM model. (Center) Tailor-made radial symmetry functions for the O local environments of C atoms, *G*_rad_^C,O^, of capped alanine. The auxiliary functions, used to ensure non-null overlaps between neighboring distributions, are shown in blue. (Right) Evenly distributed radial functions made to match the same number of components as those of their tailor-made analogs. The *y*-axis shows the relative frequency of the observations in arbitrary units.

The left panel in Figure 7 shows the decomposition of the radial environments of the C–O atom pairs in the database (raw data shown in black) in terms of their optimized GMM components (each of them is shown in blue, whereas the sum of all of the Gaussians is shown in red). The GMM model is able to perfectly reconstruct all the radial environments resulting in the clear-cut clustering of the data in easily recognizable chemical moieties (e.g., C=O ketone).

**Figure 8:**
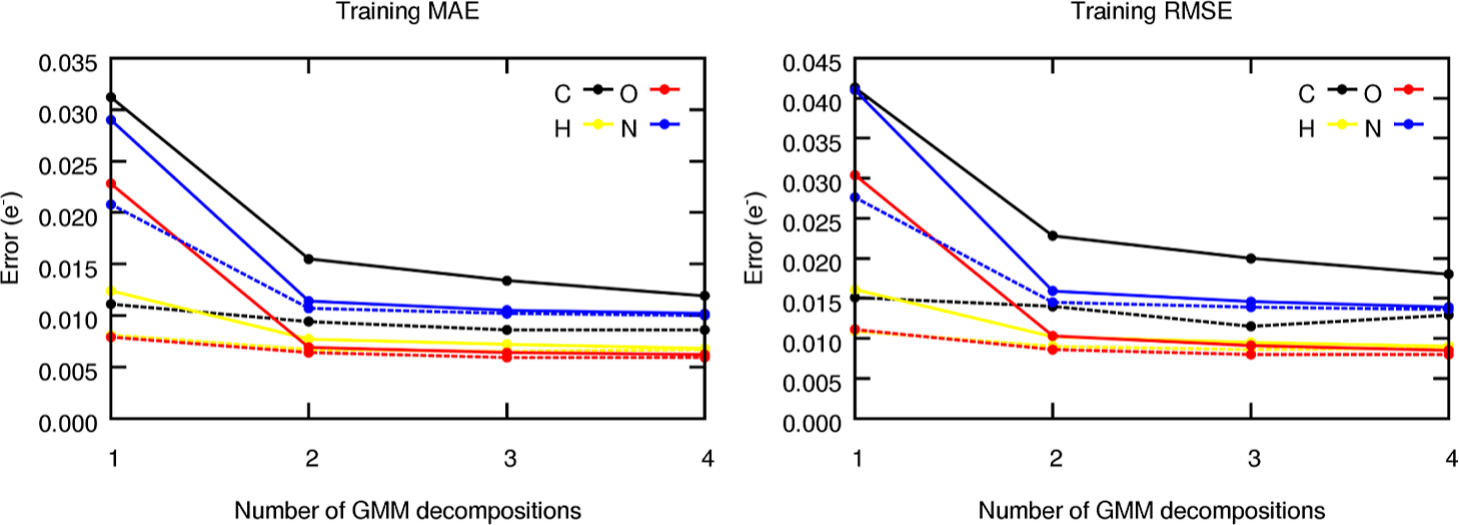
Evolution of the training MAE and RMSE metrics, in electrons, of the FFNN models trained to predict the atomic charges of capped alanine as a function of the number of decompositions of each GMM component. Dashed lines are used to show the effect of including auxiliary functions. The results for the testing subset are gathered in Figure S16.

We now explore the raw performance of the tailor-made functions along with the effect of their decomposition and inclusion of auxiliary components, as shown in Figure 8. For this purpose, radial ACSF were built from the previously found GMM components, and multiple AEV sets were obtained by decomposing each GMM component, up to a 4-fold splitting, as shown in Figure S9 of Section S4.5 in the Supporting Information. As expected, the error metrics decrease with the number of subdecompositions (*K*), something that is also observed for the testing data set (see Section S5.2 in the Supporting Information). This finding suggests that the latter approach increases the resolution of the AEVs in the description of the local environments. Generally speaking, the performance seems to converge quite quickly, showing the major increase in prediction accuracy for the 2-fold decomposition and saturating at around *K* ≈ 3–4. These observations arise as a result of the interplay between the increasing resolution and descriptor length. The penalizing effect of the latter disrupts the generalization abilities of the models, increasing the risk of overfitting for large *K* values. Additionally, an excessive decomposition can make the normal distributions to spread outside the boundaries of the cluster, where little to no valuable information can be extracted. This “overflow” effect would introduce dummy features in the input descriptor, rendering the training of the ML models difficult. Similar trends are also found in the evolution of the testing error metrics, collected in Section S5.2 of the Supporting Information. Additionally, with the particular exception of the C atoms, auxiliary functions result only in a very subtle increase in the model accuracy. In agreement with our intuition, the impact of the latter becomes less pronounced with the increasing values of *K*. This result points out that the benefit brought by auxiliary functions is likely to arise from the description of the cluster boundaries, which aids the discrimination of certain chemical environments.

To further delve into the quality of the self-optimized symmetry functions, their performance was compared to that provided by a uniformly distributed radial basis with the same number of components, as detailed in Section S4.3.1 of Supporting Information. The latter are fuzzy functions that, unlike the previous approach, lack any specificity in the description of particular functional groups or fragments. As shown in Table 2, evenly distributed radial bases outperform tailor-made symmetry functions even when the latter are decomposed. Indeed, the minimal even radial basis, corresponding to 29, 36, 23, and 20 features for C, H, O, and N, respectively, is generally able to surpass the prediction accuracies shown by heavily decomposed tailor-made functions (*K* = 4). However, we stress that evenly distributed functions suffer from severe drawbacks despite showing decent performance. Indeed, as the radial grid is decreased (resulting in more functions), the AEVs become more prone to contain dead functions, covering a region of the space that is unlikely to be sampled and thus resulting in null input features. Indeed, we had to account for such a problematic scenario by removing those functions that never became activated throughout the training data set.

**Table 2:** Training Error Metrics in the FFNN Prediction of the Atomic Charges of Capped Alanine as a Function of the Number of Radial ACSF Employed^a^

atom	N_feat_	MAE_tr_	RMSE_tr_	MAE_tr_	RMSE_tr_
C	29	0.031	0.041	0.008	0.011
	58	0.016	0.023	0.008	0.010
	87	0.013	0.020	0.008	0.012
	116	0.012	0.018	0.008	0.011
H	36	0.012	0.016	0.006	0.008
	72	0.008	0.010	0.006	0.008
	108	0.007	0.010	0.006	0.008
	144	0.007	0.009	0.006	0.008
O	24	0.023	0.030	0.006	0.008
	48	0.007	0.010	0.006	0.008
	72	0.006	0.009	0.006	0.008
	96	0.006	0.009	0.006	0.008
N	20	0.029	0.041	0.013	0.018
	40	0.011	0.016	0.009	0.012
	60	0.011	0.015	0.009	0.012
	80	0.010	0.014	0.009	0.012

a Left and right, respectively, show the results for tailor-made (without auxiliary functions) and evenly distributed Gaussians. For the latter, the same number of functions as that dictated by the former was used. All values are reported in electrons. The results for the testing subset are gathered in Table S3.

The previously discussed lower performance of tailor-made radial SF, albeit counterintuitive at first glance, arises from the symmetry introduced in the input features when the Gaussian kernels reproduce the exact radial distributions. In such a scenario, the ACSF features are unable to distinguish bond shortening or elongation phenomena, thereby resulting in inconsistent features. We note in passing that the use of the cutoff function perturbs the symmetry of the Gaussian and thus slightly alleviates the problem of imposing artificial symmetries in the system. As the number of GMM decompositions is increased, each cluster is described by a more flexible collection of functions and thus allowing a nonsymmetric collective description even in spite of its symmetric individual components. These findings would suggest that the increase in performance observed with the number of GMM decompositions in the tailor-made approach is likely to predominantly arise from the symmetry rupture of the features rather than from the explicit increase in radial resolution.

### Symmetry Breaking Effects in the Radial Resolution

**Figure 9:**
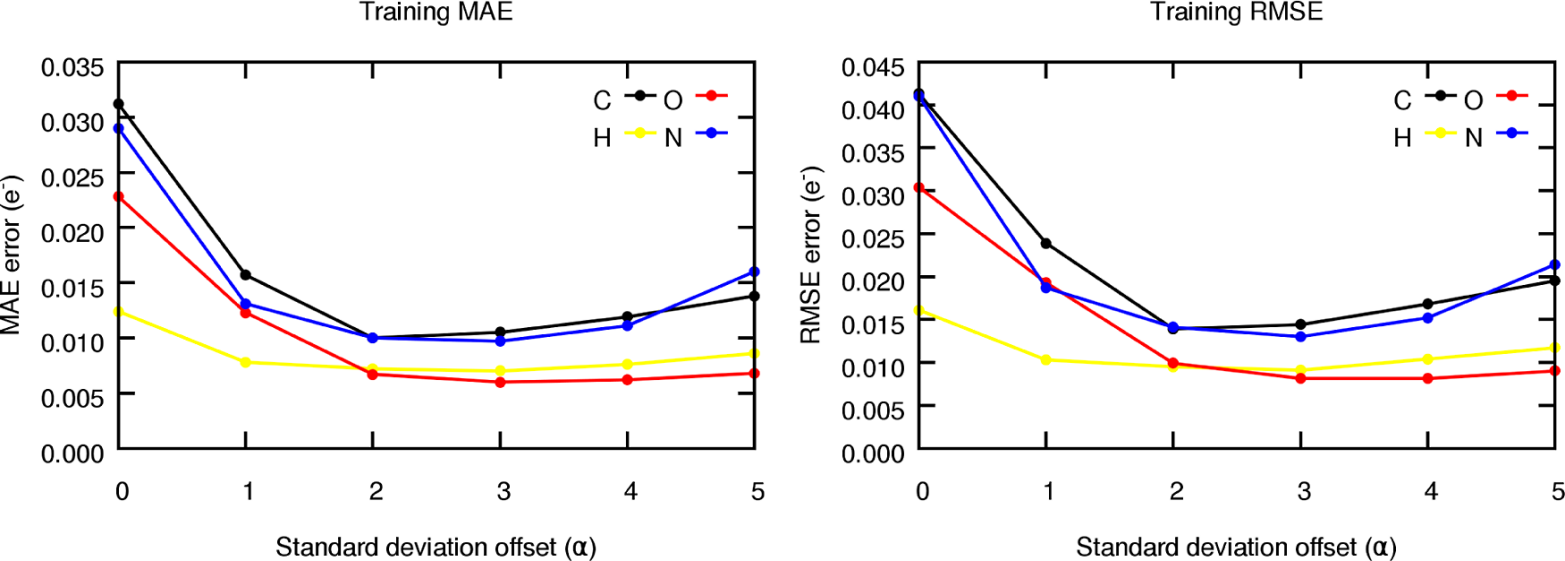
Evolution of the training MAE and RMSE metrics, in electrons, of the FFNN models trained to predict the atomic charges of capped alanine as a function of the offset (α) of the displaced Gaussians. The results for the testing subset are gathered in Figure S17.

In order to test the validity of the aforementioned hypothesis, the radial symmetry functions were redistributed in space according to the schemes explained in the Algorithmic details section. The latter were built from the previously optimized tailor-made functions and used to train the FFNN models in the same conditions as before. Figure 9 shows the evolution of the training error metrics, reported as MAE in electrons, as a function of the offset employed in the displaced Gaussians. The results for the testing data set are gathered in Section S5.4 of the Supporting Information.

As shown in Figure 9, the prediction accuracy shows clearly convex behavior, which is compatible with an intuitive and appealing explanation. Indeed, large σ parameters initially increase the range of values uniquely described by a Gaussian function, resulting in better prediction accuracy. In fact, the performance peaks at α = 3, corresponding to the sweet spot where the lower tail of the function spans along μ ± 3·σ, comprising 99.7% of the observed cluster population. It should be noted that such an observation is interesting on its own, as it demonstrates that breaking the symmetry of the functions offers a more adequate representation of each radial environment, in agreement with our previous hypothesis. Further increasing the offset, as dictated by α, results in two main inconveniences. First, shifting the function further away from the cluster increases the chance of accidently covering the lower tails of a neighboring (intruder) cluster. Second, high α values inevitably come at the expense of decreasing the radial resolution close to the centroid of the cluster, something that is further accentuated by the increased σ of the displaced SF, and which can decrease the quality of the resultant description.

**Figure 10:**
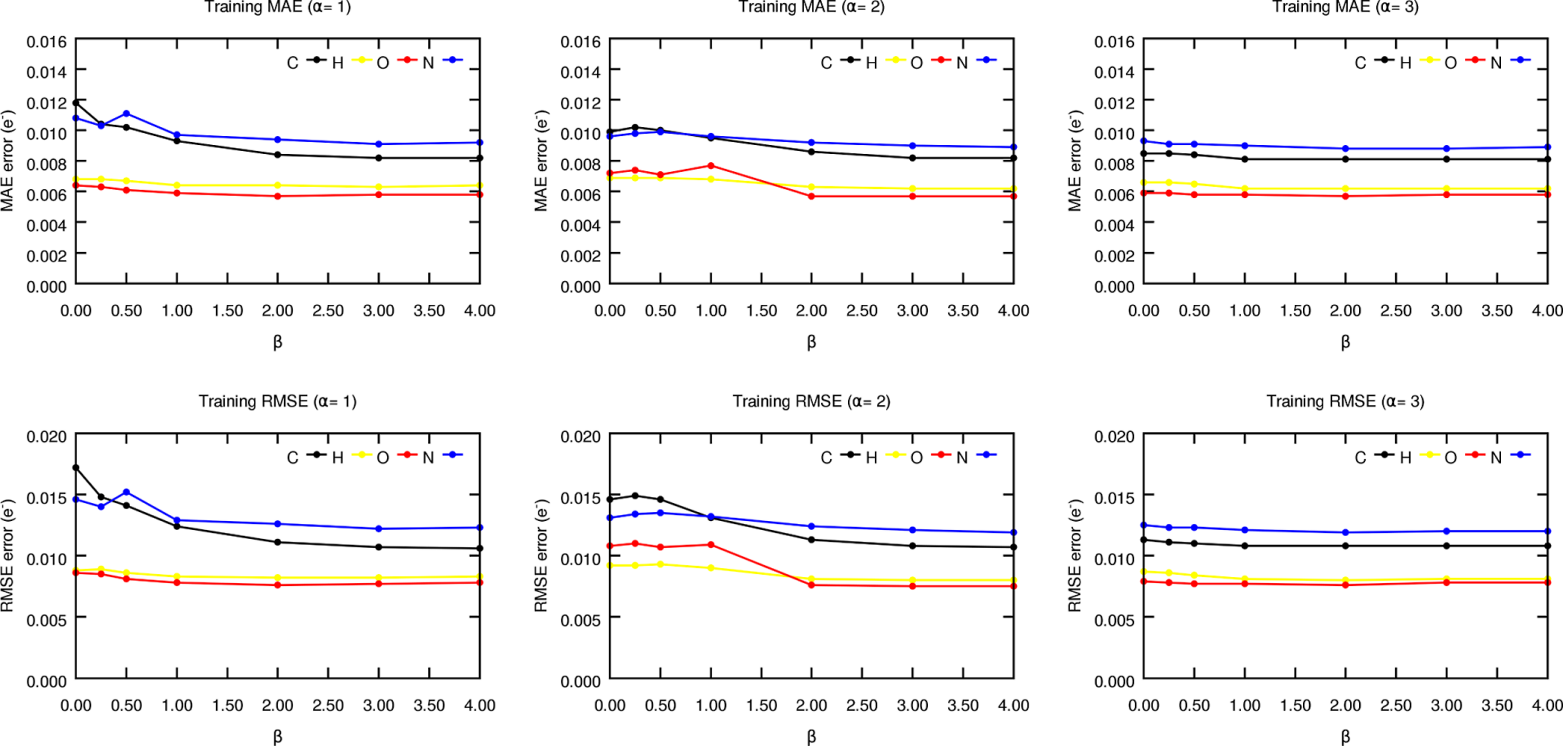
Evolution of the training MAE and RMSE metrics, in electrons, of the FFNN models trained to predict the atomic charges of capped alanine as a function of the α and β values. The results for the testing subset are gathered in Figure S18.

**Figure 11:**
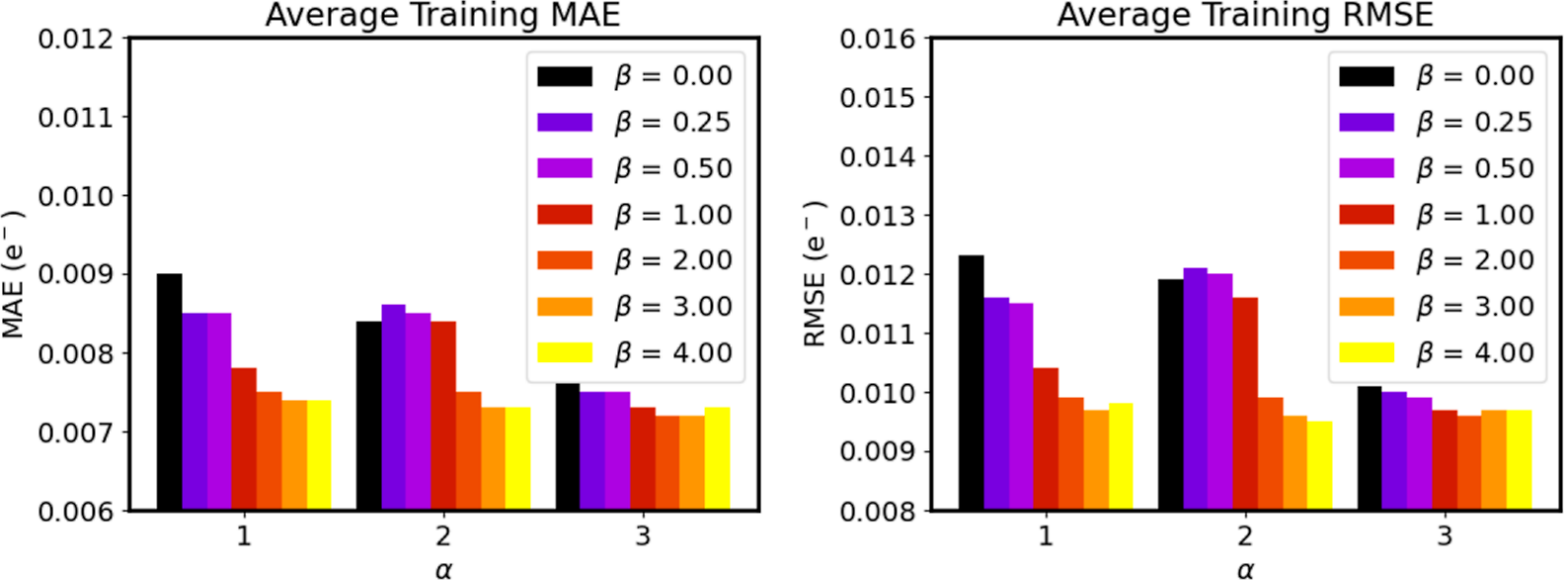
Evolution of the mean training MAE and RMSE error metrics, in electrons, of the FFNN models trained to predict the atomic charges of capped alanine as a function of the α and β values employed. The results for the testing subset are gathered in Figure S23.

We now explore the effect of using the binary distribution scheme in the performance of the models, as shown in Figure 10. Although different elements seem to behave in slightly different ways (e.g., H and O atoms show more monotonous trends) due to the widely varied feature and property distributions, some general trends can be distilled from the data. In agreement with the behavior found for the displaced functions, the errors tend to generally decrease as the value of α increases, something that becomes even more obvious from Figure 11, which shows the mean error metrics regardless of the chemical nature of the atom. However, more complex trends are found for the β parameter. For instance, for low α, monotonously increasing the value of β decreases the prediction errors, because larger widths can be safely used without excessive contamination from the neighboring clusters. On the other hand, for optimum offsets (α = 3), the prediction errors exhibit a convergent trend with β as reflected by the quasi-convex behavior found for the mean model performance, see Figure 11. These findings, especially prominent for the C atom, could arise from the conspiring interplay between two opposite effects: initially, increasing the β values helps the description of those bond distances close to equilibrium, decreasing the errors. However, further increasing the width of the Gaussian reduces its specificity and resolution, hampering its performance. Altogether, it seems that the combination of α = 3 and a moderate β value (between 1 and 2) affords the best average results. Indeed, the FFNN models trained on the latter seem to achieve the maximum radial resolution threshold (see Table S9 in the Supporting Information), with MAE errors of 0.008, 0.006, 0.006, and 0.009 electrons for C, H, O, and N, respectively. It is remarkable that these decent prediction accuracies are achieved while solely relying on radial information, and suggests that the property under study may be well characterized by at most two-body information.

### Effect of Including Three-Body Information

**Figure 12:**
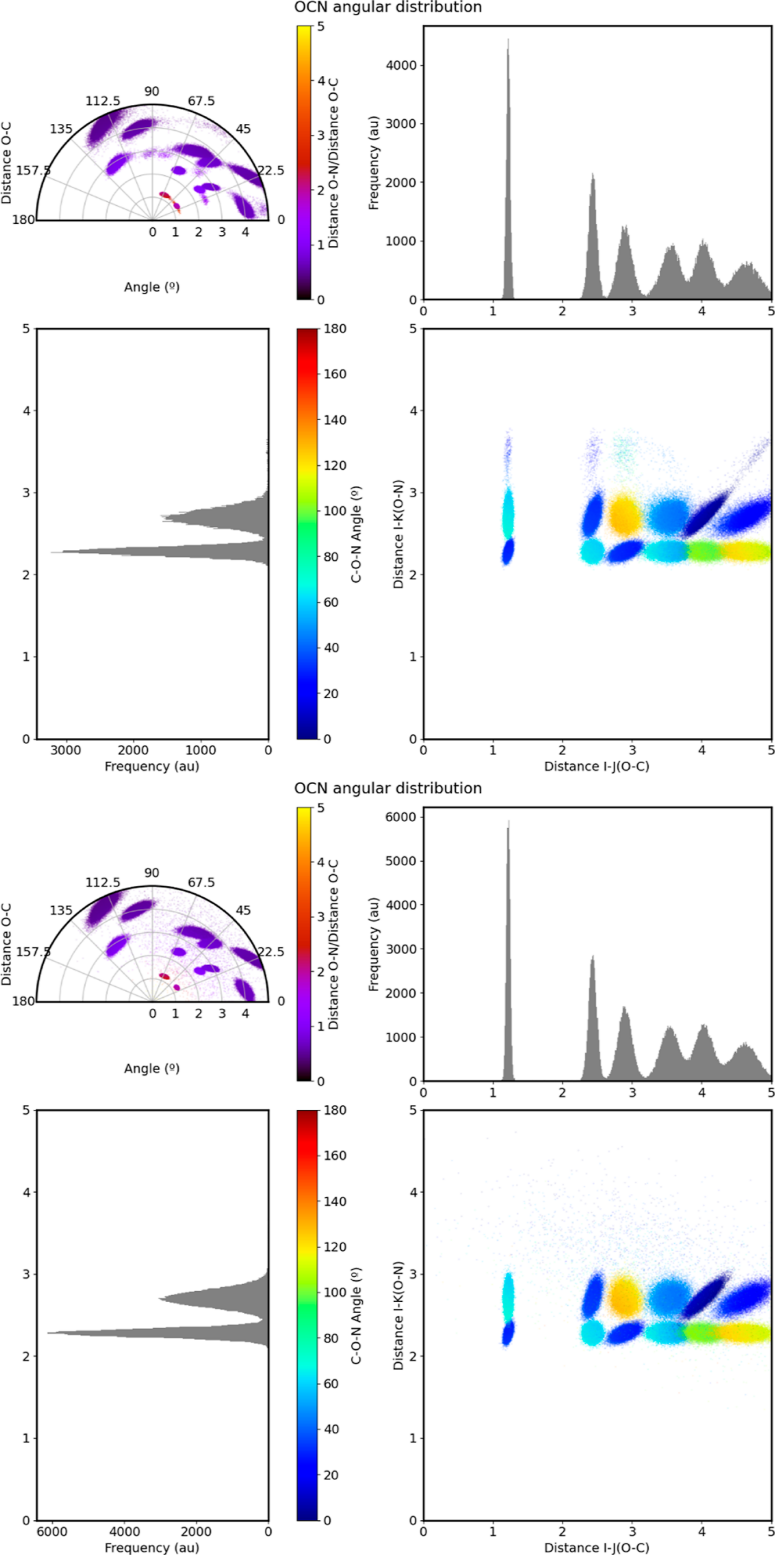
Observed (left) and GMM reconstructed (right) angular distribution of the O–CN atomic trio of capped alanine. The radial dispersion plot is colored according to the θ_*ijk*_ values, whereas the polar plot is colored according to the *r*_*ik*_/*r*_*ij*_ ratio to show the heterogeneity in the neighboring pairwise distances.

After having evaluated the performance of different schemes in the optimization of radial symmetry functions, we now explore the impact of including three-body information in the models, paying special attention to the quality of the optimized angular symmetry functions. Figure 12 shows the observed and GMM-reconstructed angular distributions for the O–CN atomic trio (gathering the contribution of CN atomic pairs to the chemical environments of O atoms) in capped alanine. From such distributions, it is evident that the proposed unsupervised learning model is able to extract the intrinsic features giving rise to the observed chemical environments, resulting in well-defined clusters. Even a quick analysis of the latter provides fruitful insight into the chemistry of the system, revealing patterns such as that associated with the O–C–N peptidic bond. We are thus tempted to think that, other than in the construction of ML features, these clustering techniques may find use in broader, more general applications, worth studying in the near future.

**Figure 13:**
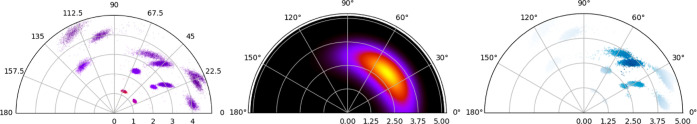
Reconstructed O–CN angular distribution (left), activation of one of the O–CN angular symmetry functions (center) along with the contribution of each cluster to the latter (right). A low weight symmetry function is shown. White and blue colors are used to map the local activation within a uniform scale from 0 to 1, respectively. For the radial dimension, the average value of the *r*_*ij*_ and *r*_*ik*_ features is used.

**Figure 14:**
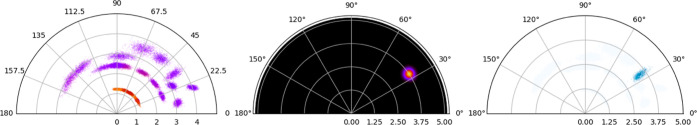
Reconstructed O–CC angular distribution (left), activation of one of the O–CC angular symmetry functions (center) along with the contribution of each cluster to the latter (right). A high weight symmetry function is shown. White and blue colors are used to map the local activation within a uniform scale from 0 to 1, respectively. For the radial dimension, the average value of the *r*_*ij*_ and *r*_*ik*_ features is used.

Further information about the quality of the resultant features can be distilled by analyzing their general topology and how different clusters contribute to the latter, which can be easily obtained with the built-in utilities of our code. Figure 13 presents one of the optimized angular symmetry functions of the (low-weight) O–CN atomic trio, while Figure 14 shows the same for the (high-weight) O–CC atomic trio. Each figure also displays the individual contributions of each cluster. Because the GMM clusters are sorted by weight, the latest functions of each set would generally arise from ill-defined collections of data, characterized by relatively high variances in the angular and radial domains and thus showing a limited specificity. Indeed, this effect is clearly reflected in the individual contributions of each cluster to the activation of the symmetry functions (as shown in the right panels of Figures 13 and 14). For instance, the lightweight O–CN function spreads noticeably throughout the spatial domain and becomes activated by a collection of different clusters. Diametrically opposed trends are found for the O–CC function, which is highly specific and is solely activated by a single cluster of the sample pool. These findings, coupled to the large amount of angular functions that often emerge from the GMM clustering, suggest that it may be convenient to use only the top fraction of the optimized angular features. The latter should generally correspond to highly specific functions accounting for the vast majority of the observed chemical environments of the system.

We will now check the effect of including 3-body information in the input features through the angular ACSF functions. Given the combinatorial growth of the number of possibilities with the size of the atomic groups, the geometries were retrieved every 20 steps. The convergence thresholds were set to 30 and 40% for the BIC score and its gradient, respectively. As for the radial functions, the best-performing features (binary, α = 3, β = 1) were employed in combination with the angular functions to create the corresponding AEVs. Different combinations of cutoff radius (*r*_c_), fraction of selected functions (afrac), and maximum number of GMM clusters (nmax) were explored to evaluate the impact of the latter parameters on the quality of the resultant description of the angular environments.

**Figure 15:**
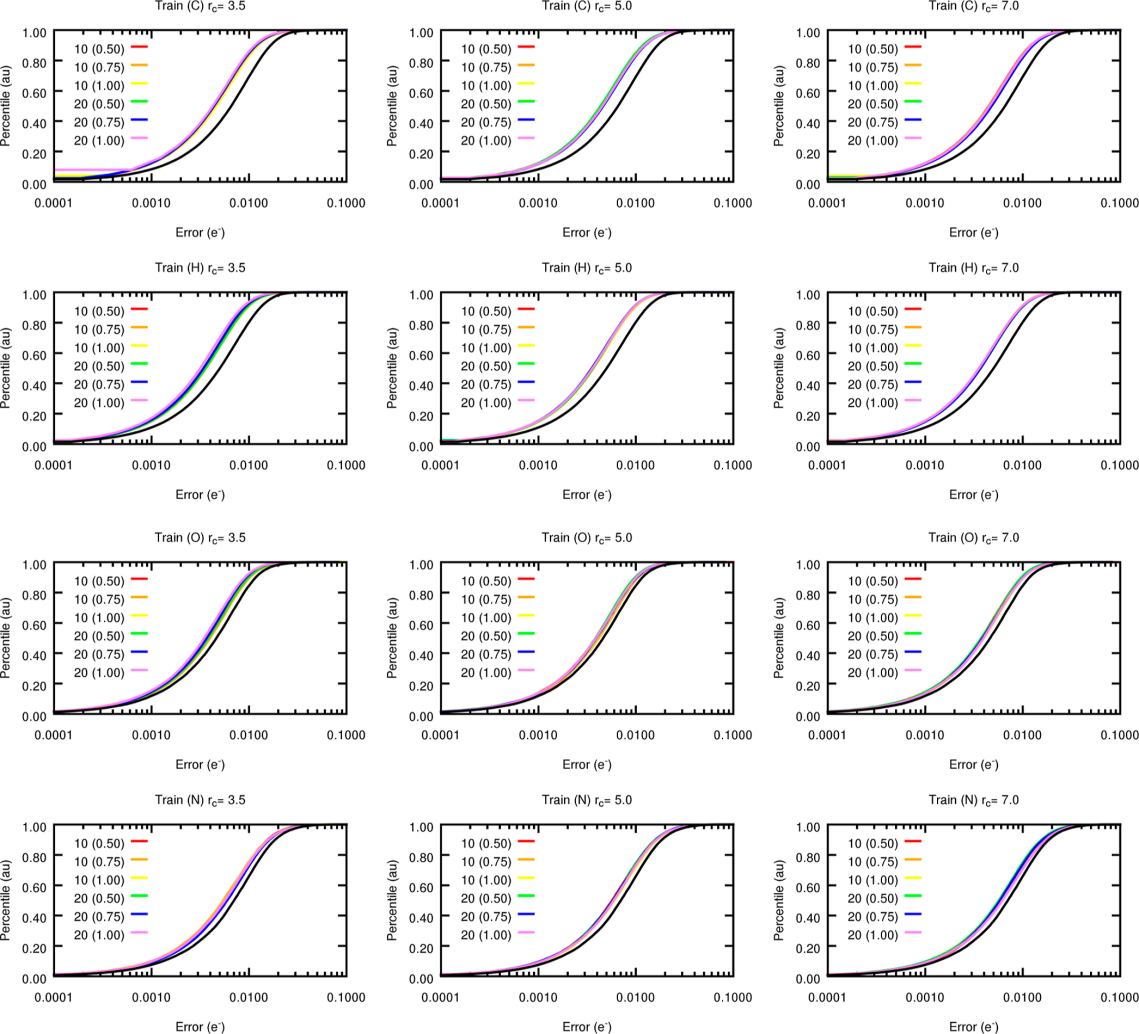
S-curves for the FFNN predicted atomic charges of capped alanine using the optimized radial and angular symmetry functions. A fixed collection of radial functions (binary, α = 3, β = 1) was employed in combination with a varying set of angular terms optimized using different nmax, afrac, and *r*_c_ values. All values are given in electrons. For the sake of comparison, the S-curves for the models trained exclusively on the binary radial functions (black curve) are also shown. The cutoff radius (*r*_c_) is reported in Å. The different colors correspond to the combination of increasing values of nmax and afrac (shown between brackets) for a given *r*_c_. The results for the testing subset are gathered in Figure S24.

The S-curves for the FFNN models trained by using such a combination of radial and angular symmetry functions are collected in Figure 15. It is clear that accounting for angular terms results in a subtle, yet noticeable, increase in prediction accuracy. Such a trend, evidenced by the S-curves shifting to the left compared to the reference values (shown in black), is slightly more pronounced for C and H atoms, for which the MAE decreases by about 0.002 electrons on average (see Section S6 in Supporting Information). It is only in the case of the nitrogen atoms where a smaller improvement of about 0.001 electrons is achieved, suggesting that the angular terms contribute to a lower extent to the characterization of their local chemical environments. This discrepancy is likely to arise from the very homogeneous environments sampled by the nitrogen atoms in capped alanine (only two atoms are present and both are chemically equivalent to one another).

As for the effect of the parameters employed in the angular feature selection, it is difficult to draw a clear conclusion owing to the similar performance exhibited by all of the angular terms. In fact, nmax or afrac values have little to no impact (0.001 electrons at most) on the raw performance of the models, yielding slightly better performing models for nmax = 20 and afrac = 1.0. In agreement with our previous hypothesis, these results show that most of the valuable chemical information characterizing the target property under study is likely to be encoded in the radial symmetry functions, which would reduce the effect of the number of angular terms on the overall quality of the description. Finally, setting the cutoff radius to relatively small values (e.g., 3.5 Å) seems to benefit the prediction accuracy, as evidenced by a slight right-shift of the S-curves when moving from the left to the right panels of Figure 15. Such a trend, in agreement with chemical intuition, points out that the effects of three-body terms to the target property (atomic charges) are more local than the pairwise contributions. It should be highlighted that, even when using simple FFNN models, the symmetry functions optimized here allow to achieve fairly decent prediction accuracies, which prove the feasibility of our approach in the precise description of the local chemical environments. We briefly demonstrate in the next section that the latter accuracies, in the range of 0.004–0.006 electrons on average (see Section S6 in the Supporting Information), improve even further when these features are used in conjunction with better performing ML architecture, such as GPs, trained in FEREBUS.

**Figure 16:**
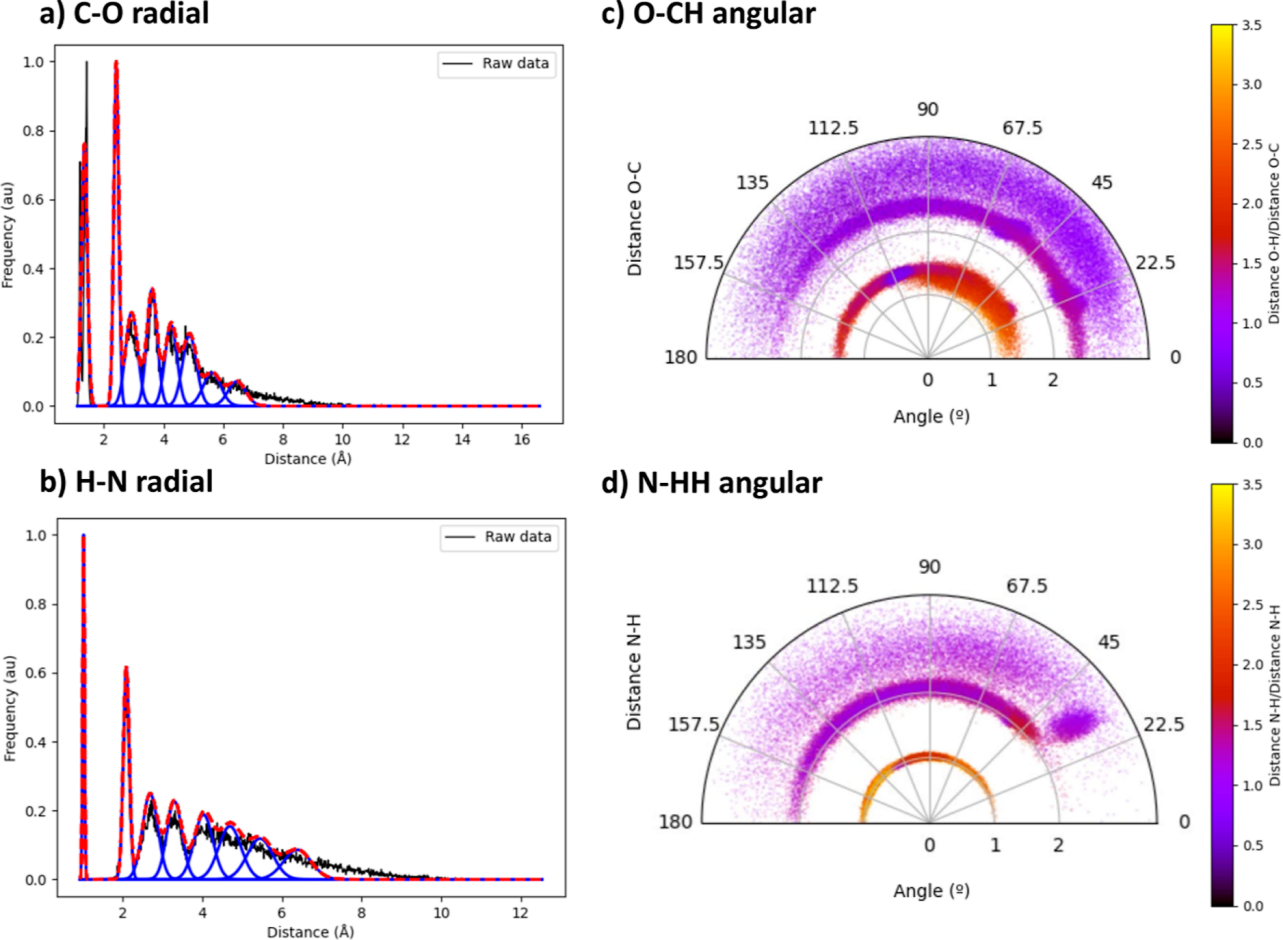
Representation of a selection of the radial and angular clusters identified by the GMM clustering across a random subset of the CHON space, as represented by the NNAIMQ database.^37,43^ For the radial environments, the black and red curves show the observed and reconstructed distributions, the latter arising from a collection of the individually identified clusters, in blue. Further details on this database along with the parameters employed for the clusterization of the radial and angular spaces are collected in the Supporting Information, Section S9.

Finally, we conclude this section by demonstrating the quality of our automatically constructed features compared to those obtained through laborious conventional selection. To achieve this, we utilize the NNAIMQ database, recently introduced by some of us.^37^ The latter gathers about 46,000 C, H, O, and N containing molecules from the equilibrium and near-equilibrium CHON chemical space. For each of these molecules, a collection of fine-tuned features is provided. As such, this represents an ideal scenario to test the quality of our self-tuned descriptors, as well as the ability of our approach to explore the vastness of the chemical space encompassing different chemical compounds. Using a small fraction (less than 10%) of the NNAIMQ database^43^ (see Supporting Information Section S9), our code was employed to explore the CHON chemical space, as shown in Figure 16. The latter reveals very distinct features in the two- and three-body distributions, showcasing well-defined clusters at short distances with a prominent dispersion that grows with the separation to the reference atomic position. This finding stems from the prevalence of common functional groups in CHON organic scaffolds, restricting the diversity found across immediate chemical contacts. The radial and angular features arising from the latter were then used to train FFNN models for the prediction of the QTAIM atomic charges of the whole NNAIMQ database. The results, mostly gathered in Supporting Information Section S9, show that the self-tuned features achieve similar performance to the meticulously selected ACSF functions, achieving testing MAEs in the range of 0.008–0.024 electrons. Such a prediction accuracy, close to the 0.007–0.016 electrons range reported in the original work,^37^ showcases the efficacy of our approach in effortlessly selecting high-quality chemical descriptors. This is particularly noteworthy, as we optimized the features using only a limited fraction of the CHON chemical space for computational efficiency, which might result in an underestimation of our approach’s actual performance. Such an effect is notorious in the N predictions as their scarcity in common CHON molecules results in the largest errors, reflecting the challenges in learning from limited data. Despite these considerations, the overall results emphasize the appropriateness of our approach in automating chemical feature creation, offering a significant advancement with potential implications for chemical ML model development.

### Performance Enhancement Using FEREBUS

**Figure 17:**
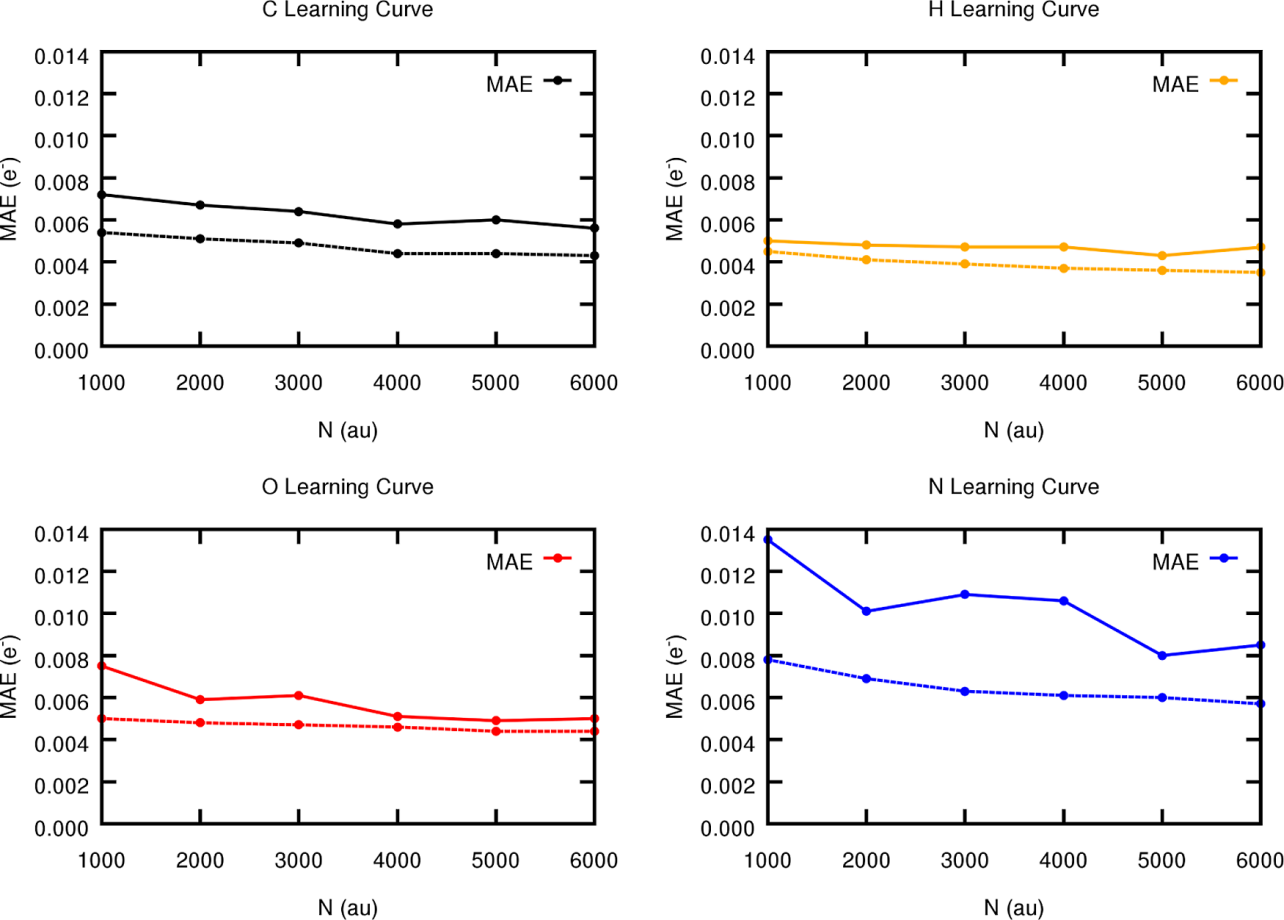
Evolution of the element-wise (testing) MAE metrics for the FFNN (solid) and GP (dashed) models with the number of molecular instances employed for the training. The models were trained on the best performing features (radial binary functions with α = 3 and β = 1 along with angular terms).

Trying to explore the full potential of our finely tuned features, while checking their utility across different architectures, we finally evaluate the performance of the latter with the more sophisticated GP models. For such a purpose, both models were trained, using the best performing radial and angular features, on a varying number of training points (from 1000 to 6000 molecular instances) randomly sampled from the full database. It should be noticed that since FEREBUS naturally builds atom-wise models, the element-wise metrics presented here were derived by averaging over atomic models of the same element. The results are collected in Section S7.2 of the Supporting Information and Figure 17.

**Figure 18:**
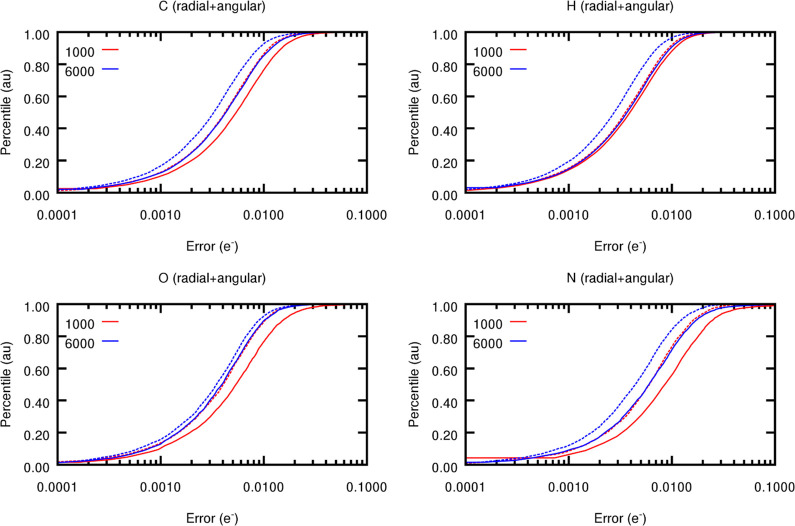
Element-wise (testing) S-curves for FFNN (solid) and GP (dashed) models trained on 1000 and 6000 training points. For GPs, the element-wise performances were computed by averaging out the atom-wise predictions.

Figure 17 gathers the learning curves reported in terms of the MAE metrics and shows that the prediction errors decrease with the number of training points, improving the generalization abilities of the models. As such, and with the particular exception of the nitrogen atoms, which tend to show a more erratic behavior with the FFNN, the prediction accuracy tends to converge with the number of training points. Moreover, the GP models are able to exploit the information contained in the symmetry functions, leading to a significant improvement of the model’s ability to predict the atomic charges. Actually, GPs outperform FFNNs in all the studied cases, a finding that becomes specially pronounced in the low-data regimes^64^ as evidenced from the elemental S-curves, comprised in Figure 18. Indeed, GP models trained on only 1000 points are already competing with their FFNN analogs trained on the whole database (15,000 points). In addition, Tables S13 and S14 in the Supporting Information point out that GP models are able to provide nearly a 2-fold increase in the quality of the predictions, despite being trained on less than half of the training set of FFNN models. These observations not only corroborate the well-known predictive superiority of kernel-based methods over neural networks in the low-data regime^64,65^ but also demonstrate how a good ML architecture can benefit from the excellent quality of the refined ACSFs proposed here. It should be noted that the lower performance of the FFNN models in the low-data regime does not have anything to do with the quality of the features. Instead, it is reminiscent of the overfitting problem, a common downside of neural network architectures, which often goes away with proper regularization such as data augmentation.^66^

As one extends the size of the training set, FFNN models become less prone to overfitting, which is reflected by the sudden improvement in their performance with an increasing number of training points (reflected in the left shift of the S-curves with the number of points, Figure 18). Like FFNNs, the quality of the GP models also improves with data augmentation. Notice, however, the saturation effect that characterizes the learning curve of FFNN models and, to some extent, that of GP models (trained with a 250-point validation set). Many factors might have contributed to this global behavior. These include (without being restricted to): (i) the inherent quality of the data, whose noise level might be imposing an upper limit on the achievable performance, and (ii) the chosen optimization/training settings. Indeed, retraining the GP models with a larger internal validation set was found to slightly improve their performance on the test set, thus providing evidence of the relevance of selecting appropriate training settings. In the case of FEREBUS models, the size and representativeness of the fixed internal validation set often determine the generalization aptitude of every trained model.

## Conclusions

Creating a suitable, machine-compatible array from the intrinsic physical variables defining a chemical system is crucial for the development of chemical AI. In this context, symmetry functions offer an efficient strategy to encode the local chemical environments of an atom embedded in its crystalline or molecular framework. Despite being intuitive and efficient, the accuracy of the resultant description heavily relies on adequate selection of the symmetry function parameters. Although chemical intuition can aid the latter, the vastness of the hyperparameter space makes this fine-tuning a challenging task. Here, we introduce an innovative approach, embedded in an easy-to-use Python code, to automate the optimization of the descriptor parameters. Our strategy, leveraging unsupervised Machine Learning techniques, employs a Gaussian Mixture Model (GMM) to unravel hidden patterns in the intrinsic geometrical features of a molecule. In this way, the chemical space of the molecule, sampled through MD or NMS techniques, is decomposed into a clear-cut collection of well-defined clusters, corresponding to characteristic radial and angular features of the system, which are then used to construct tailor-made symmetry functions. The unsupervised clustering implemented here has proven able to explore both conformational and chemical spaces across a diverse spectrum of molecules, encompassing highly flexible structures as well as notably rigid scaffolds, highlighting the general applicability of our implementation. As for the quality of the technique used to sample the space, unlike MD, NMS is constrained to the vicinity of the initial equilibrium structure, limiting its ability to capture significant conformational shifts. Consequently, NMS may benefit from combining simulations initiated from different potential wells. While high-temperature MD is generally recommended for extensive space sampling, caution is advised in weakly bonded intermolecular systems, as dissociation issues may compromise trajectory representativeness, which can, in turn, jeopardize the applicability of our approach in these scenarios.

The quality of the description provided by the self-tuned features was put to the test with ML estimation of the atomic QTAIM charges of a prototypical amino acid with FFNN and GP models. In this regard, the impact of the spatial distribution of the symmetry functions with respect to the cluster was thoroughly explored through the implementation of three resampling techniques: even, displaced, and binary Gaussian functions. Our findings have shown that breaking the symmetry of the function with respect to the cluster distribution is of utmost importance, resulting in an extraordinarily large increase in the prediction accuracy of the models. Indeed, using a pair of optimally spaced symmetry functions to describe the lower and upper tails of a cluster (binary), MAEs as low as 0.006 electrons have been achieved, even when using only radial information. Beyond two-body terms, GMMs are able to efficiently decompose the considerably more complex angular space from which three-body symmetry functions can be built. Accounting for these angular terms aids the description of local chemical environments reducing the errors by 0.001 to 0.002 electrons. On top of that, our automatically generated descriptors have demonstrated comparable performance to that provided by a meticulously fine-tuned, task-specific feature selection, even when utilizing only a small subset of the entire target space for feature optimization. This is exemplified through the prediction of the atomic charges of the NNAIMQ database,^37^ with errors in the range of 0.008–0.024 electrons, emphasizing the significant contribution of this work to the automation of chemical feature construction. Moreover, the potential of our features can be further improved when used in combination with better performing ML kernels. This is directly reflected by the outstanding performance of some atomic GP models, achieving predictive mean absolute errors as low as 0.002 electrons. As such, our study further demonstrates the effectiveness of these finely tuned features across different architectures, showcasing the superiority of GP models over FFNNs in various scenarios, particularly in low-data regimes. Notably, GP models, trained on a fraction of the data set compared to FFNNs, consistently provide nearly a 2-fold increase in prediction quality, emphasizing the predictive superiority of kernel-based methods in such conditions.

All things considered, our work marks a pivotal step in the ongoing challenge of AI-driven chemical research, paving the way toward the effortless construction of local chemical descriptors. As such, we believe that our innovative approach holds valuable promise for the future of computational chemistry and chemical AI. The automated construction of radial and angular ACSF features opens doors to advancing in this field by eliminating the bias and by reducing the costs associated with the alternative and tedious manual feature selection. Additionally, by seamlessly navigating the conformational and chemical spaces, our strategy emerges as a valuable tool for efficiently assessing the quality of the underlying sampling approach. Finally, the proven success in decomposition of the local chemical environment enables the creation of more precise chemical descriptors. In this way, utilizing GMMs to traverse complex spaces could aid in integrating additional effects that are not easily captured by the conventional combination of radial and angular terms. In fact, our strategy could lead to the development of more accurate and resilient AEVs by expanding pairwise interactions to encompass many-body effects, a path that we aim to explore in the near future. It should be mentioned, however, that maintaining the desired asymmetry as descriptor complexity and dimensionality increase poses a significant challenge in this endeavor.

## Data Availability

The data and code that support the findings of this study will be made available in a GitHub project (https://github.com/m-gallegos/MM2SF).

## Supporting Information Available

Details about the database creation along with an analysis of the chemical space sampled throughout the latter; computational details regrading the training of the ML models used in this work; in-depth view of the algorithmic details of the here presented code; selection of the optimized radial and angular symmetry functions; results for the increase in performance offered by FEREBUS when compared to simple FFNN models; suitability of the NMS and MD samplings of the space, with a particularly emphasis on the effect of the temperature; and applicability of our approach in the exploration of the chemical space, distilling the radial and angular environments arising from a heterogeneous database (PDF)

## References

[ref1] Choudhary N.; Bharti R.; Sharma R. Role of artificial intelligence in chemistry. Mater. Today: Proc. 2022, 48, 1527–1533. 10.1016/j.matpr.2021.09.428.

[ref2] Gasteiger J. Chemistry in Times of Artificial Intelligence. ChemPhysChem 2020, 21, 2233–2242. 10.1002/cphc.202000518. 32808729 PMC7702165

[ref3] Bianchini S.; Müller M.; Pelletier P. Artificial intelligence in science: An emerging general method of invention. Resour. Policy 2022, 51, 104604 10.1016/j.respol.2022.104604.

[ref4] Keith J. A.; Vassilev-Galindo V.; Cheng B.; Chmiela S.; Gastegger M.; Müller K. R.; Tkatchenko A. Combining Machine Learning and Computational Chemistry for Predictive Insights Into Chemical Systems. Chem. Rev. 2021, 121, 9816–9872. 10.1021/acs.chemrev.1c00107. 34232033 PMC8391798

[ref5] Goh G. B.; Hodas N. O.; Vishnu A. Deep learning for computational chemistry. J. Comput. Chem. 2017, 38, 1291–1307. 10.1002/jcc.24764. 28272810

[ref6] Westermayr J.; Gastegger M.; Schütt K. T.; Maurer R. J. Perspective on integrating machine learning into computational chemistry and materials science. J. Chem. Phys. 2021, 154, 154 10.1063/5.0047760. 34241249

[ref7] Manzhos S.; Tsuda S.; Ihara M. Machine learning in computational chemistry: interplay between (non)linearity, basis sets, and dimensionality. Phys. Chem. Chem. Phys. 2023, 25, 1546–1555. 10.1039/D2CP04155C. 36562317

[ref8] Heinen S.; Schwilk M.; von Rudorff G. F.; von Lilienfeld O. A. Machine learning the computational cost of quantum chemistry. Mach. Learn.: Sci. Technol. 2020, 1, 025002 10.1088/2632-2153/ab6ac4.

[ref9] Vamathevan J.; Clark D.; Czodrowski P.; Dunham I.; Ferran E.; Lee G.; Li B.; Madabhushi A.; Shah P.; Spitzer M.; Zhao S. Applications of machine learning in drug discovery and development. Nat. Rev. Drug Discovery 2019, 18, 463–477. 10.1038/s41573-019-0024-5. 30976107 PMC6552674

[ref10] Schütt K. T.; Gastegger M.; Tkatchenko A.; Müller K. R.; Maurer R. J. Unifying machine learning and quantum chemistry with a deep neural network for molecular wavefunctions. Nat. Commun. 2019, 10, 5024 10.1038/s41467-019-12875-2. 31729373 PMC6858523

[ref11] Fang X.; Liu L.; Lei J.; He D.; Zhang S.; Zhou J.; Wang F.; Wu H.; Wang H. Geometry-enhanced molecular representation learning for property prediction. Nat. Mach. Intell. 2022, 4, 127–134. 10.1038/s42256-021-00438-4.

[ref12] Schmidt J.; Marques M. R. G.; Botti S.; Marques M. A. L. Recent advances and applications of machine learning in solid-state materials science. npj Comput. Mater. 2019, 5, 83 10.1038/s41524-019-0221-0.

[ref13] Wiener H. Structural Determination of Paraffin Boiling Points. J. Am. Chem. Soc. 1947, 69, 17–20. 10.1021/ja01193a005. 20291038

[ref14] Raghunathan S.; Priyakumar U. D. Molecular representations for machine learning applications in chemistry. Int. J. Quantum Chem. 2021, 122, 122 10.1002/qua.26870.

[ref15] Seko A.; Togo A.; Tanaka I. Nanoinformatics; Springer Singapore, 2018; pp 3–23.

[ref16] Todeschini R.; Consonni V. Handbook of Molecular Descriptors; Wiley, 2000.

[ref17] Comesana A. E.; Huntington T. T.; Scown C. D.; Niemeyer K. E.; Rapp V. H. A systematic method for selecting molecular descriptors as features when training models for predicting physiochemical properties. Fuel 2022, 321, 123836 10.1016/j.fuel.2022.123836.

[ref18] Klein C. T.; Kaiser D.; Ecker G. Topological Distance Based 3D Descriptors for Use in QSAR and Diversity Analysis. J. Chem. Inf. Comput. Sci. 2004, 44, 200–209. 10.1021/ci0256236. 14741029

[ref19] Rupp M.; Tkatchenko A.; Müller K. R.; von Lilienfeld O. A. Fast and Accurate Modeling of Molecular Atomization Energies with Machine Learning. Phys. Rev. Lett. 2012, 108, 058301 10.1103/PhysRevLett.108.058301. 22400967

[ref20] Morris G. M.; Goodsell D. S.; Halliday R. S.; Huey R.; Hart W. E.; Belew R. K.; Olson A. J. Automated docking using a Lamarckian genetic algorithm and an empirical binding free energy function. J. Comput. Chem. 1998, 19, 1639–1662. 10.1002/(SICI)1096-987X(19981115)19:14<1639::AID-JCC10>3.0.CO;2-B.

[ref21] Morgan H. L. The Generation of a Unique Machine Description for Chemical Structures-A Technique Developed at Chemical Abstracts Service. J. Chem. Doc. 1965, 5, 107–113. 10.1021/c160017a018.

[ref22] Carhart R. E.; Smith D. H.; Venkataraghavan R. Atom pairs as molecular features in structure-activity studies: definition and applications. J. Chem. Inf. Comput. Sci. 1985, 25, 64–73. 10.1021/ci00046a002.

[ref23] Devinyak O.; Havrylyuk D.; Lesyk R. 3D-MoRSE descriptors explained. J. Mol. Graphics Modell. 2014, 54, 194–203. 10.1016/j.jmgm.2014.10.006. 25459771

[ref24] Ferguson A.; Heritage T.; Jonathon P.; Pack S.; Phillips L.; Rogan J.; Snaith P. J. Comput.-Aided Mol. Des. 1997, 11, 143–152. 10.1023/A:1008026308790. 9089432

[ref25] Todeschini R. Perspect. Drug Discov. Des. 1998, 9/11, 355–380. 10.1023/a:1027284627085.

[ref26] Mills M. J. L.; Popelier P. L. A. Electrostatic Forces: Formulas for the First Derivatives of a Polarizable, Anisotropic Electrostatic Potential Energy Function Based on Machine Learning. J. Chem. Theory Comput. 2014, 10, 3840–3856. 10.1021/ct500565g. 26588529

[ref27] Behler J. Atom-centered symmetry functions for constructing high-dimensional neural network potentials. J. Chem. Phys. 2011, 134, 074106 10.1063/1.3553717. 21341827

[ref28] Bartók A. P.; Kondor R.; Csányi G. On representing chemical environments. Phys. Rev. B 2013, 87, 184115 10.1103/PhysRevB.87.184115.

[ref29] Hansen K.; Biegler F.; Ramakrishnan R.; Pronobis W.; von Lilienfeld O. A.; Müller K. R.; Tkatchenko A. Machine Learning Predictions of Molecular Properties: Accurate Many-Body Potentials and Nonlocality in Chemical Space. J. Phys. Chem. Lett. 2015, 6, 2326–2331. 10.1021/acs.jpclett.5b00831. 26113956 PMC4476293

[ref30] Schütt K. T.; Kindermans P.-J.; Sauceda H. E.; Chmiela S.; Tkatchenko A.; Müller K.-R. SchNet: A Continuous-Filter Convolutional Neural Network for Modeling Quantum Interactions; Curran Associates, Inc., 2017.

[ref31] Schütt K. T.; Sauceda H. E.; Kindermans P.-J.; Tkatchenko A.; Müller K. R. SchNet – A deep learning architecture for molecules and materials. J. Chem. Phys. 2018, 148, 241722 10.1063/1.5019779. 29960322

[ref32] Gastegger M.; Schwiedrzik L.; Bittermann M.; Berzsenyi F.; Marquetand P. wACSF—Weighted atom-centered symmetry functions as descriptors in machine learning potentials. J. Chem. Phys. 2018, 148, 241709 10.1063/1.5019667. 29960372

[ref33] Bircher M. P.; Singraber A.; Dellago C. Improved Description of Atomic Environments using Low-cost Polynomial Functions with Compact Support. Mach. Learn.: Sci. Technol. 2021, 2, 035026 10.1088/2632-2153/abf817.

[ref34] Zhang K.; Yin L.; Liu G. Physically inspired atom-centered symmetry functions for the construction of high dimensional neural network potential energy surfaces. Comput. Mater. Sci. 2021, 186, 110071 10.1016/j.commatsci.2020.110071.

[ref35] Eckhoff M.; Behler J. High-dimensional neural network potentials for magnetic systems using spin-dependent atom-centered symmetry functions. npj Comput. Mater. 2021, 7, 170 10.1038/s41524-021-00636-z.

[ref36] Unke O. T.; Meuwly M. PhysNet: A Neural Network for Predicting Energies, Forces, Dipole Moments, and Partial Charges. J. Chem. Theory Comput. 2019, 15, 3678–3693. 10.1021/acs.jctc.9b00181. 31042390

[ref37] Gallegos M.; Guevara-Vela J. M.; Martín Pendás Á. NNAIMQ: A neural network model for predicting QTAIM charges. J. Chem. Phys. 2022, 156, 014112 10.1063/5.0076896. 34998318

[ref38] Rowe P.; Deringer V. L.; Gasparotto P.; Csányi G.; Michaelides A. An accurate and transferable machine learning potential for carbon. J. Chem. Phys. 2020, 153, 034702 10.1063/5.0005084. 32716159

[ref39] Hellström M.; Behler J. Structure of aqueous NaOH solutions: insights from neural-network-based molecular dynamics simulations. Phys. Chem. Chem. Phys. 2017, 19, 82–96. 10.1039/C6CP06547C. 27805193

[ref40] Artrith N.; Urban A. An implementation of artificial neural-network potentials for atomistic materials simulations: Performance for TiO2. Comput. Mater. Sci. 2016, 114, 135–150. 10.1016/j.commatsci.2015.11.047.

[ref41] Mudassir M. W.; Goverapet Srinivasan S.; Mynam M.; Rai B. Systematic Identification of Atom-Centered Symmetry Functions for the Development of Neural Network Potentials. J. Phys. Chem. A 2022, 126, 8337–8347. 10.1021/acs.jpca.2c04508. 36300823

[ref42] Bader R. Atoms in Molecules: A Quantum Theory; International Series of Monographs on Chemistry; Oxford University Press: Oxford, 1990.

[ref43] Gallegos M. NNAIMQ-DATA. 2021; https://data.mendeley.com/datasets/tcj2btw63r/1, was accessed on April 6, 2023.

[ref44] Smith J. S.; Isayev O.; Roitberg A. E. ANI-1: an extensible neural network potential with DFT accuracy at force field computational cost. Chem. Sci. 2017, 8, 3192–3203. 10.1039/C6SC05720A. 28507695 PMC5414547

[ref45] Mixture Models and Applications; Bouguila N., Fan W., Eds.; Springer International Publishing, 2020.

[ref46] Fraley C.; Raftery A. E. Model-Based Clustering, Discriminant Analysis, and Density Estimation. J. Am. Stat. Assoc. 2002, 97, 611–631. 10.1198/016214502760047131.

[ref47] Klem H.; Hocky G. M.; McCullagh M. Size-and-Shape Space Gaussian Mixture Models for Structural Clustering of Molecular Dynamics Trajectories. J. Chem. Theory Comput. 2022, 18, 3218–3230. 10.1021/acs.jctc.1c01290. 35483073 PMC9228201

[ref48] Wu X.; Zheng Y.; Zhang J.; Wu B.; Wang S.; Tian Y.; Li J.; Meng X. Investigating Hydrochemical Groundwater Processes in an Inland Agricultural Area with Limited Data: A Clustering Approach. Water 2017, 9, 723 10.3390/w9090723.

[ref49] Blair B.; Chen C.; Glenn A.; Kaplan A.; Ruz J.; Simms L.; Wurtz R. Gaussian mixture models as automated particle classifiers for fast neutron detectors. Stat. Anal. Data Min. 2019, 12, 479–488. 10.1002/sam.11432.

[ref50] Poater J.; Fradera X.; Duran M.; Solà M. The Delocalization Index as an Electronic Aromaticity Criterion: Application to a Series of Planar Polycyclic Aromatic Hydrocarbons. Chem.—Eur. J. 2003, 9, 400–406. 10.1002/chem.200390041. 12532288

[ref51] Outeiral C.; Vincent M. A.; Martín Pendás Á.; Popelier P. L. A. Revitalizing the concept of bond order through delocalization measures in real space. Chem. Sci. 2018, 9, 5517–5529. 10.1039/C8SC01338A. 30061983 PMC6049528

[ref52] Bader R. F. W.; Matta C. F. Atomic Charges Are Measurable Quantum Expectation Values: A Rebuttal of Criticisms of QTAIM Charges. J. Phys. Chem. A 2004, 108, 8385–8394. 10.1021/jp0482666.

[ref53] Pembere A. M. S.; Liu X.; Ding W.; Luo Z. How Partial Atomic Charges and Bonding Orbitals Affect the Reactivity of Aluminum Clusters with Water?. J. Phys. Chem. A 2018, 122, 3107–3114. 10.1021/acs.jpca.7b10635. 29526102

[ref54] Iwaoka M.; Komatsu H.; Katsuda T.; Tomoda S. Nature of Nonbonded Se ··· O Interactions Characterized by 17O NMR Spectroscopy and NBO and AIM Analyses. J. Am. Chem. Soc. 2004, 126, 5309–5317. 10.1021/ja049690n. 15099116

[ref55] Geidl S.; Bouchal T.; Raček T.; Svobodová Vařeková R.; Hejret V.; Křenek A.; Abagyan R.; Koča J. High-quality and universal empirical atomic charges for chemoinformatics applications. J. Cheminf. 2015, 7, 59 10.1186/s13321-015-0107-1. PMC4667495 26633997

[ref56] Di Pasquale N.; Bane M.; Davie S. J.; Popelier P. L. A. FEREBUS: Highly parallelized engine for kriging training: FEREBUS: Highly Parallelized Engine. J. Comput. Chem. 2016, 37, 2606–2616. 10.1002/jcc.24486. 27649926

[ref57] Fletcher T. L.; Davie S. J.; Popelier P. L. A. Prediction of Intramolecular Polarization of Aromatic Amino Acids Using Kriging Machine Learning. J. Chem. Theory Comput. 2014, 10, 3708–3719. 10.1021/ct500416k. 26588516

[ref58] Burn M. J.; Popelier P. L. FEREBUS: a high-performance modern Gaussian process regression engine. Digital Discovery 2023, 2, 152–164. 10.1039/D2DD00082B.

[ref59] Isamura B. K.; Popelier P. L. Toward a simple yet efficient cost function for the optimization of Gaussian process regression model hyperparameters. AIP Adv. 2023, 13, 095202 10.1063/5.0151033.

[ref60] Frisch M. J.; Trucks G. W.; Schlegel H. B.; Scuseria G. E.; Robb M. A.; Cheeseman J. R.; Scalmani G.; Barone V.; Mennucci B.; Petersson G. A.; Nakatsuji H.; Caricato M.; Li X.; Hratchian H. P.; Izmaylov A. F.; Bloino J.; Zheng G.; Sonnenberg J. L.; Hada M.; Ehara M.; Toyota K.; Fukuda R.; Hasegawa J.; Ishida M.; Nakajima T.; Honda Y.; Kitao O.; Nakai H.; Vreven T.; Montgomery J. A. Jr.; Peralta J. E.; Ogliaro F.; Bearpark M.; Heyd J. J.; Brothers E.; Kudin K. N.; Staroverov V. N.; Kobayashi R.; Normand J.; Raghavachari K.; Rendell A.; Burant J. C.; Iyengar S. S.; Tomasi J.; Cossi M.; Rega N.; Millam J. M.; Klene M.; Knox J. E.; Cross J. B.; Bakken V.; Adamo C.; Jaramillo J.; Gomperts R.; Stratmann R. E.; Yazyev O.; Austin A. J.; Cammi R.; Pomelli C.; Ochterski J. W.; Martin R. L.; Morokuma K.; Zakrzewski V. G.; Voth G. A.; Salvador P.; Dannenberg J. J.; Dapprich S.; Daniels A. D.; Farkas O.; Foresman J. B.; Ortiz J. V.; Cioslowski J.; Fox D. J. Gaussian 09. Revision E.01; Gaussian Inc.: Wallingford CT, 2009.

[ref61] Case D. A.; Aktulga H. M.; Belfon K.; Ben-Shalom I. Y.; Berryman J. T.; Brozell S. R.; Cerutti D. S.; Cheatham T. E. III; Cisneros G. A.; Cruzeiro V. W. D.; Darden T. A.; Duke R. E.; Giambasu G.; Gilson M. K.; Gohlke H.; Goetz A. W.; Harris R.; Izadi S.; Izmailov S. A.; Kasavajhala K.; Kaymak M. C.; King E.; Kovalenko A.; Kurtzman T.; Lee T. S.; LeGrand S.; Li P.; Lin C.; Liu J.; Luchko T.; Luo R.; Machado M.; Man V.; Manathunga M.; Merz K. M.; Miao Y.; Mikhailovskii O.; Monard G.; Nguyen H.; O’Hearn K. A.; Onufriev A.; Pan F.; Pantano S.; Qi R.; Rahnamoun A.; Roe D. R.; Roitberg A.; Sagui C.; Schott-Verdugo S.; Shajan A.; Shen J.; Simmerling C. L.; Skrynnikov N. R.; Smith J.; Swails J.; Walker R. C.; Wang J.; Wang J.; Wei H.; Wolf R. M.; Wu X.; Xiong Y.; Xue Y.; York D. M.; Zhao S.; Kollman P. A. Amber 2022; University of California: San Francisco, 2022.

[ref62] Keith T. A. AIMALL; TK Gristmill Software; Overland Park KS: USA, 2019.

[ref63] Jmol: an open-source Java viewer for chemical structures in 3D. http://www.jmol.org/.

[ref64] Handley C. M.; Hawe G. I.; Kell D. B.; Popelier P. L. A. Optimal construction of a fast and accurate polarisable water potential based on multipole moments trained by machine learning. Phys. Chem. Chem. Phys. 2009, 11, 6365 10.1039/b905748j. 19809668

[ref65] Kamath A.; Vargas-Hernández R. A.; Krems R. V.; Carrington T.; Manzhos S. Neural networks vs Gaussian process regression for representing potential energy surfaces: A comparative study of fit quality and vibrational spectrum accuracy. J. Chem. Phys. 2018, 148, 241702 10.1063/1.5003074. 29960346

[ref66] Oyedotun O. K.; Olaniyi E. O.; Khashman A. A simple and practical review of over-fitting in neural network learning. Int. J. Appl. Pattern Recognit. 2017, 4, 307–328. 10.1504/IJAPR.2017.089384.

