# Supplementary material for: An Unsupervised Machine Learning Approach for the Automatic Construction of Local Chemical Descriptors
Source: J Chem Inf Model. 2024 Mar 18;64(8):3059–79. doi: 10.1021/acs.jcim.3c01906 (PMC11040729; doi:10.1021/acs.jcim.3c01906)
Supplement: Supplementary file 1 — ci3c01906_si_001.pdf [file ci3c01906_si_001.pdf]

Supporting Information

An Unsupervised Machine Learning Approach for the Automatic Construction of Local Chemical Descriptors

Miguel Gallegos[1], Bienfait Kabuyaya Isamura[2], Paul L. A. Popelier[*2], and Ángel Martín Pendás[*1]

[1]*Department of Analytical and Physical Chemistry, University of Oviedo, E-33006, Oviedo, Spain.*
[2]*Department of Chemistry, The University of Manchester, Oxford Road, Manchester M13 9PL, United Kingdom*

E-mail: pla@manchester.ac.uk ampendas@uniovi.es

Contents

1 Database creation **S3**

2 Mist plots and the Kabsch algorithm **S4**

3 Training of the ML models **S5**

4 Algorithmic details **S6**
- 4.1 Cutoff function . . . S6
- 4.2 Optimization of the ideal number of GMM clusters . . . S6
- 4.3 Automatic selection of radial ACSF functions . . . S7
  - 4.3.1 Evenly distributed symmetry functions . . . S10
- 4.4 Automatic selection of angular ACSF functions . . . S11
  - 4.4.1 Dealing with the angular redundancies . . . S16
- 4.5 Resampling techniques . . . S20

5 Optimized Radial Symmetry Functions **S22**
- 5.1 GMM clustering of the radial environments . . . S22
- 5.2 Decomposed functions . . . S26
- 5.3 Evenly sampled functions . . . S28
- 5.4 Displaced functions . . . S29
- 5.5 Binary functions . . . S30

6 Optimized Angular Symmetry Functions **S36**

7 Performance enhancement using FEREBUS **S39**
- 7.1 Computational details and methodology: FEREBUS . . . S39
- 7.2 Performance metrics . . . S42

8 Impact of the sampling procedure **S44**
- 8.1 Normal Mode Sampling (NMS) . . . S44
- 8.2 Molecular Dynamics vs Normal Mode Sampling . . . S45
- 8.3 The effect of temperature . . . S53

9 Identifying radial and angular environments in diverse chemical spaces **S60**
- 9.1 GMM exploration of the CHON chemical space . . . S61
- 9.2 Performance of the self-optimized features . . . S69

1 Database creation

Starting from the resultant 20,000 individual snapshots, single point calculations were performed at the B3LYP/6-31+G(d,p) level of theory in the gas phase to obtain the corresponding wavefunctions with the aid of Gaussian09.[1] Then Quantum Theory of Atoms in Molecules (QTAIM) electronic metrics (partial charges along with the localized and delocalized electron counts) were computed for each of the latter geometries with the AIMAll[2] and PROMOLDEN codes.[3] In order to ensure the quality of the reference data used to train the models, potential outliers were removed from the previously computed data points using the following strategy:

- The distribution of the total electronic energies is computed for all the data-points from which the mean, μ, and standard deviation, σ, of the whole population are obtained. Those geometries with energies beyond the $\mu \pm 3 \cdot \sigma$ thresholds or those for which the virial ratio exhibits an offset larger than 0.1 were discarded. Doing so ensures that the database corresponds to a uniformly and homogeneously sampled region of the potential energy landscape.

- The second filtering step relies on the actual target properties to be predicted by the models. Since in our particular scenario we will be dealing with QTAIM electron metrics, such as the partial charges or the localized and delocalized electron counts, special attention was paid to the reconstruction of the corresponding molecular observables. Hence, the distributions of the molecular charges and total electron counts were computed for all the data instances. Those geometries beyond the $\mu \pm 2 \cdot \sigma$ thresholds in the distribution of molecular charge and electron counts, or those which do not satisfy the Poincaré–Hopf theorem, were removed. Notice that since these are directly related to the target properties that will be fed to the models, a tighter outlier criterion has been used when compared to the molecular energies. We note in passing that this filter was applied twice as there were a couple of extreme outliers in the database which could potentially shift the statistics of the distribution, and thus result in a biased picture of the dataset properties.

After removing all the problematic datapoints, 18,950 valid molecular instances were left from the starting 20,000 points.

2 Mist plots and the Kabsch algorithm

The Kabsch algorithm,[4] commonly employed in the context of computational chemistry and cheminformatics, maximizes the overlap between two consecutive frames (e.g molecular geometries) to superimpose their corresponding structures. For such a purpose, the characteristic coordinates of each frame are translated and rotated to minimize a given distance metric, usually the root-mean-square deviation (RMSD), with respect to a reference, something which is commonly achieved through the following steps:

- The structures are displaced to superimpose their corresponding centroids with respect to the reference coordinates.
- The covariance matrix between both structures is then computed.
- Finally, the optimum rotation matrix is found from the previously computed covariance, something which is often obtained by means of the application of a Single Value Decomposition (SVD) to the latter.

Applying such an algorithm to the collection of frames arising from a Molecular Dynamics simulation and superimposing the resultant structures within a single molecular representation allows to obtain an unbiased representation of the relative displacement of the molecular scaffold. Thus, the resultant representation, commonly referred to as *mist plot*, provides a robust way to analyze the extent and rigor with which the potential energy surface is sampled. The following figure shows the *mist plots*, centered at the C3 atom of each frame, of the Molecular Dynamics simulation of peptide-capped alanine. For the sake of convenience, the data for 1000 randomly sampled geometries, out of the 20,000, are shown.

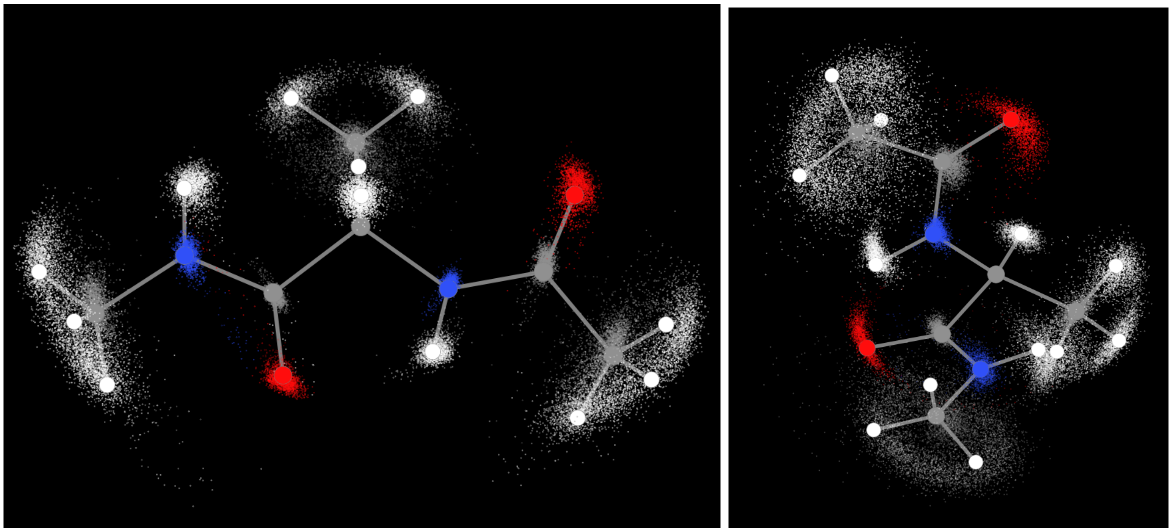

Figure 1: *Mist plots* (centered at the central C3 atom) of the Molecular Dynamics simulation of peptide-capped alanine. For the sake of convenience 1000 geometries randomly sampled from the total pool of 20,000 are shown. The Kabsch algorithm was applied on the whole collection of particles of each molecular frame. Images generated with Matplotlib[5].

3 Training of the ML models

Feed Forward Neural Networks (FFNN), densely connected, were employed as main Machine Learning (ML) architectures for the prediction of the target properties under consideration. Unless otherwise specified, the model architecture was built from 3 layers interfaced through hyperbolic tangent activations, as implemented in Keras.[6] On the other hand, the final output of the model was obtained through the linear combination of the last layer, using thus a linear activation function for the latter. A fixed learning rate of 0.0001 was employed in combination with the RMSprop optimizer, used as default in Keras.[6] The Mean Absolute Error (MAE) of the predictions was used as a kernel for the loss function, while the Root Mean Square Error (RMSE) was also employed, along with the MAE, to track the performance of the model. Finally, the early-stopping approach, with a fixed patience of 25 epochs and using the validation set loss as metric, was employed to ameliorate the potential overfitting of the models. As for the number of neurons, homogeneous arrangements of (10,10,10) or (20,20,20) neurons were used throughout. A standard train-test-val split of the database was employed resulting in 15000, 2000 and 1950 molecular structures for the training, testing and validation subsets, respectively. As for the features, a collection of the introduced here self-tuned ACSF functions (specifically detailed for each case in the upcoming sections as well as throughout the manuscript) were used in all cases. For the sake of convenience, the original scale of each of the features was normalized with respect to the previously specified training subset, resulting in distributions with $\mu = 0$ and $\sigma = 1$ for all the elements of the input vectors fed into the ML models.

All the models were trained with the aid of the Keras[6] and Tensorflow 1.14.0 modules[7] as implemented in Python 3.8. The maximum number of epochs was set to 10^6 although a much smaller number of iterations, set by the early-stopping approach, was commonly required.

Finally, the prediction performance of the trained here models was monitored through different error metrics, namely the Mean Absolute Error (MAE) and the Root Mean Squared Error (RMSE). The MAE is obtained as the mean value of the offset between the observations and the predictions, in absolute value. For a total of n data instances, the MAE is computed as,

$$MAE = \frac{1}{n}\sum_{i=1}^{n}|y_i - \hat{y}_i|, \tag{1}$$

where y_i and $\hat{y}_i$ represent the observed and predicted values for a given data instance, i, respectively.

Likewise, the RMSE error is obtained as the squared difference between the predictions and observations, averaged out over all the data instances in the dataset. For the sake of convenience, the square root of the previous quantity, referred to as the Mean Squared Error (MSE), is often taken, leading to the RMSE. For a total of n data instances, the RMSE is computed in terms of the observed (y_i) and predicted ($\hat{y}_i$) values as,

$$RMSE = \sqrt{\frac{1}{n}\sum_{i=1}^{n}(y_i - \hat{y}_i)^2}. \tag{2}$$

Although both metrics are commonly used in the context of ML, the RMSE is able to penalize more seriously large offsets in the predictions, making it particularly sensitive to the presence of outliers in the data.

4 Algorithmic details

The current section comprises the general details and architecture of the code developed for the automatic optimization of the symmetry functions.

4.1 Cutoff function

As mentioned in the main document, local descriptors, such as symmetry functions are used in combination with cutoff functions to truncate the effective chemical environment of each atom. For the sake of simplicity, we have decided to employ a simple cosine-based function, as given by

$$f_c(r_{ij}) = \begin{cases} 0.5 \cdot \left[cos(\frac{\pi r_{ij}}{r_c}) + 1\right], r_{ij} \leq r_c \\ 0.0, r_{ij} > r_c, \end{cases} \tag{3}$$

where r_{ij} is the inter-atomic distance between particles i and j and r_c is the so-called cutoff radius, which controls the locality of the descriptor.

4.2 Optimization of the ideal number of GMM clusters

As previously mentioned, the Gaussian Mixture Models (GMM) decomposition requires the prior specification of the number of GMM components from which the observed sample population is claimed to arise. Different criteria have been derived to optimize the number of clusters, some of the most commonly employed ones, include:

- Elbow method: for each GMM model, a clustering score is computed and the optimum number of clusters is said to be that corresponding to the elbow in the score function (point above which convergence in the latter is achieved). In this way, the elbow correspond to the point above which increasing the number of GMM components brings little to no benefit in the actual clustering task.

- Silhouette analysis: in this approach, the fitting accuracy of each datapoint in the dataset, with respect to the assigned cluster probabilities, is computed, something which is often referred to as the Silhouette metric (s):

$$s = \frac{b - a}{max(a, b)}, \tag{4}$$

where a and b represent, respectively, the mean intra-cluster and intercluster distances. The latter is measured with respect to the immediately near clusters. The s score will peak at the point where the intercluster distance is maximized while minimizing the intra-cluster dispersion, leading to a prominent maximum in the evolution of the s metric. The Silhouette analysis is usually claimed to offer a more trustworthy evaluation of the ideal number of clusters in the sample problem.

- Information criteria metrics, such as the Akaike Information Criterion (AIC) or the Bayesian Information Criterion (BIC) estimate the balance between the quality of the clustering algorithm and its complexity: increasing the number of clusters of a model will generally improve the quality of the estimation at the expense of making the GMM model more prone to over-fitting. Thereby, BIC or AIC metrics penalize excessively complex models with poor inferring abilities. For instance, the general BIC kernel for a population of N datapoints takes the following expression

$$BIC = k \cdot ln(N) - 2 \cdot LL, \tag{5}$$

where k is the number of free model parameters and LL is the log-likelihood kernel. The former can be estimated from the dimensions of the data (d) and the number of GMM components (c),

$$k = (d \cdot c) + (c - 1) + c \cdot (d \cdot (d + 1)/2). \tag{6}$$

On the other hand, LL evaluates the correspondence between the predictions and observations

$$log(L) = \sum_{i=1,N}^{N} log\left[\sum_{j=1}^{c} w_j \cdot pdf(x_i, \mu_j, \sigma_j)\right], \tag{7}$$

where $pdf(x, \mu, \sigma)$ is the probability density function of a normal distribution with mean μ and standard deviation σ, evaluated at the point x. Altogether, this approach is specially suited for complex sample populations, where the starting data distribution may not necessarily arise from the combination of isolated and well-defined spherical components. Although lower BICs suggest better performances, the number of components is often chosen on the basis of the gradients instead: the evolution of the score gradients with the number of clusters will eventually reach a plateau and thus explicitly penalize too intricate GMM models.

4.3 Automatic selection of radial ACSF functions

The current section gathers the basic details about the algorithm used to select the radial Atom Centered Symmetry Functions (ACSF).

- First, the geometries are read from a trajectory file arising, for instance, from Molecular Dynamics simulations or normal mode analysis. The parameter `trj_step` controls the sampling interval in frames (`trj_step`= 1 will sample all geometries in the trajectory file).

```
geometries = read_xyz_trajectory_wstep(trjname,sampling_step=trj_step)
```

- The distance between all possible atomic pairs is computed and subsequently stored in a dictionary sorted in terms of the chemical composition of the pairs (e.g C-C, O-H). Additionally, a list of all unique element symbols found in the database is computed.

```
symbols,dist_dists=compute_distance_distribution(geometries)
```

- The code then loops over all possible elemental pairs. For each of them, the radial distribution is estimated from the previously computed pairwise distances stored in the dictionary.

```
for idx1 in np.arange(0,len(symbols)):   # i=1,N
    for idx2 in np.arange(idx1,len(symbols)): # j=i,N
        elemi=str(symbols[idx1])
        elemj=str(symbols[idx2])

        # Retrieve distances for elemi-elemj pairs
        dist=dist_dists[elemi][elemj]

        # Discard null values (appearing for same element distribution)
        mask = dist != 0
        dist=dist[mask]

        # Parse the distribution to 2D data:
        x,y=extract_distribution(dist, nbins)
        x= np.reshape(x, (-1, 1))
        y= np.reshape(y, (-1, 1))
        xydist=np.hstack((x, y))
```

- A cutoff function filter is then applied to the radial distribution to ensure that the GMM decomposition is only performed within the local chemical environments. This can be applied according to two slightly different approaches: a soft cutoff, relying on the cosine kernel shown in Eq. 3, and a hard cutoff (all values larger than the cutoff radius are discarded). It should be noticed that the latter is not generally recommended as it can introduce artifacts in the radial distribution which may hinder the GMM decomposition.

```
# Apply a cutoff function
xydist_cut=apply_cut(xydist,rcut,cut_type=cut_type)
```

The resultant local distribution can be smoothed (if desired) with the aid of a Kernel Density Estimation (KDE), something which can be beneficial when dealing with a reduced number of datapoints.

```
if (smooth == "yes"):
```

```
    xydist_cut_kde,samples=smooth_data(xydist_cut,bw)
```

- Starting from the previously filtered distribution, the optimum number of GMM clusters is estimated using one of the aforementioned criteria (e.g BIC). Afterwards, the parameters of the optimized GMM components are obtained.

```
# Optimize the number of GMM components
n_comp=find_opt_components(dist_dat,nmax,max_iter)

# Retrieve the hyperparameters of each of the GMM components
gmm,g_means,g_covariances,g_weights,sum_gau,grid=find_gmm_params(dist_dat,n_comp,max_iter,nbins)
```

If desired, each GMM component can be further decomposed in as many sub-clusters as indicated by the `ndecomp` variable.

```
# Print the radial functions and decompose them into their sub-components if desired
gmm_sub=print_gmm_components(gmm,decomp=ndecomp)
```

Doing so provides a way to increase the radial resolution in the most relevant regions of the space, affording thus a more accurate radial fingerprint of the system. The following figure shows an example of the change in radial resolution offered by this technique.

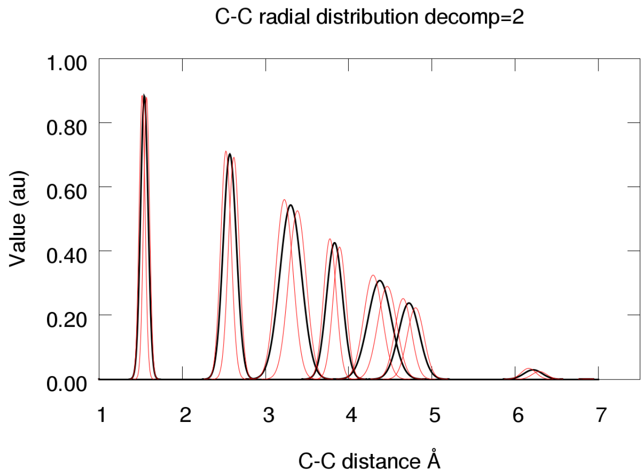

Figure 2: GMM components (in black), along with their sub-GMMs (in red), for the radial C-C distribution of peptide-capped alanine. Each GMM was decomposed in two additional normal distributions, increasing the radial resolution.

- Generally speaking, the pairwise distance distribution of a system will exhibit a collection of quasi-discrete clusters arising from the intrinsic chemical features of the system. Hence, the GMM decomposition will often yield unevenly distributed Gaussian functions which may not necessarily span along the whole range of distances from 0 to r_c. Although this should not be a problem on its own, it may be convenient to include auxiliary functions. The latter are fuzzy Gaussians tailor-made to fill the "blank gaps" of the observed GMM mixture. In order to account for the latter (if desired by the user), the overlap between consecutive clusters is estimated and, if lower than a given threshold `over_thres`, an auxiliary function is included between the two:

```
# Include auxiliary functions, if desired
if (aux == "yes"):
    aux_gaussians = compute_auxiliar_gaussians(gmm, (0,rcut),over_thres)
    for t in range(len(aux_gaussians)):
        val=aux_gaussians[t]
        mean=val[0]
```

```
    variance=val[1]**2
```

In the current version of the code, auxiliary functions are only included between the lower and upper bounds of the GMM model, thereby excluding the extremely short and long distance regimes. The following figure shows the effect of including a bunch of fuzzy on the overall distribution of radial cluster.

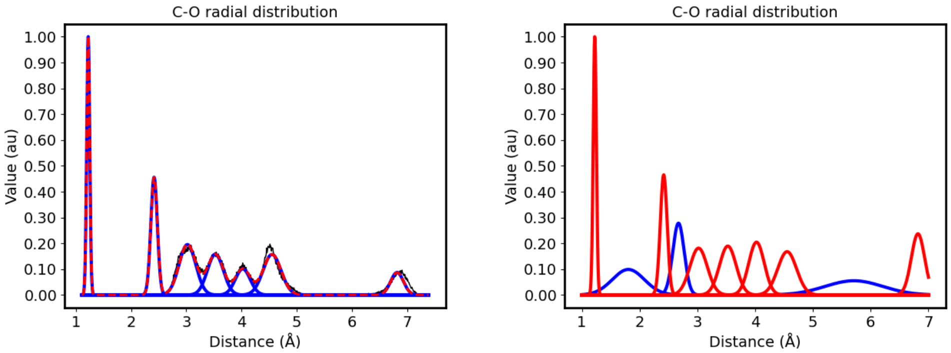

Figure 3: GMM decomposition of the radial environments of the C-O distribution (left) along with the auxiliary functions used to cover intermediate regimes, shown in blue (right).

- Finally, the selection of GMM components and sub-components along with their auxiliary functions are transformed into the radial ACSF parameters.

```
# Save the radial ACSF to a file:
outname=str(elemi)+str(elemj)+".rad"
rad_acsf=save_radial_acsf_params(gmm=gmm,gmm_sub=gmm_sub,aux=aux_gaussians,file=outname)
```

It's important to highlight that the transition from the Gaussian Mixture Model to the radial ACSF feature spaces is straightforward. This simplicity arises from the resemblance in the kernels of the underlying Gaussian functions. Let's start by considering the general expression of a standard two-body (radial symmetry) function as given by,

$$G_i^{rad} = \sum_{j \neq i}^{N} e^{-\eta (r_{ij} - r_s)^2} \cdot f_c(r_{ij}). \tag{8}$$

This expression is completely analogous to that of a uni-dimensional Gaussian distribution, $G(r)$,

$$G(r) = A \cdot e^{-\frac{1}{2 \cdot \sigma^2} \cdot (r-\mu)^2}, \tag{9}$$

where A is a scaling factor to ensure the adequate normalization of the function. Similarly, the parameters μ and σ define the center (mean) and width (standard deviation) of the distribution. It is thus evident that the intrinsic parameters of the radial ACSF kernel (η and r_s) can be readily obtained from the latter as,

$$\begin{cases} r_s = \mu \\ \\ \eta = \frac{1}{2\sigma^2} \end{cases}, \tag{10}$$

which allows us to exactly reconstruct two-body ACSF features from the previously found clusters in the radial distribution of a given atomic pair.

In our code, this mapping is internally handled by the save_radial_acsf_params function which receives the best-fitting set of GMM radial clusters, each one giving rise to a specific radial ACSF, as,

```
[IN] save_radial_acsf_params:
     gmm_mu    = gmm.means_.flatten()
     gmm.sigma2 = gmm.covariances_.flatten()
     gmm_eta   = 1/(2*gmm_sigma2)
```

Auxiliary functions along with the decomposed, displaced and binary GMMs are modified beforehand and are finally added to the overall radial set following this same procedure.

4.3.1 Evenly distributed symmetry functions

For the sake of comparison, the performance of the self-optimized radial symmetry functions can be compared to that offered by a collection of normal distributions designed to evenly sample the radial dimension. For such a purpose, a total of N gaussians are evenly distributed in the space (from 0 to r_c) and their standard deviations are set to achieve a homogeneous overlap between any two consecutive functions:

```python
def even_gaussians(rc, n):
    """
    A function to create n evenly sampled Gaussian functions in the space going
    from 0 to rc.
    """
    widths  = np.full(n, rc / n)              # Initialize width array
    overlaps = np.diff(np.linspace(0, rc, n + 1)) # Estimate the overlaps
    widths[:-1] *= np.sqrt(overlaps[1:] / overlaps[:-1]) # Adjust the width

    gaussian_mean = []
    gaussian_sdev = []

    for i in range(n):
        gaussian_mean.append(i * rc / n + widths[i] / 2) # Mean of the Gaussian
        gaussian_sdev.append(widths[i] / 2) # Standard deviation of the Gaussian

    return gaussian_mean,gaussian_sdev
```

It should be noticed that this approach will result in a collection of fuzzy radial functions lacking any specificity at describing any particular functional groups or discrete chemical moieties. We note in passing that, despite its success and simplicity, evenly sampled radial functions have their own inconveniences, particularly when it comes to selecting the density of the radial grids: the use of fine meshes will result in quite a few dummy gaussians (which will never get activated) which can have detrimental effects in the training of the models. Moreover, these dummy gaussians will exhibit constant null values throughout the training data, hampering the normalization of the input space, and are, thus, often removed during the treatment of the data prior to the model training.

4.4 Automatic selection of angular ACSF functions

The current section comprises the details of the code designed to optimize the angular symmetry functions allowing, thus, to include 3-body information in the local chemical descriptors.

- Just as in the case of the radial ACSF, the code starts by reading the molecular snapshots, which are stored in the `geometries` variable.

```
geometries = read_xyz_trajectory_wstep(trjname,sampling_step=trj_step)
```

- The radial and angular distributions are then computed along with the possible elemental composition found in the data.

```
symbols,chemdist=compute_angles(geometries)
```

The previous subroutine runs over each geometry in `geometries`, for each of the latter all possible atomic trios (centered at atom i and formed by neighboring atoms j and k) are computed. We note in passing that, for the sake of efficiency, the loop is executed, exclusively, for the unique atomic combinations ($i > j > k$).

```
for geometry in geometries: # Iterate over each geometry in the set
    positions = geometry['positions']
    symbols = geometry['symbols']
    for i in range(0,natoms-2):     # 1  to N-2
        for j in range(i+1,natoms-1): # i+1 to N-1
            for k in range(j+1,natoms): # j+1 to N
```

For each atomic trio, the angles and pairwise distances are obtained. Since the angle is computed with an `arccos` function, all the values will span over a range from 0 to 180 °, corresponding thus to the inner angle centered at the reference atom, i,

```
atom_i = positions[i]
atom_j = positions[j]
atom_k = positions[k]
# Compute distances from central atom (i) to neighboring atoms (j,k)
dist_i_j = np.linalg.norm(atom_i - atom_j)
dist_i_k = np.linalg.norm(atom_i - atom_k)
# Compute the angle centered at i
v1 = atom_j - atom_i
v2 = atom_k - atom_i
angle = np.arccos(np.dot(v1, v2) / (np.linalg.norm(v1) * np.linalg.norm(v2)))
angle = np.degrees(angle)
```

It should be noticed that in order to achieve an unbiased picture of the chemical features of the system, the three equivalent angular descriptions of every atomic trio are taken into account. More information on this is provided in Section 4.4.1. The θ_i^{jk}, r_{ij} and r_{ik} values are finally stored in the output dictionary accounting for the permutational invariance of the ij and ik pairs

```
if symbols[j] == pair[0]:
    chemdist[symbols[i]][pair].append((angle, dist_i_j, dist_i_k))
else:
    chemdist[symbols[i]][pair].append((angle, dist_i_k, dist_i_j))
```

where the `pair` label is formed form concatenation of the j and k (sorted) atomic symbols.

- The code then iterates over all possible atomic trios arising from the combination of the element and pair types. Since not all trios may be present in the system (e.g if only 2N atoms are present, there will be no N-NN trios), the parse is only executed for `len(data) > 0`.

```
for elem, pairs in chemdist.items():
    for pair, data in pairs.items():
```

```
      if len(data) > 0:
```

- For each of the latter combinations, the data are truncated up to the cutoff radius, as previously explained for the radial symmetry functions.

```
data=filter3Ddata(data,rcut)
```

In this case, however, a hard cutoff scheme is applied for the sake of simplicity: those (θ_i^{jk}, r_{ij},r_{ik}) points for which the r_{ij} or r_{ik} distances are larger than the cutoff radius are discarded with the aid of a mask (logic array)

```
mask = (dist_ij <= rcut) & (dist_ik <= rcut)
angles_filtered = angles[mask]
dist_ij_filtered = dist_ij[mask]
dist_ik_filtered = dist_ik[mask]
data = np.column_stack((angles_filtered,dist_ij_filtered,dist_ik_filtered))
```

- Given the considerably large intricacy that can be found in the angular space, the data are normalized to aid the GMM decomposition

```
data_norm,data_mean,data_std=norm_3ddata(data)
```

- The ideal number of GMM components is optimized just as in the case of the radial symmetry functions

```
n_comp=find_opt_components3D(data_norm,nmax=nmax,max_iter=max_iter,cv_type=cv_type,
      crit=gmm_crit,percbic=percbic,percdiff=percdiff,file=outname)
```

Owing to the substantial increase in complexity when moving from a single-dimension mixture model to a multi-dimensional space, more sophisticated approaches should be used to prevent the selection of an excessively large number of GMM components. The `gmm_crit` variable controls whether the latter is determined based on the minimum BIC score or relying on its convergence. In the case of the latter, the `percbic` and `percdiff` parameters set the thresholds for the BIC score and its gradient. In this way, the minimum number of clusters for which the BIC score, and its gradient, are within these thresholds will be selected. This approach will generally penalize an excessively large number of clusters, as exemplified in the left panel of Fig. 8, which can result in too intricate and poorly performing GMM models.

It should be noticed that the percbic, percdiff, nmax and afrac parameters must be selected with care as they can dramatically change the suitability of the resultant angular ACSF functions.

```
# Compute the BIC score and BIC gradient percentiles
percentiles = np.arange(0, 101)
diff_distribution = [np.percentile(abs(diff), p) for p in percentiles]
bic_distribution = [np.percentile(bic_scores, p) for p in percentiles]

# Set convergence threshold as a percentage of the maximum change in smoothed BIC scores
bic_thres = bic_distribution[percbic]
diff_thres = diff_distribution[percdiff]

# Initialize the number of components
best_n_components = n_components_range[np.argmin(bic_scores)]
for p in range(1,nmax-1):
   low_dev=abs(bic_scores[p]-bic_scores[p-1])
   up_dev=abs(bic_scores[p+1]-bic_scores[p])
   score=bic_scores[p]
   if (low_dev <= diff_thres and up_dev <= diff_thres and score <= bic_thres) == True:
      best_n_components=p
      break
```

The parameters of the GMM model for the optimum number of components are then retrieved

```
gmm,g_means,g_covariances,g_weights=find_gmm_params3D(data_norm,n_comp,max_iter,cv_type=cv_type)
```

The following figures show an example of the observed and reconstructed angular and radial distributions arising from the code, evidencing its success at capturing the chemical features of the data.

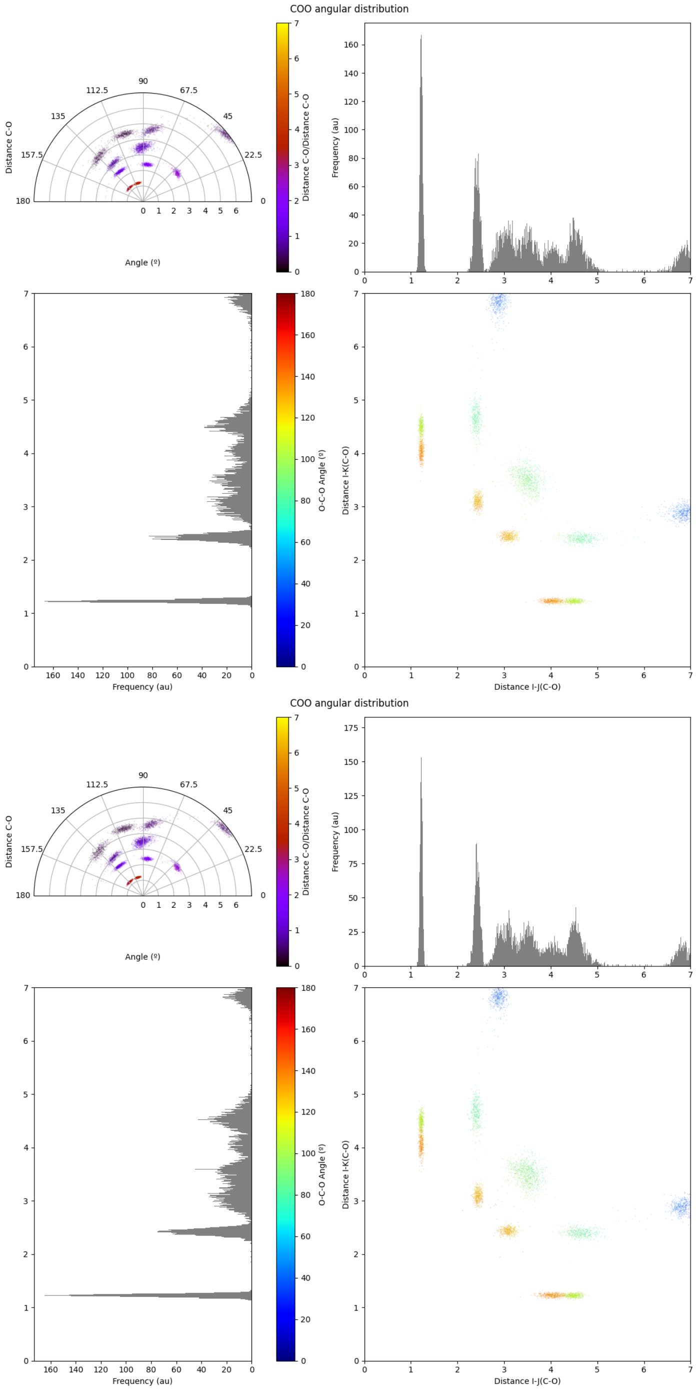

Figure 4: Observed (top) and GMM reconstructed (bottom) angular distribution of the C-OO atomic trio for peptide-capped alanine. The radial dispersion plot is colored according to the θ_i^{jk} values, whereas the polar plot is colored according to the ratio r_{ik}/r_{ij} ratio to show the heterogeneity in the neighboring pairwise distances.

- Finally, the GMM parameters are transformed into the ACSF feature space so as to determine the optimum hyperparameters of the angular symmetry functions. Although, as explained in the main manuscript, different functional forms have been derived for the latter, the heavily modified angular ACSF [8] kernel, shown in Eq. 11, will be used owing to its large flexibility and performance.

$$G_i^{ang} = 2^{1-\xi} \sum_{j,k\neq i}^{N} (1+\cos(\theta_{ijk}-\theta_s))^{\xi} \cdot exp\left[-\eta\left(\frac{r_{ij}+r_{ik}}{2}-r_s\right)^2\right] \cdot f_c(r_{ij}) \cdot f_c(r_{ik}), \tag{11}$$

Unfortunately, unlike the radial ACSF functions where the parameters can be easily and precisely reconstructed from the GMMs, the situation is significantly more challenging for angular features. This difficulty arises from the slightly different kernels displayed by the heavily modified angular ACSFs with respect to that of a regular Gaussian distribution. In fact, this becomes evident if we compare Eq. 11 with the kernel of a Gaussian distribution in the three-dimensional space, $G(\theta, r_{ij}, r_{ik})$, as,

$$G(r_{ij}, r_{ik}, \theta) = A \cdot \exp\left(-\frac{1}{2}\left(\frac{(r_{ij}-\mu_{r_{ij}})^2}{\sigma_{r_{ij}}^2} + \frac{(r_{ik}-\mu_{r_{ik}})^2}{\sigma_{r_{ik}}^2} + \frac{(\theta-\mu_\theta)^2}{\sigma_\theta^2}\right)\right), \tag{12}$$

where A represents the normalization factor and with μ and σ being the mean and standard deviation, respectively, of the distribution in each of the dimensions of the space, namely the pairwise distances to the nearest neighbors $\{r_{ij}, r_{ik}\}$ and the angle formed by each atomic trio, θ.

Since both kernels must be centered about the same values, the θ_s shift can be set equal to the mean of the GMM component in the angular space,

$$\theta_s = \mu_\theta. \tag{13}$$

This is directly handled by the built-in `save_angular_acsf_params` function,

```
[IN] save_angular_acsf_params
angular_acsf=[]
# The main parameters of the GMM components in the 3D (angle, rij, rik) space are first retrieved
for i in range(N):
    mu_angle.append(gmm_mu[i][0])
    mu_rij.append(gmm_mu[i][1])
    mu_rik.append(gmm_mu[i][2])
    sigma_angle.append(np.sqrt(gmm_cov[i,0,0]))
    sigma_rij.append(np.sqrt(gmm_cov[i,1,1]))
    sigma_rik.append(np.sqrt(gmm_cov[i,2,2]))
# Then, the GMM parameters are mapped to the angular ACSF feature space.
# Map the centroid of the angular distribution
theta_s=[]
for i in range(N):
    theta_s.append(mu_angle[i])
```

Applying a similar rationale, the center of the radial distribution for the angular ACSF function can be directly derived as the mean value of the centroid of the Gaussian in the pairwise distance dimensions to the nearest neighbors.

$$r_s = \frac{\mu_{r_{ij}} + \mu_{r_{ik}}}{2}, \tag{14}$$

which is, again, handled by the `save_angular_acsf_params` function:

```
[IN] save_angular_acsf_params
# Map the centroid of the radial distribution
rs=[]
for i in range(N):
    rs.append((mu_rij[i]+mu_rik[i])/2)
```

Likewise, and just as in the case of the radial ACSF kernel, the width of the radial distribution, as controlled by the η parameter, can be once again obtained from the standard deviation of the GMM components in the radial dimensions, as already detailed in Eq. 10,

$$\eta = \frac{1}{2\sigma^2}. \tag{15}$$

Notice that since the heavily modified angular ACSF kernel uses a single radial grid for both nearest neighbors, the η parameter is derived from the mean values of the r_{ij} and r_{ik} radial distributions of the three-dimensional GMM, that is,

$$\eta = \frac{1}{2\overline{\sigma}^2}, \tag{16}$$

with,

$$\overline{\sigma} = \frac{\sigma_{rij} + \sigma_{rik}}{2}, \tag{17}$$

which is implemented as:

```
[IN] save_angular_acsf_params
# Map the width of the radial distribution
eta=[]
for i in range(N):
    # Get the eta parameters: (obtained as the average value)
    sigma2dum=((sigma_rij[i] + sigma_rik[i])/2)**2
    eta.append(1/(2*sigma2dum))
```

Unfortunately, finding the last unknown variable of the angular ACSF kernel, namely the ξ parameter, is considerably more difficult as the equality between the Gaussian distribution and the heavily modified angular ACSF kernel seems to lack a proper analytical solution. To overcome this inconvenience, we have opted to determine the value of ξ by means of a numerical optimization approach to ensure that the cosine kernel accurately reproduces the space covered by the Gaussian distribution arising from a given component of the GMM model. Comparing the angular cosine kernel of Eq. 11 with that used in the exponential core of a Gaussian function yields,

$$\left\{ \begin{array}{l} 2^{1-\xi} \cdot (1 + \cos(\theta_{ijk} - \theta_s))^{\xi} \\ A \cdot e^{-\frac{(\theta-\mu_\theta)^2}{2\sigma_\theta^2}} \end{array} \right\}.$$

which allows us to map the features of the latter to those of the target angular function. To do so, the internal function `anglegaus_2_anglecos` is used. The latter starts by defining the target cosine function of the form,

$$((1 + \cos(x - x_s))^w \times 2^{1-w}), \tag{18}$$

which is a one-dimensional function whose shape is tuned with the aid of the x_s and w parameters. Similarly, a one-dimensional gaussian function is also defined,

$$A \cdot \exp\left(-\frac{(x-\mu)^2}{2\sigma^2}\right), \tag{19}$$

where, for the sake of convenience, the A scaling factor is set to 2 so as to ensure that both kernels span over similar ranges of values. Then a numerical optimization routine, as built-in within the SciPy Python module,[9] is used to find the ξ parameter, while the angular shift is kept fixed to the centroid of the GMM cluster in its angular domain, $\theta_s = mu$,

```
# We will adjust between gaussians between 10 sigmas
x = np.linspace(mu - 10 * sigma, mu + 10 * sigma, 10000)
# Generate y values using the cosine function with Gaussian noise
y = gaus_kernel(x, A, mu, sigma)
# Set the center of the function to mu
xs = mu
# Fit the data to a Gaussian function
popt, _ = curve_fit(cos_kernel, x, y)
# Extract the parameters of the Gaussian function
w_fit = popt
```

To ease the optimization procedure, while trying to fit as much as possible the cosine kernel to the underlying exponential function of the GMM, the space is restricted to $\pm 10\sigma$, centered at μ.

Being a numerical optimization routine, convergence may not be guaranteed, thus the ratio between the areas covered by the Gaussian and cosine kernels is used as a rough metric of their overlap.

```
# Compute the overlap between the gaussian and cosine kernels
# Calculate the values of both functions
y_cos = cos_kernel(x, w_fit)
y_gaus = gaus_kernel(x, A, mu, sigma)

# Calculate the areas under both curves
area_cos = simps(y_cos, x)
area_gaus = simps(y_gaus, x)

# Calculate the overlap ratio
overlap_ratio = area_gaus / area_cos
```

Overlap values significantly differing from 1.0 indicate sub-optimal ξ parameters.

4.4.1 Dealing with the angular redundancies

This section details the procedure used to handle the potential redundancies arising from the origin-dependent angular descriptions of a three-atom combination.

For the sake of simplicity, let's consider an atomic trio of C, H and O atoms. Although such a configuration is given by a unique set of three distances (r_{CH}, r_{CO}, r_{OH}) there are different, yet equivalent, definitions owing to the reference-dependence of the inner-angles. If these redundancies were not taken into account, running the parse for $i \neq j \neq k$, the number of possible combinations would be given by $N \cdot (N-1) \cdot (N-2)$, with N being the number of atoms in the system. On the other hand, accounting for the permutational symmetry of the neighboring j and k atoms (e.g a CH or HC neighbor pairs are indistinguishable) by introducing the $i > j$ constraint reduces the number of possible configurations to $\frac{N \cdot (N-1) \cdot (N-2)}{2!}$. Finally, taking into account only, exclusively, the unique configurations of the three-body combination, $i > j > k$, results in a much more reduced number of configurations ($\frac{N \cdot (N-1) \cdot (N-2)}{3!}$) since all the redundancies are removed.

```
for i in range(0,natoms-2):
    for j in range(i+1,natoms-1):
            for k in range(j+1,natoms):
                    TPset=[]
                    TPset.append(i)
                    TPset.append(j)
                    TPset.append(k)
                    # Account for the 3 possible descriptions of the triangle
                    for p1 in TPset:
                        atom_cen=positions[p1]
                        nset=TPset.copy()
                        nset.remove(p1)
                        p2=nset[0]
                        p3=nset[1]
                        atom_nei1=positions[p2]
                        atom_nei2=positions[p3]
                        # Compute the distances
                        dist_cen_nei1=np.linalg.norm(atom_cen - atom_nei1)
                        dist_cen_nei2=np.linalg.norm(atom_cen - atom_nei2)
                        # Compute the angle centered at p1
                        v1 = atom_nei1 - atom_cen
                        v2 = atom_nei2 - atom_cen
                        angle = np.arccos(np.dot(v1, v2) / (np.linalg.norm(v1) * np.linalg.norm(v2)))
                        angle = np.degrees(angle)
```

```
pair = symbols[p2] + symbols[p3] if symbols[p2] <= symbols[p3] else symbols[p3] +
    symbols[p2]
# Check the id of the atomic pairs
if symbols[p1] not in chemset:
    chemset[symbols[p1]] = {}
    symbols_set.append(symbols[p1])
if pair not in chemset[symbols[p1]]:
    chemset[symbols[p1]][pair] = []
# Append the results, accounting for redundancies in the case of an homoatomic pair
if symbols[p2] == pair[0]:
    chemset[symbols[p1]][pair].append((angle, dist_cen_nei1, dist_cen_nei2))
if symbols[p3] == pair[0]:
    chemset[symbols[p1]][pair].append((angle, dist_cen_nei2, dist_cen_nei1))
```

Although the code only iterates over the unique atomic trios, it accounts for the three possible descriptions of each of them, key to get an unbiased angular distribution regardless on the position of the reference atom. For instance, in the case of an homoatomic pair (e.g CHH, NOO or HHH), the data are appended twice (with the two possible descriptions of the neighboring configuration) in order to account for the symmetries of the system.

For the sake of computational time, only the upper triangular part of the distribution will be parsed to the GMM clustering algorithm, something which is handled with the aid of the following mask.

```
# In case the pair is formed by the same elements, we need to account for symmetry
if (pair[0] == pair[1]):
   mask_sym = (dist_ik_filtered >= dist_ij_filtered)
   angles_filtered = angles_filtered[mask_sym]
   dist_ij_filtered = dist_ij_filtered[mask_sym]
   dist_ik_filtered = dist_ik_filtered[mask_sym]
```

It is worth mentioning that doing so will introduce minor artifacts in the shape of the clusters centered at the diagonal (with very similar or identical r_{ij} and r_{ik} mean values). However, this does not usually result in a noticeable worsening of the clustering accuracy.

The following figures show the effect of running the GMM clustering for all the combinations (including redundancies) or for the data corresponding, solely, to the upper triangular distribution for different scenarios (atomic configurations).

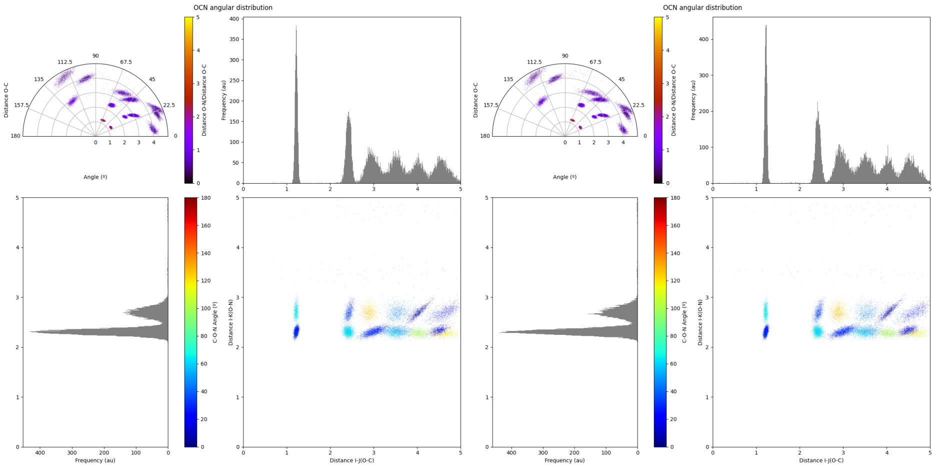

Figure 5: GMM clusters for an O-C-N distribution computed with the entire (left) and upper-triangular (right) distributions.

Whereas both approaches yield the same results for the hetero-atomic case, accounting for the redundancies affords only the upper half of the distribution in the homo-atomic scenario. Given that the latter is symmetric, the same features of the local chemical environments can be recovered at a much lower computational cost. Furthermore, it is worth mentioning that, as expected, the number of GMM clusters is decreased to half the starting value which results in significant speedups and better performances of the clustering technique.

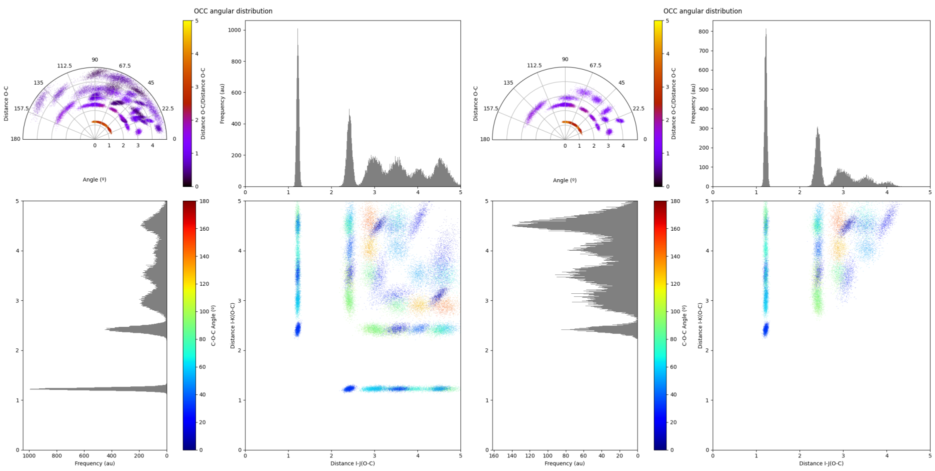

Figure 6: GMM clusters for an O-C-C distribution computed with the entire (left) and upper-triangular (right) distributions.

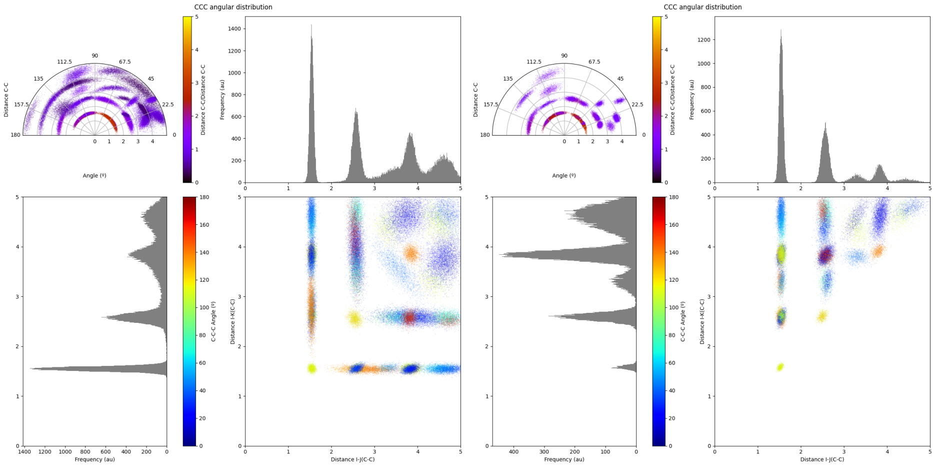

Figure 7: GMM clusters for an C-C-C distribution computed with the entire (left) and upper-triangular (right) distributions.

This becomes even more evident from the evolution of the score metric with the number of GMM clusters using both datasets, as gathered in the following figures.

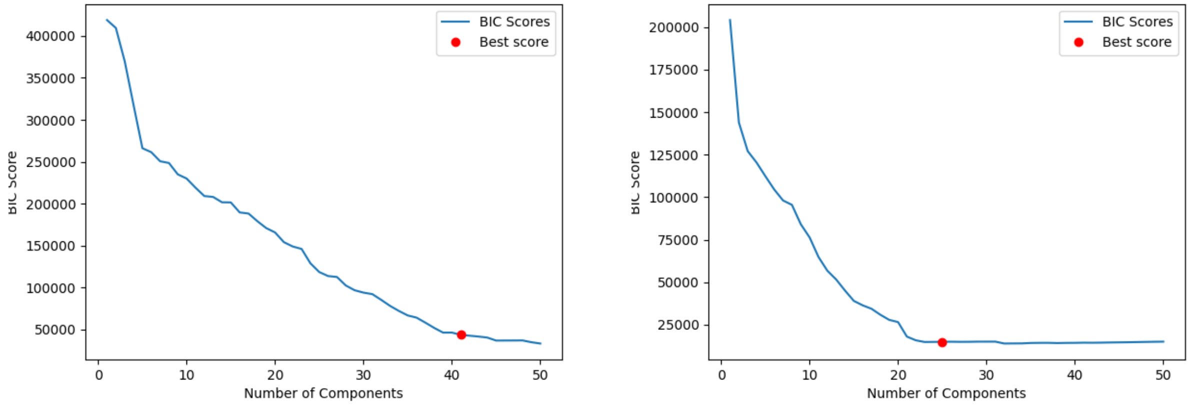

Figure 8: Evolution of the BIC scores as a function of the number of GMM clusters for the O-C-C distribution, computed with the entire (left) and upper-triangular (right) distributions.

4.5 Resampling techniques

This section comprises a brief summary of the different resampling techniques designed to increase the accuracy of the Atomic Environment Vectors (AEVs) by perturbing the original spatial distribution of the symmetry functions.

Decomposed gaussians

In this approach, each of the clusters resulting from the initial GMM is expressed as a combination of K additional gaussian functions, referred here to as GMM sub-components. The following figure shows the effect of increasing the number of sub-decompositions in the description of the C-C radial environment.

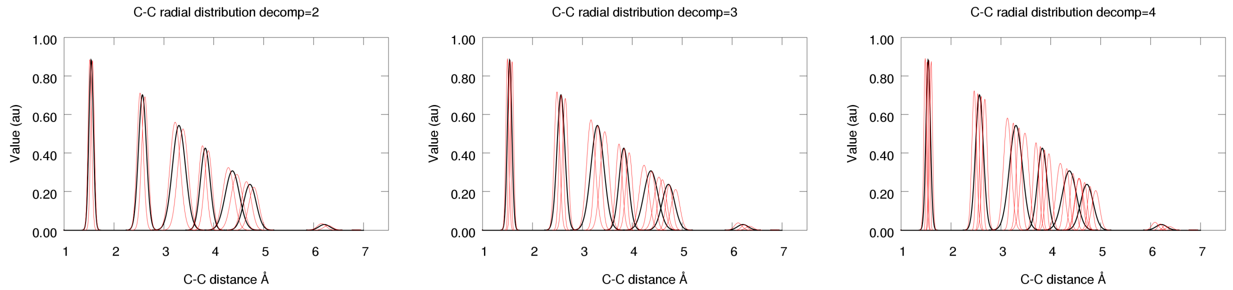

Figure 9: C-C radial GMM components with an increasing number of sub-decompositions (shown in red) starting from a collection of tailor-made functions (shown in black).

Displaced gaussians

As previously mentioned, in this approach the center and width of the tailor-made gaussian (that built to exactly reproduce one of the GMM components) are modified so that the lower tail of the Gaussian spans over both tails of the cluster. The α parameter is used to control the offset of the function in terms of σ, as :

$$\left\{ \begin{array}{l} \mu^* = \mu + \alpha \cdot \sigma \\ \sigma^* = 2 \cdot \sigma \end{array} \right\}$$

By doing so, the lower tail of the new gaussian ($\mu^* - \alpha \cdot \sigma^*$,$\mu^*$) covers the whole range of the original cluster ($\mu - \alpha \cdot \sigma$, $\mu + \alpha \cdot \sigma$).

Binary gaussians

This approach employs a combination of two gaussians to describe the lower and upper tails of the main radial cluster, something which should provide higher resolution in the codification of the chemical environments. Following an analogous strategy to that employed in the case of the displaced gaussians, the mean and standard deviation of each gaussian are given, in terms of the center μ and standard deviation σ of a given cluster in the radial distribution, by the following expressions:

$$\left\{ \begin{array}{l} \mu^- = \mu - \alpha \cdot \sigma \\ \sigma^- = \sigma \cdot (1 + \frac{\beta}{\alpha}) \end{array} \right\}$$

$$\left\{ \begin{array}{l} \mu^+ = \mu + \alpha \cdot \sigma \\ \sigma^+ = \sigma \cdot (1 + \frac{\beta}{\alpha}) \end{array} \right\}$$

As can be seen from the previous expressions, the α parameter controls the offset of the center of the lower and upper functions, just as in the case of their displaced analogs. The following figure shows the effect of increasing the values of α while keeping a constant value of $\beta = 0$.

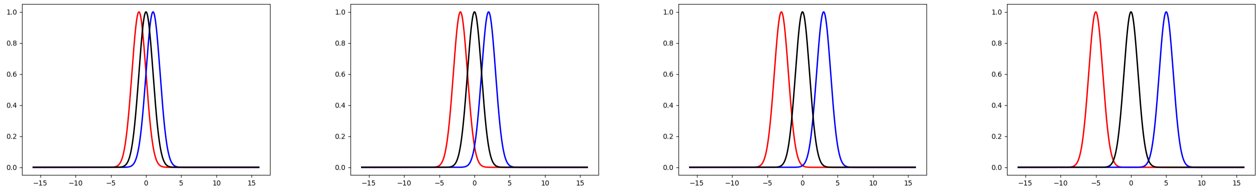

Figure 10: Effect of increasing the α values in the topology of the binary gaussians. A fixed value of $\beta = 0$ was employed in combination with an increasing $\alpha = 1, 2, 3$ and 5.

On the other hand, the β parameter controls the overlap of the $(\mu^- + \alpha \cdot \sigma^-)$ and $(\mu^+ - \alpha \cdot \sigma^+)$ tails of the lower and upper binary gaussians, respectively, such that:

$$\left\{ \begin{array}{l} \mu^- + \alpha \cdot \sigma^- = \mu + \beta \cdot \sigma \\ \mu^+ - \alpha \cdot \sigma^+ = \mu - \beta \cdot \sigma \end{array} \right\}$$

In this way, as the value of β is increased, the tails (defined by the $\alpha \cdot \sigma$ threshold) overlap more and more, arising from the interpenetration of both lower and upper gaussians. The effect of increasing the β values while keeping a constant value of α can be seen in the following figures:

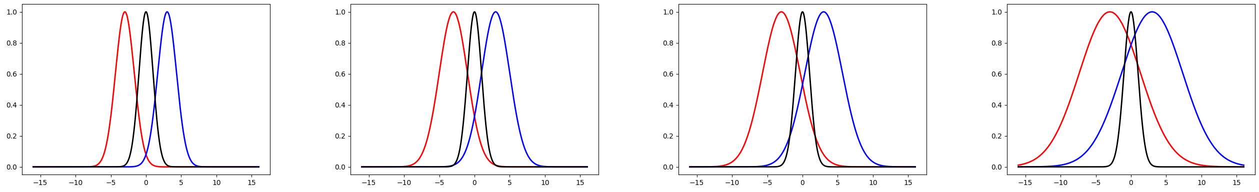

Figure 11: Effect of increasing the β values in the topology of the binary gaussians. A fixed value of $\alpha = 3$ was employed in combination with an increasing $\beta = 1, 3, 5$ and 10.

5 Optimized Radial Symmetry Functions

The current section comprises a summary of the optimization procedure of the radial symmetry functions, according to different schemes.

5.1 GMM clustering of the radial environments

In the upcoming figures, the radial distributions of the different atomic pairs of peptide-capped alanine will be shown along with the corresponding optimized clusters. For the optimization, a hard cutoff scheme ($r_c = 7.00$ Å) was employed. Additionally, the minimum BIC score, with a maximum number of 15 components, was used as a criterion to find the number of GMM components.

The following figure comprises the radial distribution, along with the GMM optimized clusters, of the C, H, O and N radial environments of the C atoms of peptide-capped alanine.

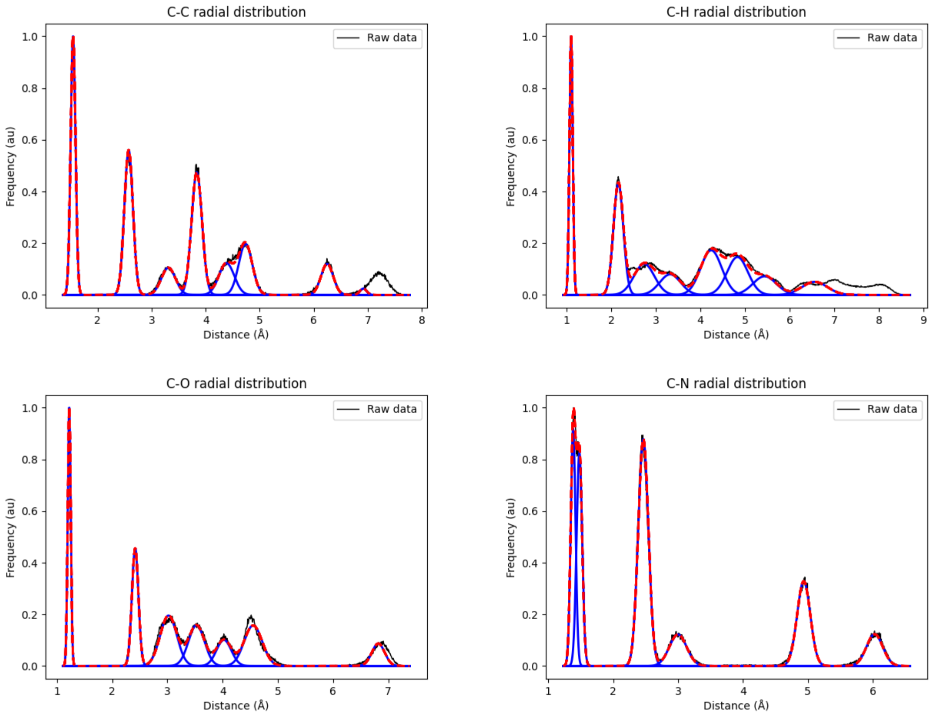

Figure 12: GMM decomposition of the radial environments of the C atoms of peptide-capped alanine. Individual clusters are shown in blue whereas their cumulative distribution is shown in red.

The following figure comprises the radial distribution, along with the GMM optimized clusters, of the C, H, O and N radial environments of the H atoms of peptide-capped alanine.

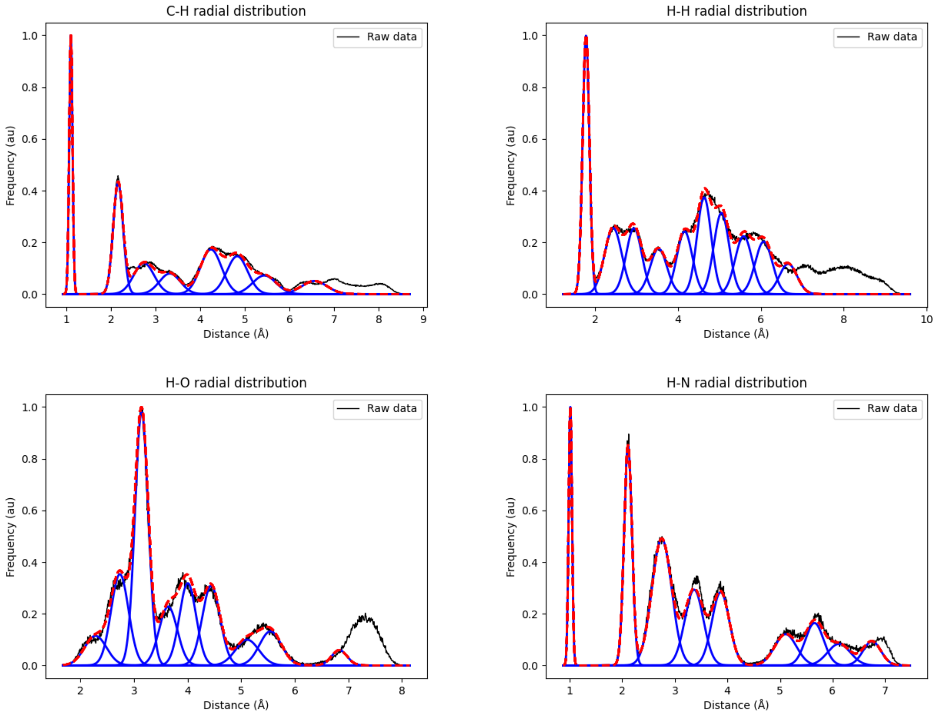

Figure 13: GMM decomposition of the radial environments of the H atoms of peptide-capped alanine. Individual clusters are shown in blue whereas their cumulative distribution is shown in red.

The following figure comprises the radial distribution, along with the GMM optimized clusters, of the C, H, O and N radial environments of the O atoms of peptide-capped alanine.

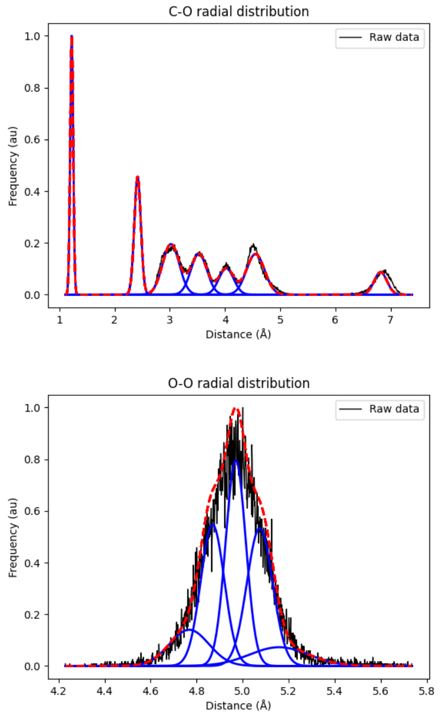

Figure 14: GMM decomposition of the radial environments of the O atoms of peptide-capped alanine. Individual clusters are shown in blue whereas their cumulative distribution is shown in red.

The following figure comprises the radial distribution, along with the GMM optimized clusters, of the C, H, O and N radial environments of the N atoms of peptide-capped alanine.

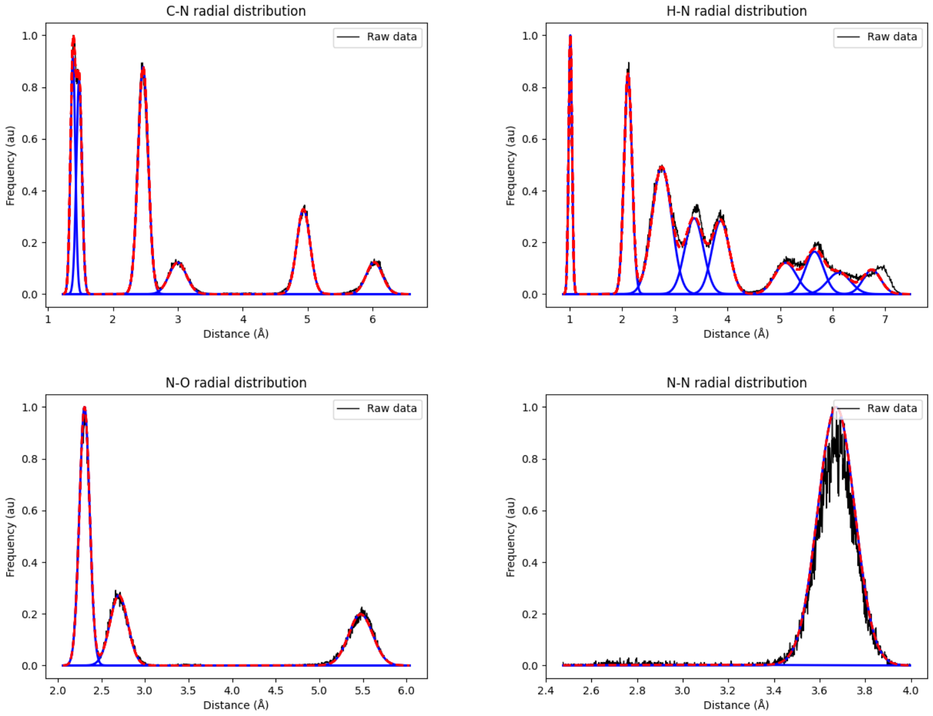

Figure 15: GMM decomposition of the radial environments of the N atoms of peptide-capped alanine. Individual clusters are shown in blue whereas their cumulative distribution is shown in red.

5.2 Decomposed functions

The following table comprises the performance of the models, reported in electrons, trained to predict the atomic charges of peptide-capped alanine using the tailor-made radial symmetry functions with a varying number of cluster decompositions. No auxiliary functions were included.

Table 1: Training (tr) and testing (ts) error metrics in the prediction of the atomic charges of peptide-capped alanine as a function of the number of decompositions (K) of the radial GMM components. No auxiliary functions were employed. N_{feat} indicates the total number of features employed in each case. All values are reported in electrons.

K= 0	MAE_{tr}	$RMSE_{tr}$	MAE_{ts}	$RMSE_{ts}$	N_{feat}
C	0.031	0.041	0.032	0.042	29
H	0.012	0.016	0.013	0.016	36
O	0.023	0.030	0.023	0.031	24
N	0.029	0.041	0.029	0.042	20
K= 2	MAE_{tr}	$RMSE_{tr}$	MAE_{ts}	$RMSE_{ts}$	N_{feat}
C	0.016	0.023	0.016	0.024	58
H	0.008	0.010	0.008	0.010	72
O	0.007	0.010	0.007	0.011	48
N	0.011	0.016	0.012	0.017	40
K= 3	MAE_{tr}	$RMSE_{tr}$	MAE_{ts}	$RMSE_{ts}$	N_{feat}
C	0.013	0.020	0.014	0.021	87
H	0.007	0.010	0.007	0.010	108
O	0.006	0.009	0.007	0.010	72
N	0.011	0.015	0.011	0.016	60
K= 4	MAE_{tr}	$RMSE_{tr}$	MAE_{ts}	$RMSE_{ts}$	N_{feat}
C	0.012	0.018	0.012	0.019	116
H	0.007	0.009	0.007	0.009	144
O	0.006	0.009	0.006	0.009	96
N	0.010	0.014	0.011	0.015	80

The following table comprises the performance of the models, reported in electrons, trained to predict the atomic charges of peptide-capped alanine using the tailor-made radial symmetry functions with a varying number of cluster decompositions. Auxiliar functions were included to prevent under-sampled regions of the potential energy landscape.

Table 2: Training (tr) and testing (ts) error metrics in the prediction of the atomic charges of peptide-capped alanine as a function of the number of decompositions of the radial GMM components. Auxiliary functions were employed. N_{feat} indicates the total number of features employed in each case. All values are reported in electrons.

0	MAE_{tr}	$RMSE_{tr}$	MAE_{ts}	$RMSE_{ts}$	N_{feat}
C	0.011	0.015	0.011	0.015	45
H	0.008	0.011	0.008	0.011	44
O	0.008	0.011	0.008	0.012	30
N	0.021	0.028	0.021	0.028	29
2	MAE_{tr}	$RMSE_{tr}$	MAE_{ts}	$RMSE_{ts}$	N_{feat}
C	0.009	0.014	0.009	0.013	74
H	0.007	0.009	0.007	0.009	80
O	0.006	0.009	0.007	0.009	54
N	0.011	0.015	0.011	0.015	49
3	MAE_{tr}	$RMSE_{tr}$	MAE_{ts}	$RMSE_{ts}$	N_{feat}
C	0.009	0.012	0.009	0.012	103
H	0.007	0.009	0.007	0.009	116
O	0.006	0.008	0.006	0.008	78
N	0.010	0.014	0.010	0.014	69
4	MAE_{tr}	$RMSE_{tr}$	MAE_{ts}	$RMSE_{ts}$	N_{feat}
C	0.009	0.013	0.009	0.012	132
H	0.007	0.009	0.007	0.009	152
O	0.006	0.008	0.006	0.008	102
N	0.010	0.014	0.010	0.014	89

The following figure shows the evolution of the testing error metrics in the prediction of the atomic charges of peptide-capped alanine with the number of decompositions employed.

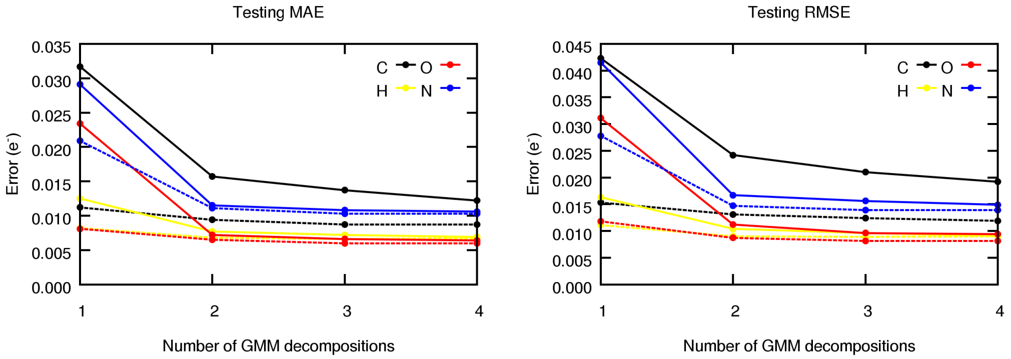

Figure 16: Evolution of the testing MAE and RMSE error metrics in the prediction of the atomic charges of peptide-capped alanine with the number of decompositions of each GMM component. Dashed lines are used to show the effect of including auxiliary functions.

5.3 Evenly sampled functions

The following table comprises a comparison of the testing MAE and RMSE error metrics in the prediction of the atomic charges of peptide-capped alanine using tailor-made radial functions or the same number of components evenly distributed in the radial space.

Table 3: Testing error metrics in the prediction of the atomic charges of peptide-capped alanine as a function of the number of radial ACSF employed. Left and right show the results for tailor-made (without auxiliary functions) and evenly distributed Gaussians, respectively. For the latter, the same number of functions as that dictated by the former was used. All values are reported in electrons.

Atom	N_{feat}	MAE_{tr}	$RMSE_{tr}$	MAE_{tr}	$RMSE_{tr}$
C	29	0.032	0.042	0.008	0.011
C	58	0.016	0.024	0.008	0.010
C	87	0.014	0.021	0.008	0.012
C	116	0.012	0.019	0.008	0.014
H	36	0.013	0.016	0.006	0.008
H	72	0.008	0.010	0.006	0.008
H	108	0.007	0.010	0.006	0.008
H	144	0.007	0.009	0.006	0.008
O	24	0.023	0.031	0.006	0.008
O	48	0.007	0.011	0.006	0.008
O	72	0.007	0.010	0.006	0.008
O	96	0.006	0.009	0.006	0.008
N	20	0.029	0.042	0.014	0.018
N	40	0.012	0.017	0.009	0.012
N	60	0.011	0.016	0.009	0.012
N	80	0.011	0.015	0.009	0.012

5.4 Displaced functions

The following table comprises the performance of the models, reported in electrons, trained to predict the atomic charges of peptide-capped alanine as a function of the offset introduced in the displaced gaussian functions. Furthermore, the corresponding results for the evenly distributed (even) and tailor-made (tm) gaussians are also shown.

Table 4: Training (tr) and testing (ts) error metrics for the FFNN models trained to predict the atomic charges of peptide-capped alanine as a function of the offset applied to the gaussian functions (α). The results for the tailor-made (tm) and evenly distributed gaussians (even), using the same number of features, are also shown. All values are given in electrons.

Atom	MAE_{tr}	$RMSE_{tr}$	MAE_{ts}	$RMSE_{ts}$
C (tm)	0.031	0.041	0.032	0.042
C (even)	0.008	0.011	0.008	0.011
C $\alpha = 1$	0.016	0.024	0.016	0.024
C $\alpha = 2$	0.010	0.014	0.010	0.014
C $\alpha = 3$	0.011	0.014	0.011	0.015
C $\alpha = 4$	0.012	0.017	0.012	0.018
C $\alpha = 5$	0.014	0.020	0.014	0.020
H (tm)	0.012	0.016	0.013	0.016
H (even)	0.006	0.008	0.006	0.008
H $\alpha = 1$	0.008	0.010	0.008	0.010
H $\alpha = 2$	0.007	0.010	0.007	0.010
H $\alpha = 3$	0.007	0.009	0.007	0.009
H $\alpha = 4$	0.008	0.010	0.008	0.010
H $\alpha = 5$	0.009	0.012	0.009	0.012
O (tm)	0.023	0.030	0.023	0.031
O (even)	0.006	0.008	0.006	0.008
O $\alpha = 1$	0.012	0.019	0.013	0.020
O $\alpha = 2$	0.007	0.010	0.007	0.010
O $\alpha = 3$	0.006	0.008	0.006	0.008
O $\alpha = 4$	0.006	0.008	0.006	0.008
O $\alpha = 5$	0.007	0.009	0.007	0.009
N (tm)	0.029	0.041	0.029	0.042
N (even)	0.013	0.018	0.014	0.018
N $\alpha = 1$	0.013	0.019	0.013	0.019
N $\alpha = 2$	0.010	0.014	0.010	0.014
N $\alpha = 3$	0.010	0.013	0.010	0.013
N $\alpha = 4$	0.011	0.015	0.011	0.015
N $\alpha = 5$	0.016	0.021	0.016	0.021

Similarly, the following figure comprises the evolution of the testing MAE and RMSE error metrics of the FFNN models trained on displaced radial symmetry functions as a function of the α value.

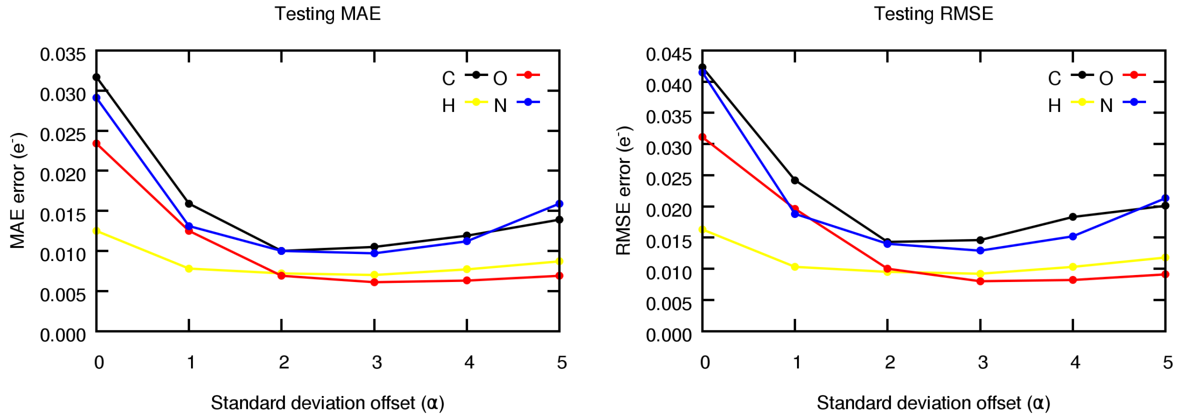

Figure 17: Evolution of the testing MAE and RMSE metrics, in electrons, of the FFNN models trained to predict the atomic charges of peptide-capped alanine as a function of the offset (α) of the displaced Gaussians.

5.5 Binary functions

The binary gaussians were generated starting from the tailor-made clusters of the radial distributions without any further decomposition (cutoff radius of 7.00 Å).

The following table comprises the performance of the models, reported in electrons, trained to predict the atomic charges of peptide-capped alanine as a function of the α and β values used in the binary redistribution schemes.

Table 5: Training (tr) and testing (ts) prediction errors in the estimation of the atomic charges of C atoms for different α and β values. All values are given in electrons.

$\beta(\alpha = 1)$	MAE_{tr}	$RMSE_{tr}$	MAE_{ts}	$RMSE_{ts}$
0.00	0.0118	0.0172	0.0120	0.0174
0.25	0.0104	0.0148	0.0105	0.0149
0.50	0.0102	0.0141	0.0102	0.0141
1.00	0.0093	0.0124	0.0093	0.0124
2.00	0.0084	0.0111	0.0084	0.0109
3.00	0.0082	0.0107	0.0082	0.0106
4.00	0.0082	0.0106	0.0081	0.0105
$\beta(\alpha = 2)$	MAE_{tr}	$RMSE_{tr}$	MAE_{ts}	$RMSE_{ts}$
0.00	0.0099	0.0146	0.0100	0.0150
0.25	0.0102	0.0149	0.0102	0.0151
0.50	0.0100	0.0146	0.0101	0.0149
1.00	0.0095	0.0131	0.0095	0.0131
2.00	0.0086	0.0113	0.0086	0.0113
3.00	0.0082	0.0108	0.0082	0.0107
4.00	0.0082	0.0107	0.0081	0.0106
$\beta(\alpha = 3)$	MAE_{tr}	$RMSE_{tr}$	MAE_{ts}	$RMSE_{ts}$
0.00	0.0085	0.0113	0.0086	0.0112
0.25	0.0085	0.0111	0.0086	0.0114
0.50	0.0084	0.0110	0.0084	0.0110
1.00	0.0081	0.0108	0.0081	0.0107
2.00	0.0081	0.0108	0.0082	0.0108
3.00	0.0081	0.0108	0.0081	0.0108
4.00	0.0081	0.0108	0.0081	0.0108

Table 6: Training (tr) and testing (ts) prediction errors in the estimation of the atomic charges of H atoms for different α and β values. All values are given in electrons.

$\beta(\alpha = 1)$	MAE_{tr}	$RMSE_{tr}$	MAE_{ts}	$RMSE_{ts}$
0.00	0.0068	0.0088	0.0068	0.0088
0.25	0.0068	0.0089	0.0068	0.0089
0.50	0.0067	0.0086	0.0066	0.0086
1.00	0.0064	0.0083	0.0064	0.0083
2.00	0.0064	0.0082	0.0063	0.0082
3.00	0.0063	0.0082	0.0063	0.0082
4.00	0.0064	0.0083	0.0064	0.0083
$\beta(\alpha = 2)$	MAE_{tr}	$RMSE_{tr}$	MAE_{ts}	$RMSE_{ts}$
0.00	0.0069	0.0092	0.0069	0.0093
0.25	0.0069	0.0092	0.0069	0.0092
0.50	0.0069	0.0093	0.0069	0.0092
1.00	0.0068	0.0090	0.0068	0.0090
2.00	0.0063	0.0081	0.0062	0.0080
3.00	0.0062	0.0080	0.0061	0.0079
4.00	0.0062	0.0080	0.0061	0.0079
$\beta(\alpha = 3)$	MAE_{tr}	$RMSE_{tr}$	MAE_{ts}	$RMSE_{ts}$
0.00	0.0066	0.0087	0.0067	0.0086
0.25	0.0066	0.0086	0.0066	0.0085
0.50	0.0065	0.0084	0.0065	0.0084
1.00	0.0062	0.0081	0.0062	0.0081
2.00	0.0062	0.0080	0.0061	0.0080
3.00	0.0062	0.0081	0.0062	0.0081
4.00	0.0062	0.0081	0.0062	0.0080

Table 7: Training (tr) and testing (ts) prediction errors in the estimation of the atomic charges of O atoms for different α and β values. All values are given in electrons.

$\beta(\alpha = 1)$	MAE_{tr}	$RMSE_{tr}$	MAE_{ts}	$RMSE_{ts}$
0.00	0.0064	0.0086	0.0065	0.0088
0.25	0.0063	0.0085	0.0063	0.0085
0.50	0.0061	0.0081	0.0061	0.0081
1.00	0.0059	0.0078	0.0059	0.0077
2.00	0.0057	0.0076	0.0058	0.0075
3.00	0.0058	0.0077	0.0059	0.0077
4.00	0.0058	0.0078	0.0058	0.0078
$\beta(\alpha = 2)$	MAE_{tr}	$RMSE_{tr}$	MAE_{ts}	$RMSE_{ts}$
0.00	0.0072	0.0108	0.0074	0.0111
0.25	0.0074	0.0110	0.0077	0.0113
0.50	0.0071	0.0107	0.0074	0.0111
1.00	0.0077	0.0109	0.0078	0.0112
2.00	0.0057	0.0076	0.0056	0.0074
3.00	0.0057	0.0075	0.0057	0.0074
4.00	0.0057	0.0075	0.0057	0.0075
$\beta(\alpha = 3)$	MAE_{tr}	$RMSE_{tr}$	MAE_{ts}	$RMSE_{ts}$
0.00	0.0059	0.0079	0.0059	0.0078
0.25	0.0059	0.0078	0.0059	0.0078
0.50	0.0058	0.0077	0.0059	0.0077
1.00	0.0058	0.0077	0.0059	0.0076
2.00	0.0057	0.0076	0.0058	0.0076
3.00	0.0058	0.0078	0.0059	0.0078
4.00	0.0058	0.0078	0.0059	0.0079

Table 8: Training (tr) and testing (ts) prediction errors in the estimation of the atomic charges of N atoms for different α and β values. All values are given in electrons.

$\beta(\alpha = 1)$	MAE_{tr}	RMSE_{tr}	MAE_{ts}	RMSE_{ts}
0.00	0.0108	0.0146	0.0110	0.0154
0.25	0.0103	0.0140	0.0104	0.0142
0.50	0.0111	0.0152	0.0112	0.0154
1.00	0.0097	0.0129	0.0097	0.0130
2.00	0.0094	0.0126	0.0093	0.0125
3.00	0.0091	0.0122	0.0090	0.0122
4.00	0.0092	0.0123	0.0092	0.0123
$\beta(\alpha = 2)$	MAE_{tr}	RMSE_{tr}	MAE_{ts}	RMSE_{ts}
0.00	0.0096	0.0131	0.0095	0.0131
0.25	0.0098	0.0134	0.0098	0.0132
0.50	0.0099	0.0135	0.0098	0.0135
1.00	0.0096	0.0132	0.0096	0.0130
2.00	0.0092	0.0124	0.0091	0.0123
3.00	0.0090	0.0121	0.0090	0.0120
4.00	0.0089	0.0119	0.0090	0.0120
$\beta(\alpha = 3)$	MAE_{tr}	RMSE_{tr}	MAE_{ts}	RMSE_{ts}
0.00	0.0093	0.0125	0.0093	0.0127
0.25	0.0091	0.0123	0.0092	0.0126
0.50	0.0091	0.0123	0.0092	0.0125
1.00	0.0090	0.0121	0.0091	0.0123
2.00	0.0088	0.0119	0.0088	0.0119
3.00	0.0088	0.0120	0.0088	0.0120
4.00	0.0089	0.0120	0.0088	0.0119

The following figures show the evolution of the testing MAE and RMSE error metrics in the prediction of the atomic charges of peptide-capped alanine as a function of the α and β values employed for the construction of the binary gaussian functions.

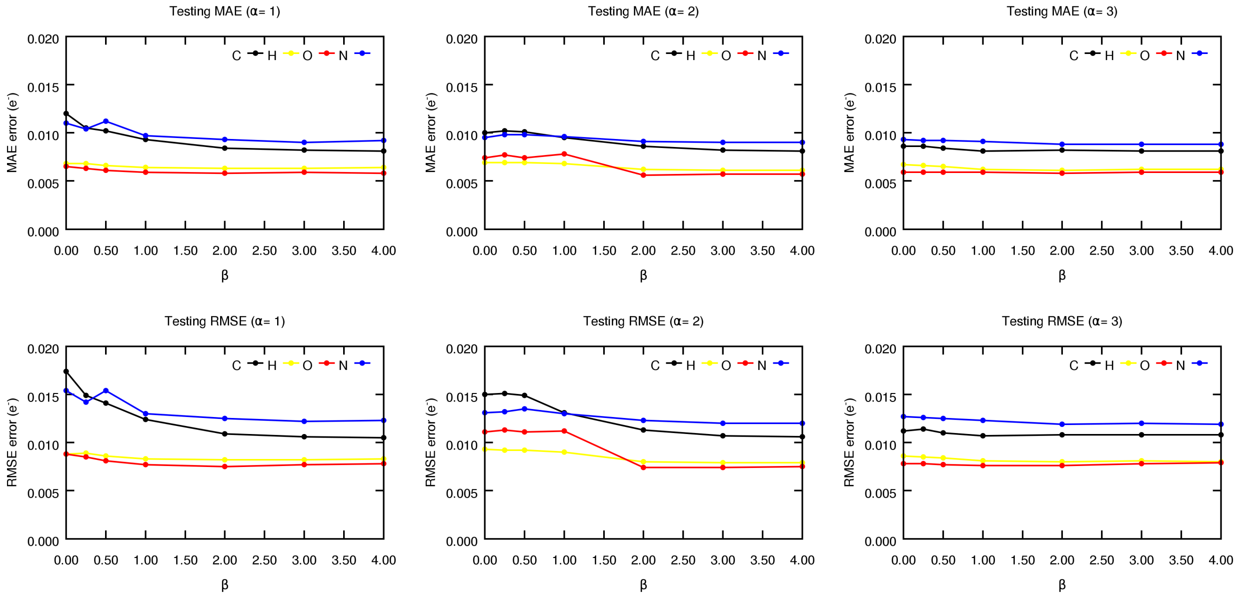

Figure 18: Evolution of the testing MAE and RMSE metrics, in electrons, of the FFNN models trained to predict the atomic charges of peptide-capped alanine as a function of the α and β values employed.

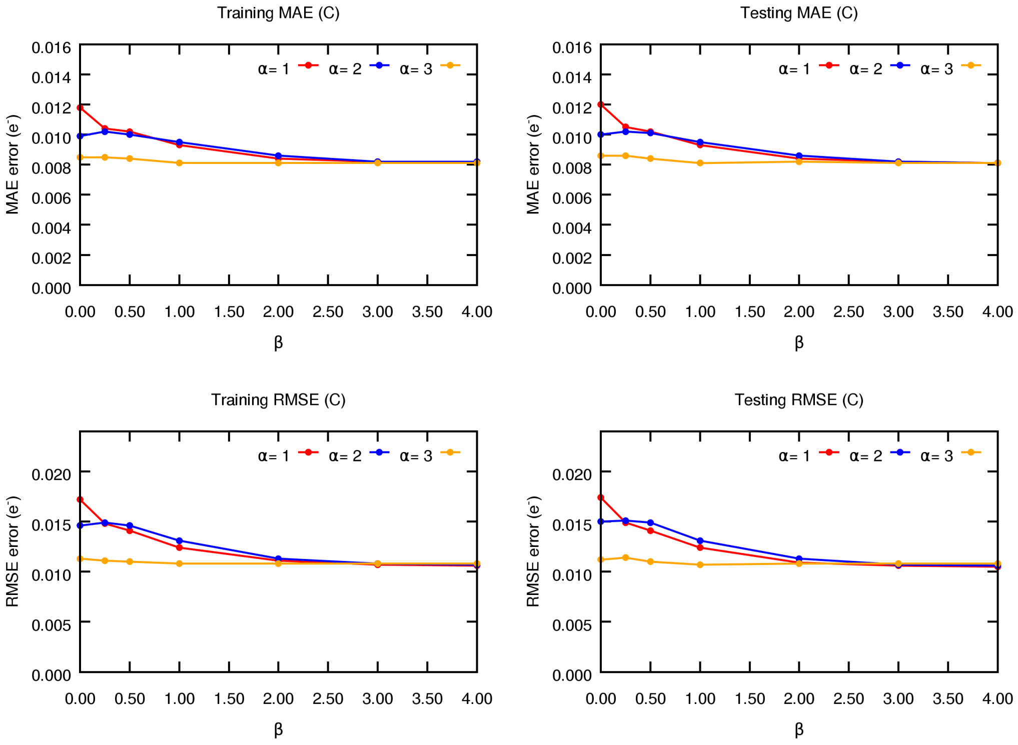

Figure 19: Evolution of the MAE and RMSE metrics, in electrons, of the FFNN models trained to predict the atomic charges of the C atoms in peptide-capped alanine as a function of the α and β values employed.

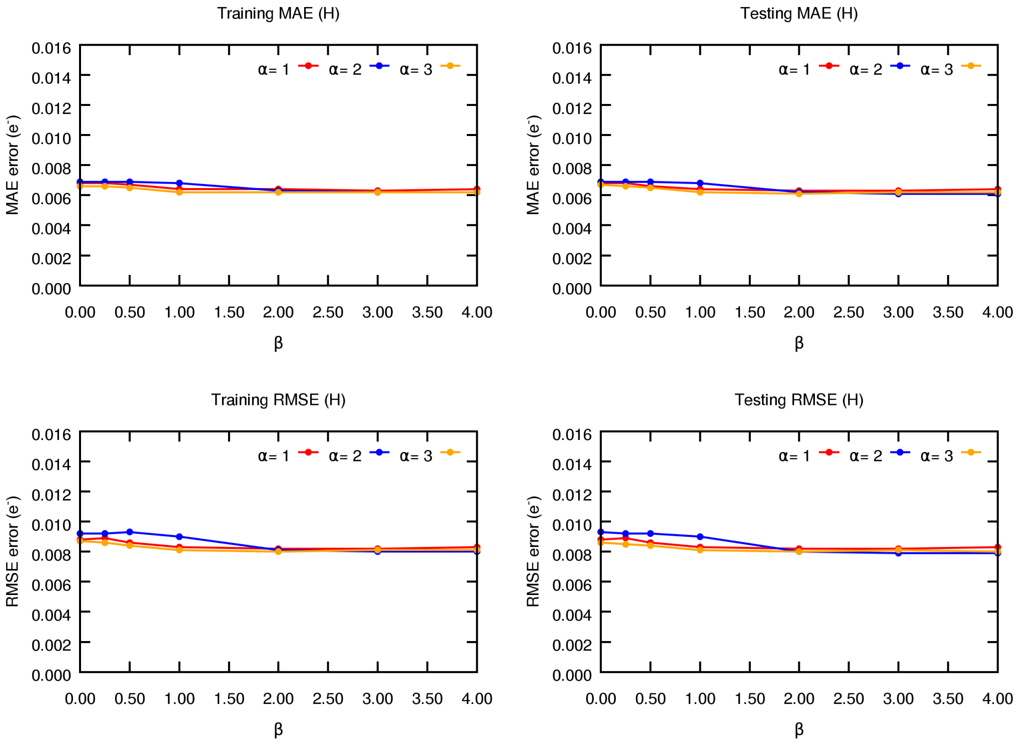

Figure 20: Evolution of the MAE and RMSE metrics, in electrons, of the FFNN models trained to predict the atomic charges of the H atoms in peptide-capped alanine as a function of the α and β values employed.

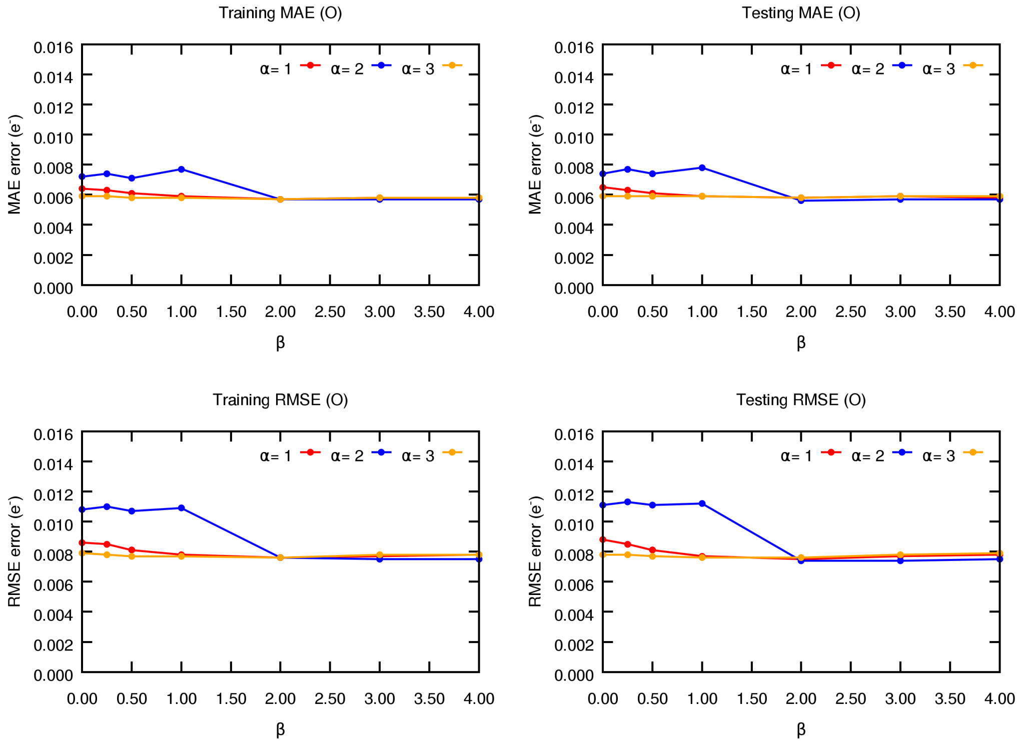

Figure 21: Evolution of the MAE and RMSE metrics, in electrons, of the FFNN models trained to predict the atomic charges of the O atoms in peptide-capped alanine as a function of the α and β values employed.

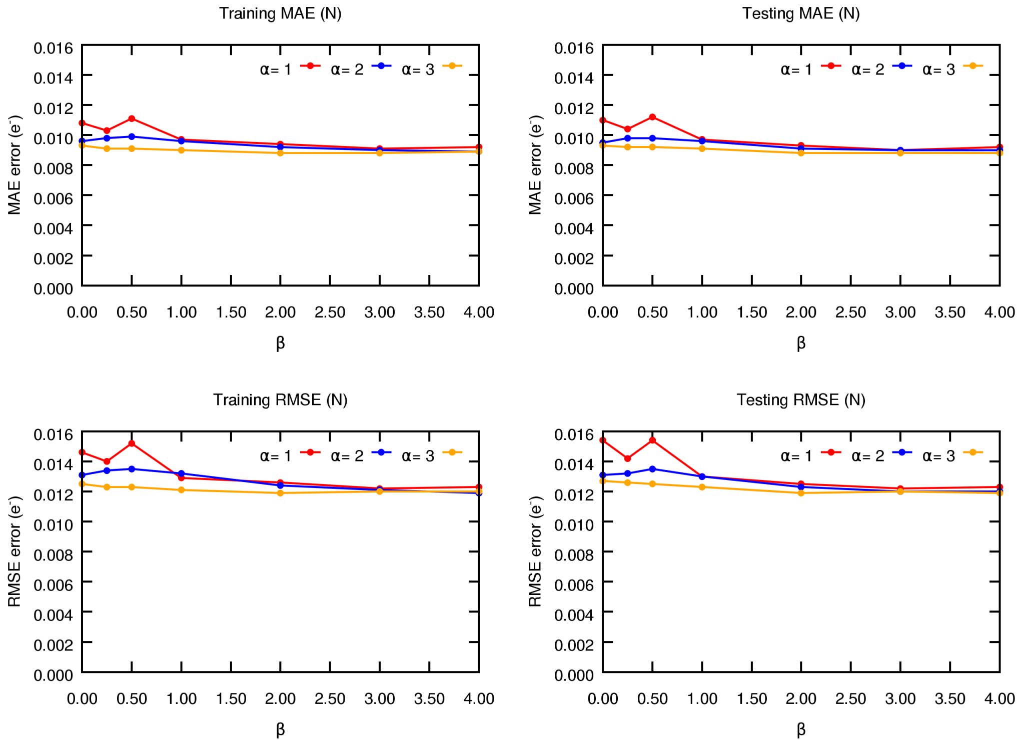

Figure 22: Evolution of the MAE and RMSE metrics, in electrons, of the FFNN models trained to predict the atomic charges of the N atoms in peptide-capped alanine as a function of the α and β values employed.

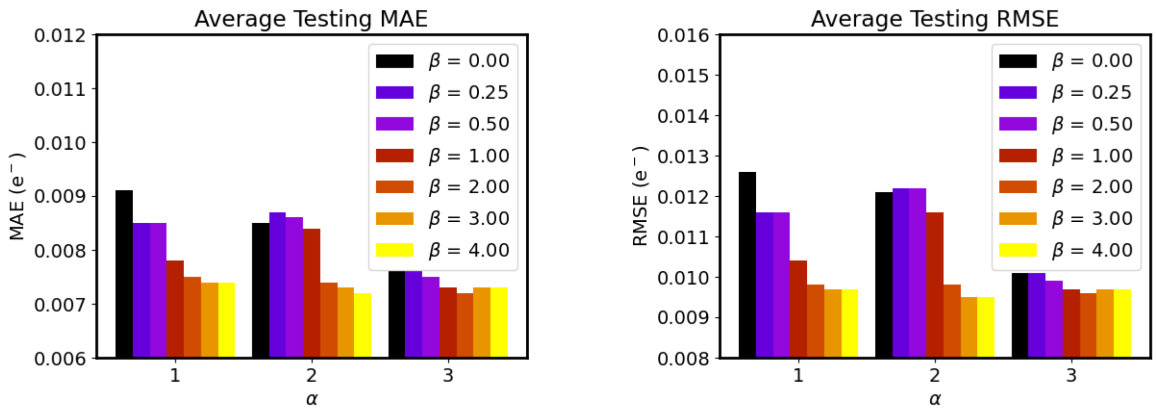

Figure 23: Evolution of the mean testing MAE and RMSE error metrics, in electrons, in the prediction of the atomic charges of peptide-capped alanine as a function of the α and β values employed.

Finally, the following table comprises a comparison of the performance achieved by the FFNN models in the prediction of the atomic charges of peptide-capped alanine using AEVs of the same length generated with different sampling techniques.

Table 9: Training MAE errors, in electrons, for the prediction of the atomic charges of different atoms in peptide-capped alanine as a function of the approach employed to create the radial ACSF functions. All approaches were trained using the same number of features corresponding to 58, 72, 48 and 40 elements for C, H, O and N, respectively.

Element	Tailor-made	Even	Binary (α= 3, β= 1)
C	0.016	0.008	0.008
H	0.008	0.006	0.006
O	0.007	0.006	0.006
N	0.011	0.009	0.009

6 Optimized Angular Symmetry Functions

The current section comprises a summary of the optimization procedure of the angular symmetry functions, according to different schemes.

Table 10: Training (tr) MAE and RMSE error metrics of the FFNN models trained to predict the atomic charges of peptide-capped alanine using a combination of radial and angular ACSF functions. A fixed collection of radial functions (`binary`, α= 3, β= 1) was employed in combination with a varying set of angular terms optimized using different `nmax`, `afrac` and r_c values. All values are given in electrons. The cutoff radius (r_c) is reported in Å.

			r_c= 3.5		r_c= 5.0		r_c= 7.0	
Element	`nmax`	`afrac`	MAE_{tr}	$RMSE_{tr}$	MAE_{tr}	$RMSE_{tr}$	MAE_{tr}	$RMSE_{tr}$
C	10	0.50	0.006	0.008	0.006	0.008	0.006	0.008
		0.75	0.006	0.008	0.006	0.008	0.006	0.008
		1.00	0.006	0.008	0.006	0.008	0.006	0.008
	20	0.50	0.006	0.008	0.006	0.008	0.006	0.008
		0.75	0.006	0.008	0.006	0.008	0.006	0.009
		1.00	0.006	0.008	0.006	0.008	0.006	0.008
H	10	0.50	0.005	0.006	0.005	0.006	0.005	0.006
		0.75	0.005	0.006	0.005	0.006	0.005	0.006
		1.00	0.005	0.006	0.005	0.006	0.005	0.006
	20	0.50	0.005	0.006	0.005	0.006	0.005	0.006
		0.75	0.004	0.006	0.005	0.006	0.005	0.006
		1.00	0.004	0.006	0.005	0.006	0.005	0.006
O	10	0.50	0.005	0.007	0.005	0.007	0.005	0.006
		0.75	0.005	0.007	0.005	0.007	0.005	0.007
		1.00	0.005	0.006	0.005	0.007	0.005	0.007
	20	0.50	0.005	0.006	0.005	0.006	0.005	0.006
		0.75	0.005	0.006	0.005	0.007	0.005	0.007
		1.00	0.004	0.006	0.005	0.007	0.005	0.007
N	10	0.50	0.007	0.010	0.008	0.010	0.008	0.011
		0.75	0.007	0.010	0.008	0.011	0.008	0.011
		1.00	0.007	0.010	0.008	0.011	0.008	0.011
	20	0.50	0.008	0.010	0.008	0.010	0.007	0.010
		0.75	0.008	0.011	0.008	0.010	0.008	0.011
		1.00	0.008	0.011	0.008	0.011	0.008	0.011

Table 11: Testing (ts) MAE and RMSE error metrics of the FFNN models trained to predict the atomic charges of peptide-capped alanine using a combination of radial and angular ACSF functions. A fixed collection of radial functions (`binary`, α= 3, β= 1) was employed in combination with a varying set of angular terms optimized using different `nmax`, `afrac` and r_c values. All values are given in electrons. The cutoff radius (r_c) is reported in Å.

			r_c= 3.5		r_c= 5.0		r_c= 7.0	
Element	`nmax`	`afrac`	MAE_{ts}	$RMSE_{ts}$	MAE_{ts}	$RMSE_{ts}$	MAE_{ts}	$RMSE_{ts}$
C	10	0.50	0.006	0.008	0.006	0.008	0.006	0.008
		0.75	0.006	0.008	0.006	0.008	0.006	0.009
		1.00	0.006	0.009	0.006	0.008	0.006	0.009
	20	0.50	0.006	0.008	0.006	0.008	0.006	0.009
		0.75	0.006	0.008	0.006	0.009	0.006	0.009
		1.00	0.006	0.008	0.006	0.008	0.006	0.008
H	10	0.50	0.005	0.006	0.005	0.006	0.005	0.006
		0.75	0.005	0.006	0.005	0.006	0.005	0.006
		1.00	0.005	0.006	0.005	0.006	0.005	0.006
	20	0.50	0.005	0.006	0.005	0.006	0.005	0.006
		0.75	0.004	0.006	0.005	0.006	0.005	0.006
		1.00	0.004	0.006	0.005	0.006	0.005	0.006
O	10	0.50	0.005	0.007	0.005	0.007	0.005	0.007
		0.75	0.005	0.007	0.005	0.007	0.005	0.007
		1.00	0.005	0.007	0.005	0.007	0.005	0.007
	20	0.50	0.005	0.007	0.005	0.007	0.005	0.006
		0.75	0.005	0.006	0.005	0.007	0.005	0.007
		1.00	0.005	0.006	0.005	0.007	0.005	0.007
N	10	0.50	0.007	0.011	0.008	0.012	0.008	0.012
		0.75	0.008	0.011	0.008	0.012	0.008	0.011
		1.00	0.008	0.011	0.008	0.011	0.008	0.012
	20	0.50	0.008	0.011	0.008	0.011	0.008	0.011
		0.75	0.008	0.012	0.008	0.011	0.008	0.011
		1.00	0.008	0.012	0.008	0.012	0.008	0.012

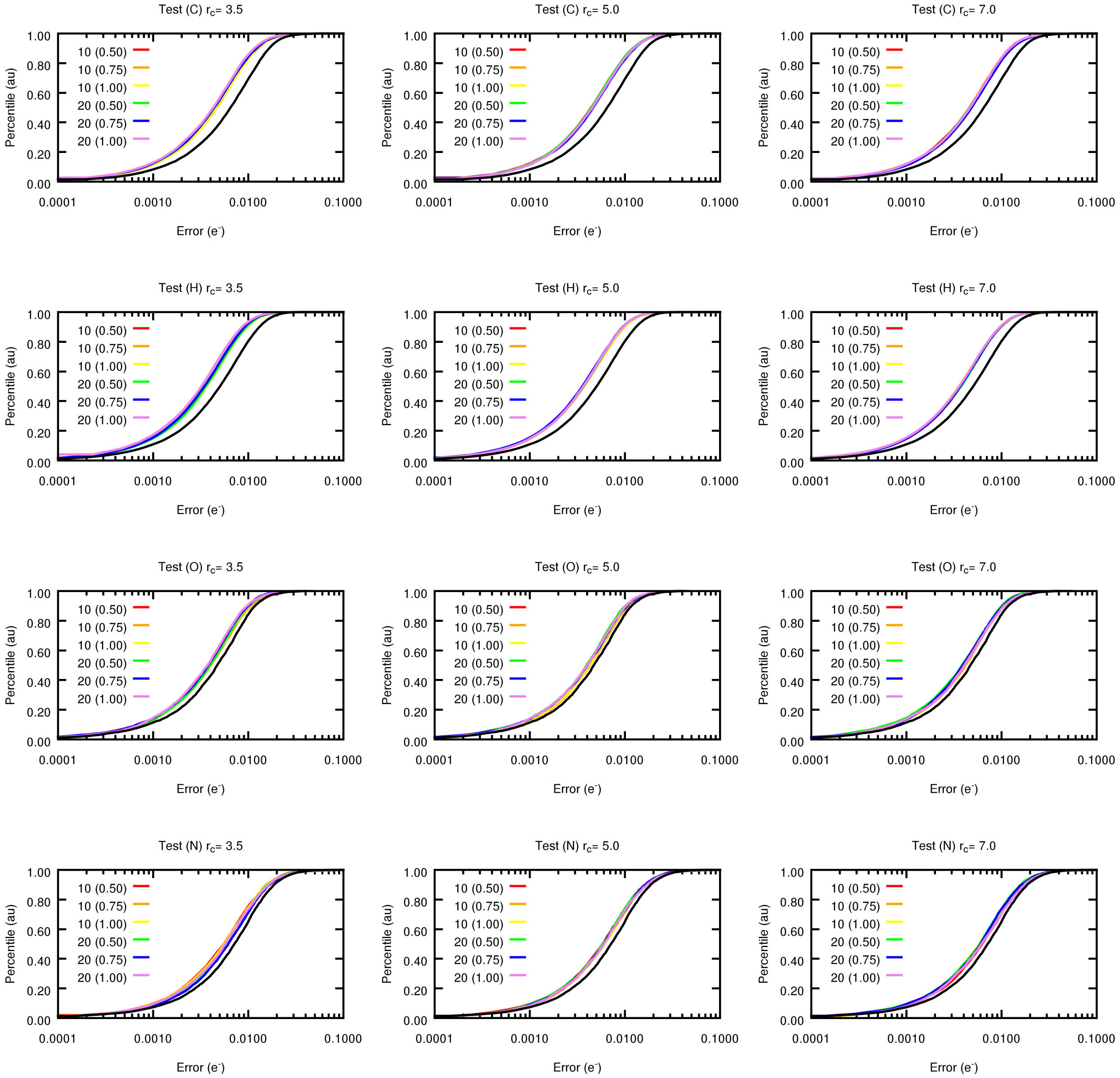

Figure 24: S-curves for the predicted atomic charges of peptide-capped alanine using the optimized radial and angular symmetry functions. A fixed collection of radial functions (binary, α= 3, β= 1) was employed in combination with a varying set of angular terms optimized using different `nmax`, `afrac` and r_c values. All values are given in electrons. For the sake of comparison, the S-curves for the models trained exclusively on the binary radial functions (black curve) are also shown. The cutoff radius (r_c) is reported in Å.

7 Performance enhancement using FEREBUS

7.1 Computational details and methodology: FEREBUS

FEREBUS is a Gaussian process regression (GPR) engine written in Fortran90 and accelerated via OpenMP. Since its earlier versions, the program has been considerably improved in terms of design, functionalities, and numerical stability. Besides the design and integration of an in-house software module (STELLA) for on-the-fly model validation and testing, FEREBUS has been equipped with a simple and intuitive training protocol called iterative hold-out cross-validation (IHOCV). Unlike the popular Type-II maximum likelihood (ML-II) approach (also available in FEREBUS), whose performance is often compromised by the presence of outliers in the datasets,[10] the IHOCV protocol is less affected in case of model misspecification and outperforms ML-II in terms of both the predictive accuracy of the trained models and numerical stability (especially in the noise-free regime).[11] To find the optimal set of GPR model hyperparameters (denoted as $\hat{\theta}$), the IHOCV protocol proceeds by minimizing the predictive (root) mean squared error of intermediary models over a fixed internal validation set. The MSE(θ) loss function is given by,

$$MSE(\theta) = \frac{1}{M}\sum_{j=1}^{M}\left(q_j^{true} - q_j^{pred}\right)^2, \tag{20}$$

where M is the number of internal validation points, q_j^{true} and q_j^{pred} the true and predicted atomic charges of the jth validation point, respectively.

Assuming a GPR model has already been trained on a dataset $D = \{X, y^{true}\}$ of N observations, predictions on unseen points can be made as,

$$q_j^{pred} = q^{prior} + \sum_{i=1}^{N}\omega_i k\left(x_i, x_j; \hat{\theta}\right), \tag{21}$$

where q^{prior} is the prior mean function, ω_i the ith element of the weights vector and k $(x_i, x_j; \theta)$ the similarity between the test point j and the ith training point. Note that the prior mean function is chosen here to be constant and equal to the arithmetic mean of q^{true} values in the training set. This choice guarantees that predictions remain physically meaningful, even in the undesired extrapolation regime. On the other hand, the weights vector is obtained by solving the system of linear equations,

$$K(\theta)\omega = \mathbf{q^{true}} - \mathbf{1}q^{prior}, \tag{22}$$

and,

$$K_{ij} = k\left(x_i, x_j; \theta\right) + \sigma_n^2\delta_{ij}, \tag{23}$$

where $\mathbf{q^{true}}$ is the vector of true atomic charges (the target values), K the covariance matrix, σ_n^2 the regularization noise, k $(x_i, x_j; \theta)$ the kernel, $\mathbf{1}$ a column vector of ones and δ the Kronecker delta.

The following squared exponential kernel was chosen to assess similarities between points within the input space. This common kernel is determined by two series of parameters (or model hyperparameters), the characteristic length-scales l_d and the prefactor term σ^2, which combined with the regularization noise σ_n^2 constitute the hyperparameters of the model. Without any loss of generality, σ^2 was fixed at 1 while the l_d's and σ_n^2 hyperparameters were fully optimized.

$$k\left(x_i, x_j; \theta\right)_{SE} = \sigma^2 \exp\left(\sum_{d=1}^{N_{feats}} -\frac{\left(x_i^d - x_j^d\right)^2}{2l_d^2}\right), \tag{24}$$

where N_{feats} is the number of input features.

The MSE(θ) loss function was minimized using an enhanced gray wolf optimizer (GWO) recently proposed by the authors.[12] Like vanilla GWO,[13] our GWO-RUHL is a population-based metaheuristic optimizer inspired by the leadership hierarchy and hunting strategy of gray wolves. It utilizes a certain number of agents to scrutinize the search space. Each agent is encoded as a vector of the same dimension as the hyperparameter space and constitutes a candidate solution. The best three candidate solutions or leaders correspond to the α, β, and δ wolves, while the non-leader agents are associated with ω wolves. Because the leaders know better where the optimal solution might be located, they share their position (at each iteration) with all ω wolves to help them move in the right direction. The position X_j of a given ω wolf j is updated using equations 25-28, where X_{Tp} $(p = \alpha, \beta, \delta)$ is a temporary vector

pointing in the direction of each of the three leaders. The two vectors A and C both store three random numbers denoted as A_p and C_p. Their role is to introduce some amount of stochasticity in the optimization process, necessary for jumping out of local optima. Equations 27-28 indicate that A_p and C_p depend on two independent random numbers $r_{1,p}$ and $r_{2,p}$ (whose values are uniformly sampled within the range [0,1]). The term a_p decreases linearly from 2 to 0 during the optimization process.

$$D_p(t) = |C_p(t)X_p(t) - X_j(t)|. \tag{25}$$

$$X_{Tp}(t+1) = X_p(t) - A_p(t)D_p(t). \tag{26}$$

$$A_p(t) = 2a_p(t)r_{1,p} - a_p(t). \tag{27}$$

$$C_p(t) = 2r_{2,p}. \tag{28}$$

The new position of an omega wolf is obtained as an average displacement toward each of the three leaders as,

$$X_j(t+1) = \frac{1}{3}\sum_p X_{Tp}(t+1). \tag{29}$$

As an improvement over the standard GWO, the GWO-RUHL algorithm incorporates in the previous search mechanism the natural desire of ω wolves to occupy high-ranked positions in the leadership hierarchy. This is achieved thanks to a new operator $\hat{U}$ which acts on the current population $P\{X_g|g \in G\}$ (set of candidate solutions) and promotes a certain number n of ω solutions, each to a new position situated in the vicinity of the center of mass of the three leaders (every p iterations). In the perturbed population, n vectors are replaced by $n^{'}$ new ones. Each new solution $X_q(t+1)^{'}$ is derived as indicated in the following equations, where Δ_q is a tiny deviation from the leaders' center of mass.

$$\hat{U}P = P^{'}, \tag{30}$$

$$X_q(t+1)^{'} = P_{cm} + \Delta_q, \tag{31}$$

$$P_{cm} = \frac{\sum_p m_p X_p}{3}; \Delta_q = |X_\alpha - P_{cm}| \times \epsilon\,(r, s)\,, \tag{32}$$

where P_{cm} is the center of mass of the leaders and $\epsilon_{r,s}$ a random number drawn between $r = 0$ and $s = 1$ (these values can be adjusted by the user). By default, the weighting scheme has been set as $\{m_p = 1|p = \alpha, \beta, \delta\}$, which assumes that all leader wolves have the same mass (or better weight). However, there is no harm in specifying any other weighting scheme that would for instance assign weights such as $m_\alpha > m_\beta > m_\delta$. One can show that the new solutions $\{X_q(t+1)^{'}|q = 1, ..., n\}$ are all viable as they remain in the search space.

Unless otherwise stated, all FEREBUS models were trained on 1000 to 6000 points (randomly sampled from the 15000 points) and tested on the same 2000 points used for testing the FFNN models. An internal validation set of 250 points was employed to guide the optimization process. Its size was chosen to ensure a reasonable compromise between model performance and computational cost. The search space was defined as $[1.0, \infty]$ and $[10^{-14}, 10^{-4}]$ for l_d and σ_n^2 hyperparameters, respectively, while a fixed number of 20 agents were tasked to explore the previous search space for a maximum of 1000 iterations. Furthermore, to accelerate the exploration of promising regions of the hyperparameter space, one lucky agent was promoted every iteration, keeping in mind that promoting many agents every iteration could lead to premature convergence. Finally, all the calculations were performed in parallel using 20 cores on compute nodes with Cascade Lake Xeon Gold 6230 CPUs of 2.10 GHz each.

Table 12: Performance of FEREBUS models in predicting the atomic charges of peptide-capped alanine for the smallest training set (1000 points). The q^{range} parameter denotes the absolute difference between the maximum and minimum atomic charges in the training set (and the entire population in parentheses) for a given atom. All quantities are given in electrons.

Atom	RMSE	MAE	q^{range}	Atom	RMSE	MAE	q^{range}
O1	0.0063	0.0049	0.26(0.28)	H12	0.0088	0.0047	0.11(0.12)
N2	0.0093	0.0069	0.44(0.50)	C13	0.0059	0.0043	0.37(0.48)
C3	0.0079	0.0061	0.37(0.45)	H14	0.0046	0.0034	0.17(0.26)
C4	0.0087	0.0067	0.24(0.33)	H15	0.0045	0.0034	0.16(0.20)
C5	0.0079	0.0055	0.54(0.73)	H16	0.0055	0.0038	0.15(0.26)
H6	0.0070	0.0053	0.13(0.22)	C17	0.0087	0.0058	0.25(0.30)
H7	0.0065	0.0051	0.19(0.24)	H18	0.0069	0.0049	0.20(0.27)
H8	0.0065	0.0050	0.21(0.26)	H19	0.0064	0.0047	0.18(0.20)
H9	0.0066	0.0051	0.20(0.28)	H20	0.0066	0.0048	0.15(0.24)
H10	0.0065	0.0043	0.14(0.17)	C21	0.0068	0.0048	0.48(0.67)
N11	0.0103	0.0074	0.40(0.46)	O22	0.0071	0.0053	0.22(0.34)

The performance of atomic models, illustrated here in Table 12, consistently demonstrates the effect of the local environment on the quality of these models. Because models must somehow learn the symmetry of a system, the only situation where one should expect two atomic models to exhibit identical performances is when the corresponding atoms are chemically equivalent, i.e., the same type and spatial neighborhood. Although some of the 22 atoms of peptide-capped alanine are obviously more similar than others from a functional perspective (for instance, all H atoms in CH_3 groups), none of them is strictly equivalent to another (for symmetry reasons). Yet, some atomic models reported in Table 12 still exhibit almost identical predictive accuracy. One reason is that when it comes to non-linear interpolation methods such as GPR, the range of the target property often dictates how challenging it is to interpolate. Therefore, two models that learn similar (but different) charge fluctuations might demonstrate the same (distinct) interpolation capabilities for the same training set size. This argument can be evoked to explain the findings of Burn and Popelier,[14] who observed that atomic GPR models trained on high-temperature MD data required more points than their low-temperature analogs to achieve the desired performance due to more pronounced fluctuations of local properties at high temperature. However, beyond the range of the target property, the nature of the atom itself clearly plays a prime role. In fact, Table 12 indicates that, while it is the charge on C5 that fluctuates the most ($q^{range} = 0.72$ e), it is the charge on N11 that seems to be the most challenging to reproduce ($q^{range} = 0.50$ e). This counter-intuitive observation advocates for the intricate character of Nitrogen.

7.2 Performance metrics

Table 13 reports the predictive accuracy of FEREBUS and FFNN models trained on 1000 to 6000 points using the best-performing ACSFs (i.e a combination of radial ($\alpha = 3$, $\beta = 1$) and angular features). Since FEREBUS naturally builds atom-wise models, the element-wise metrics presented here were derived as averages over atomic models of the same element.

Table 13: Performance of FEREBUS (GP) and FFNN models in predicting atomic charges of peptide-capped alanine. All the models were trained and tested on the same exact datasets, using the best-performing ACSFs. The test metrics are given in electrons.

N_{train}	Element	$RMSE_{GP}$	MAE_{GP}	$RMSE_{NN}$	MAE_{NN}
1000	C	0.0075	0.0054	0.0104	0.0072
	H	0.0061	0.0045	0.0068	0.0050
	O	0.0065	0.0050	0.0118	0.0075
	N	0.0107	0.0078	0.0303	0.0135
2000	C	0.0071	0.0051	0.0098	0.0067
	H	0.0056	0.0041	0.0065	0.0048
	O	0.0063	0.0048	0.0079	0.0059
	N	0.0094	0.0069	0.0180	0.0101
3000	C	0.0070	0.0049	0.0091	0.0064
	H	0.0052	0.0039	0.0066	0.0047
	O	0.0061	0.0047	0.0085	0.0061
	N	0.0086	0.0063	0.0214	0.0109
4000	C	0.0062	0.0044	0.0082	0.0058
	H	0.0051	0.0037	0.0064	0.0047
	O	0.0061	0.0046	0.0070	0.0051
	N	0.0084	0.0061	0.0216	0.0106
5000	C	0.0062	0.0044	0.0084	0.0060
	H	0.0049	0.0036	0.0059	0.0043
	O	0.0059	0.0044	0.0067	0.0049
	N	0.0081	0.0060	0.0118	0.0080
6000	C	0.0060	0.0043	0.0080	0.0056
	H	0.0050	0.0035	0.0067	0.0047
	O	0.0057	0.0044	0.0067	0.0050
	N	0.0079	0.0057	0.0135	0.0085

Similarly, Table 14 presents the maximum absolute errors (maxAE) of FEREBUS models trained on an increasing number of points. Also reported there are the mean absolute errors (MAEs) for the reconstruction of the molecular charge.

Table 14: Maximum absolute errors of FEREBUS models trained on 1000-6000 points and corresponding mean absolute errors for the reconstruction of molecular charges (for both FEREBUS and FFNN). All values are given in electrons.

N_{train}	C	H	O	N	MAE^{GP}_{mol}	MAE^{NN}_{mol}
1000	0.0734	0.0649	0.0405	0.0962	0.026	0.035
2000	0.0601	0.0569	0.0369	0.0855	0.024	0.030
3000	0.0732	0.0471	0.0359	0.0902	0.023	0.031
4000	0.0587	0.0464	0.0370	0.0738	0.022	0.029
5000	0.0535	0.0444	0.0341	0.0684	0.022	0.026
6000	0.0569	0.0604	0.0333	0.0821	0.022	0.027

8 Impact of the sampling procedure

In this section, the impact of the approach employed to sample the potential energy surface of the target systems on the quality of the resultant features will be addressed. More specifically, we have decided to focus on two of the most widely employed techniques for this purpose, namely Normal Mode Sampling (NMS) and Molecular Dynamics (MD) simulations.

The NMS exploration of the space was performed by distorting the equilibrium geometry (optimized at the B3LYP/6-311G level of theory in the gas phase) as dictated by its normal vibration modes at different temperatures. A total of 10 000 frames were generated for each compound. The geometry optimization and frequency calculation were performed with the Gaussian09[1] quantum chemistry package. On the other hand, and for the sake of computational cost, the Molecular Dynamics (MD) sampling of the space was performed at the GFN2-xTB level as implemented in the Atomic Simulation Environment (ASE) python package. Each simulation was propagated for 500 ps with a time step of 1 fs. The temperature of the simulation was controlled using the Langevin thermostat with a friction coefficient of 0.01/fs. Snapshots (frames) were saved every 50 fs, leading to a generous population of 10 000 geometries per compound at a given temperature. Unless otherwise specified throughout the text, the MD and NMS samplings of the space were performed at a fixed temperature of 500 K.

The analysis of the explored chemical spaces was performed with the presented here code. For the radial and angular environments, a cutoff radius of 7.0 and 3.5 Å, respectively, was used throughout. Geometries were sampled every 5 frames from the previously generated pool of trajectories, computed by means of NMS or MD simulations. Each radial environment was decomposed in as much as 15 clusters. On the other hand, a total of 20 clusters were searched for each angular domain. Finally, the following parameters were employed to find the optimum number of angular components: `mm_crit= bicconv, afrac=0.75, percbic=30, percdiff=40`.

8.1 Normal Mode Sampling (NMS)

Normal Mode Sampling (NMS) is a procedure commonly employed to sample the near-equilibrium space of a given system. In NMS, the reference (optimized) geometry is distorted as dictated by its normal vibration modes which should be able to reliably capture the conformational space visited by the molecular backbone at a given temperature.

From a practical point of view, the molecular geometry, comprising a total of N atoms, is first optimized at a given level of theory, followed by the analysis of the eigenvalues of its Hessian matrix. Doing so affords the set of 3N-5 or 3N-6 normal vibration modes (N_f) for linear and non-linear systems, respectively. Each normal vibration mode i is characterized by a set of coordinates, q_i, and a force constant, K_i. The latter represents the second derivative of the molecular energy with respect to an infinitesimal displacement in the atomic coordinates. Then, a set of numbers are drawn from a random distribution for each of the normal modes, c_i, such that $\sum_i^{N_f} c_i = 1 \in [0, 1]$. These are subsequently used to apply random distortions of the molecular skeleton around its equilibrium geometry, the displacement is estimated as,

$$R_i = \pm\sqrt{\frac{3c_i N k_b T}{K_i}}, \tag{33}$$

where k_b is the Boltzmann constant and T is the desired temperature of the ensemble in Kelvin. The sign of the previous displacement is randomly chosen according to a Bernoulli distribution (p= 0.5) to provide a homogeneous sampling of both sides of the potential well. It is worth mentioning that, for the sake of convenience, a lower threshold in the force constant is often imposed so as to ensure a minimum displacement for each normal vibration mode. Applying this procedure for each of the normal vibration modes allows to obtain the net displacement matrix, $\mathbf{Q^R} = \sum^{N_f} \mathbf{q_i^R}$, where $\mathbf{q_i^R}$ is the displacement matrix attributed to each normal vibration mode,

$$\mathbf{q_i^R} = R_i \cdot \mathbf{q_i}, \tag{34}$$

where the latter expression represents a scalar multiplication of the R_i displacement and the coordinate matrix attributed to the ith normal mode, $\mathbf{q_i}$. Altogether, the NMS approach allows to explore the various molecular conformations within the near-equilibrium space that can be populated at a given T. Naturally, the greater the T the larger the geometrical distortion with respect to the optimized geometry.

8.2 Molecular Dynamics vs Normal Mode Sampling

Let's now explore the exhaustiveness with which the conformational space is sampled by the NMS and MD simulations. To obtain a robust picture of the quality of these techniques, various molecular systems, accounting for different connectivities, rigidity and versatility, were used as prototypical models.

The following figure gathers a representation of the selection of molecules used throughout, along with the conformational space sampled by the NMS and MD approaches at 500 K (as represented by the mist-plots).

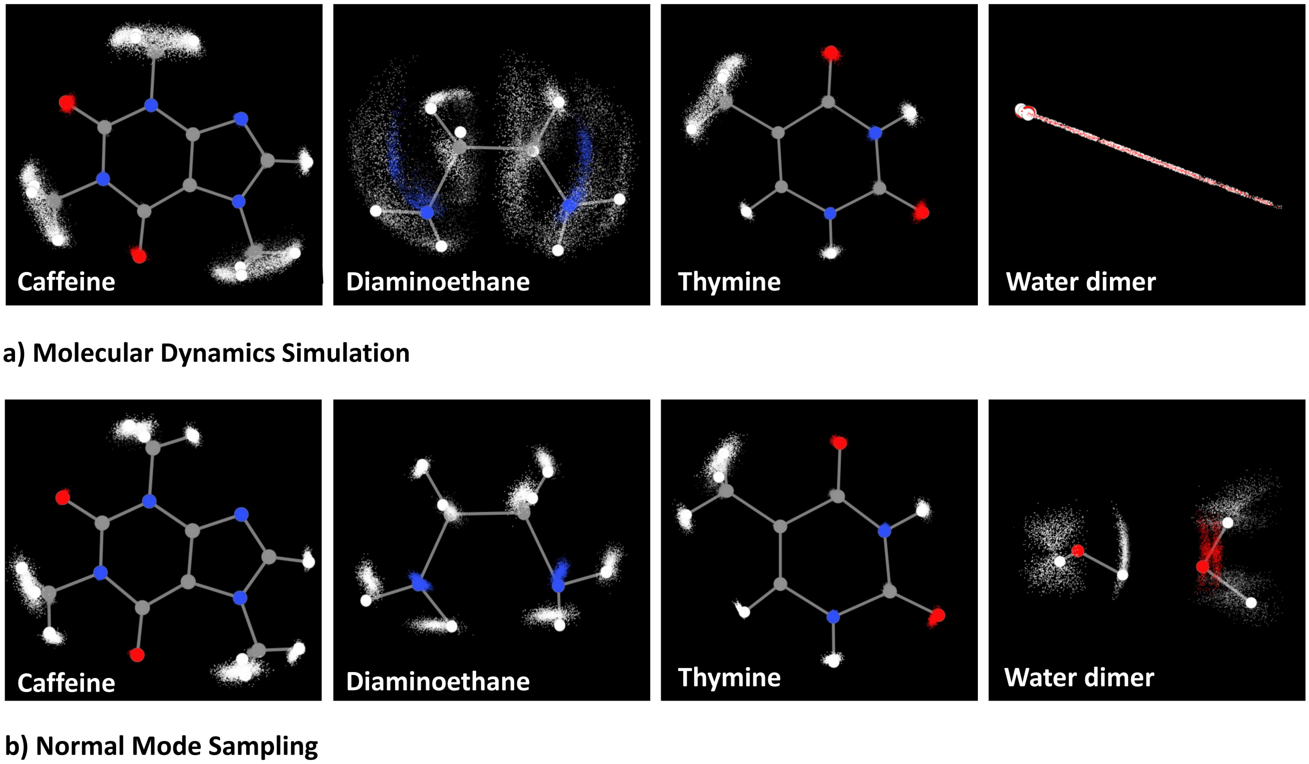

Figure 25: Mist-plots showing the conformational space sampled by the NMS and MD methodologies of different molecular scaffolds, at a temperature of 500 K. For a given system, both plots are centered with respect to the same reference atom. Images generated with Matplotlib[5].

From the previous figure it becomes evident that, in the general case, both NMS and MD techniques tend to explore similar regions of the conformational space. However, MD seems to afford a more extensive sampling, specially around the rotatable σ bonds, as for instance found in the case of the CH_3 moieties. In fact, the differences between both techniques become particularly pronounced for the highly flexible scaffolds, whereas the main backbone of rigid molecules seems to be equally sampled by both strategies. This result is unsurprising, as NMS is inherently limited by its tendency to oscillate around the initial equilibrium position, thus unable to capture degenerate or quasi-degenerate wells present in the PES. In contrast, MD, which explicitly evaluates the system's energy at each frame, proves capable of handling these redundant or equivalent scenarios. This observation is further evidenced from the mean atomic displacements, gathered from the following tables. As such, H atoms, generally embedded in freely movable groups, are often the ones for which MD yields considerably larger mean displacements. Instead, the C atoms, generally comprising the more rigid backbone of the molecule, achieve a similar sampling by both techniques.

Table 15: The mean atomic displacements (μ) and standard deviations (σ) of the different molecules at 500 K throughout the Molecular Dynamics (MD) sampling of the PES. All values, corresponding the average for all the atoms of a given chemical element, are reported in Å.

Molecule	μ_H	μ_O	μ_C	μ_N	σ_H	σ_O	σ_C	σ_N
caffeine	1.23	0.32	0.17	0.17	0.59	0.19	0.09	0.10
diaminoethane	1.33	–	0.32	0.88	0.63	–	0.13	0.48
thymine	0.77	0.34	0.15	0.18	0.38	0.19	0.08	0.10
water dimer	23.50	23.13	–	–	12.59	12.36	–	–

Table 16: The mean atomic displacements (μ) and standard deviations (σ) of the different molecules at 500 K throughout the Normal Mode Sampling (NMS) sampling of the PES. All values, corresponding the average for all the atoms of a given chemical element, are reported in Å.

Molecule	μ_H	μ_O	μ_C	μ_N	σ_H	σ_O	σ_C	σ_N
caffeine	0.45	0.21	0.15	0.14	0.22	0.13	0.07	0.08
diaminoethane	0.36	–	0.12	0.24	0.15	–	0.04	0.08
thymine	0.27	0.26	0.12	0.18	0.14	0.14	0.05	0.08
water dimer	0.54	0.27	–	–	0.25	0.11	–	–

Finally, a particularly noteworthy observation arises in the case of the water dimer: at moderately high temperatures, the available thermal energy becomes sufficient to overcome the existing potential well. In the MD simulation, this leads to a partially unbounded system that effectively behaves as two isolated water monomers. This is clearly evidenced by both the mist plots and the aforementioned tables, which show mean atomic displacements of approximately 20 Å. Once again, the NMS technique is constrained to explore the near-equilibrium space based on a harmonic potential around the well, thereby preventing the observed effect in the MD simulation. We thus expect NMS to become particularly handy to roughly explore the equilibrium PES of, specially weakly bounded, intermolecular systems. Altogether, at a given temperature, MD appears to enable a more aggressive sampling of the space, extending further from the starting equilibrium point. Additionally, it exhibits the capability to, at least partially, consider the presence of degenerate or nearly-degenerate critical points in the PES.

Radial environments

After exploring the extent to which the space gets sampled by the NMS and MD simulations, let's check the impact of the sampling approach on the actual radial and angular environments.

The following figures show a comparison of the radial distribution of a selection of relevant atomic pairs for each of the systems, as sampled by the NMS and MD trajectories at 500 K. Black and red lines show the observed and reconstructed radial distributions, the latter arising from the sum of the individual clusters identified by the GMM models (shown in blue).

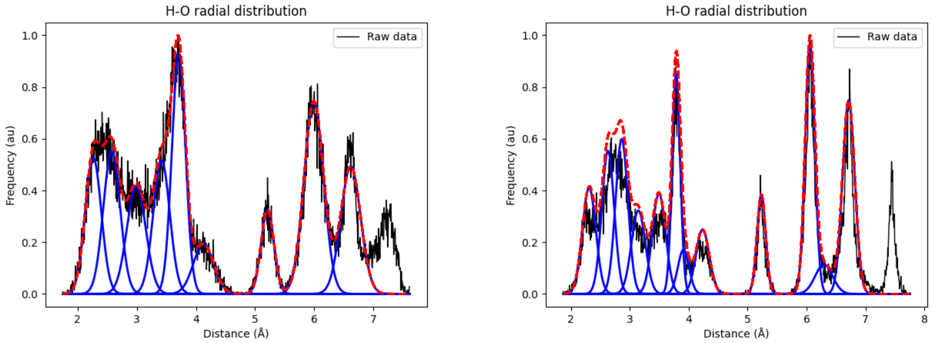

Figure 26: H-O radial distributions (500 K) of the caffeine molecule, using a cutoff radius of 7.0 Å, as sampled by the MD (left) and NMS (right) simulations.

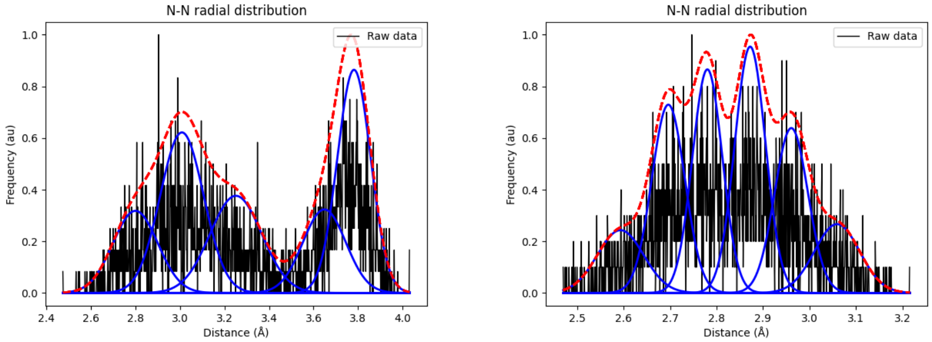

Figure 27: N-N radial distributions (500 K) of the diaminoethane molecule, using a cutoff radius of 7.0 Å, as sampled by the MD (left) and NMS (right) simulations.

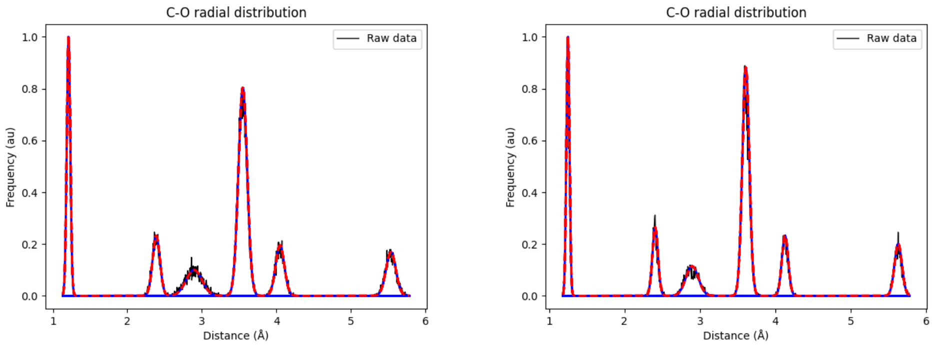

Figure 28: C-O radial distributions (500 K) of the thymine molecule, using a cutoff radius of 7.0 Å, as sampled by the MD (left) and NMS (right) simulations.

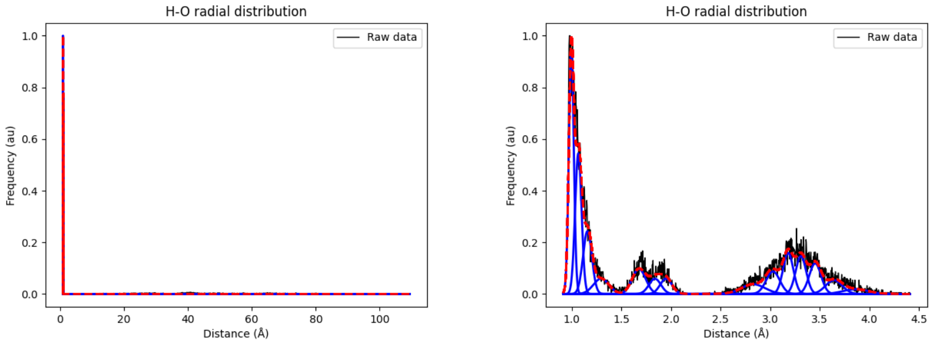

Figure 29: H-O radial distributions (500 K) of the water dimer molecule, using a cutoff radius of 7.0 Å, as sampled by the MD (left) and NMS (right) simulations.

As can be seen from the previous figures, and despite the aforementioned differences, the NMS and MD simulations generally afford similar radial environment distributions. That said, as anticipated, the utilization of MD appears to result in broader distributions, stemming from the more aggressive bond shortening and elongation phenomena associated with the more thorough sampling of the PES. On top of that, the pairwise radial distributions attributed to highly flexible and rotatable bonds do show discrepancies for both techniques, with MD covering a wider range of values. This can be clearly seen for instance in the case of the N-N distribution of diaminoethane, for which MD exhibits an additional cluster centered around 3.8 Å corresponding to the transition between *gauche*, *staggered* and *eclipsed* rotational conformers of this system. By the same token, the previously mentioned cleavage of the water dimer through the MD simulation is directly reflected in the H-O MD radial distributions which tend to (nearly) infinitely spread through the space. Contrarily, and in agreement with the trends found throughout the mist plots, the NMS sampling of the water dimer results in well-defined clusters corresponding to the sampling of the intra- and inter-molecular H-O pairwise distances.

Angular environments

The following figures show a comparison of the angular distribution of a selection of relevant atomic trios for each of the systems, as sampled by the NMS and MD trajectories at 500 K.

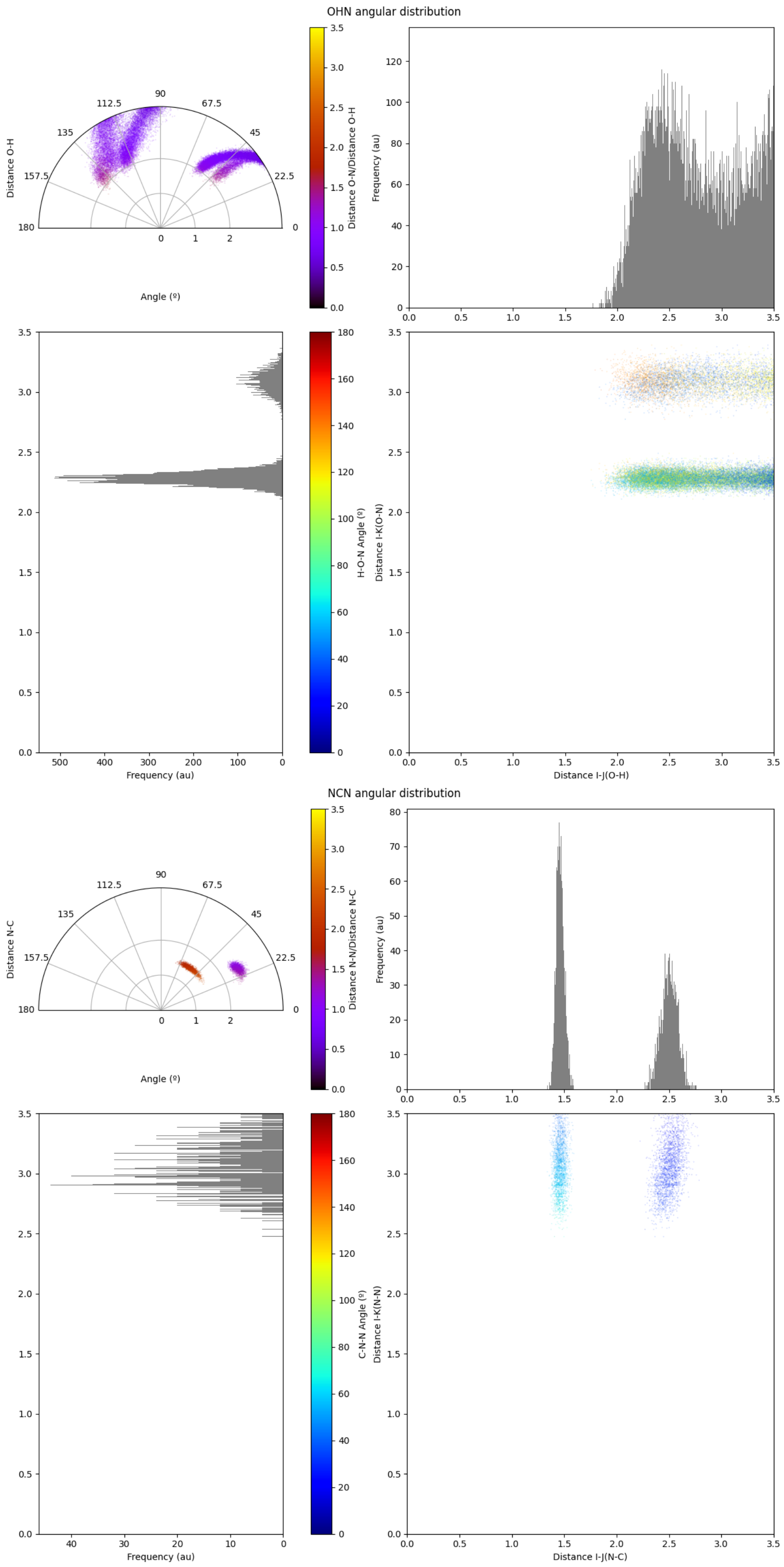

Figure 30: O-H-N and N-C-N angular distributions (500 K) of the caffeine (top) and diaminoethane (bottom) molecules, using a cutoff radius of 3.5 Å, as sampled by the MD simulation.

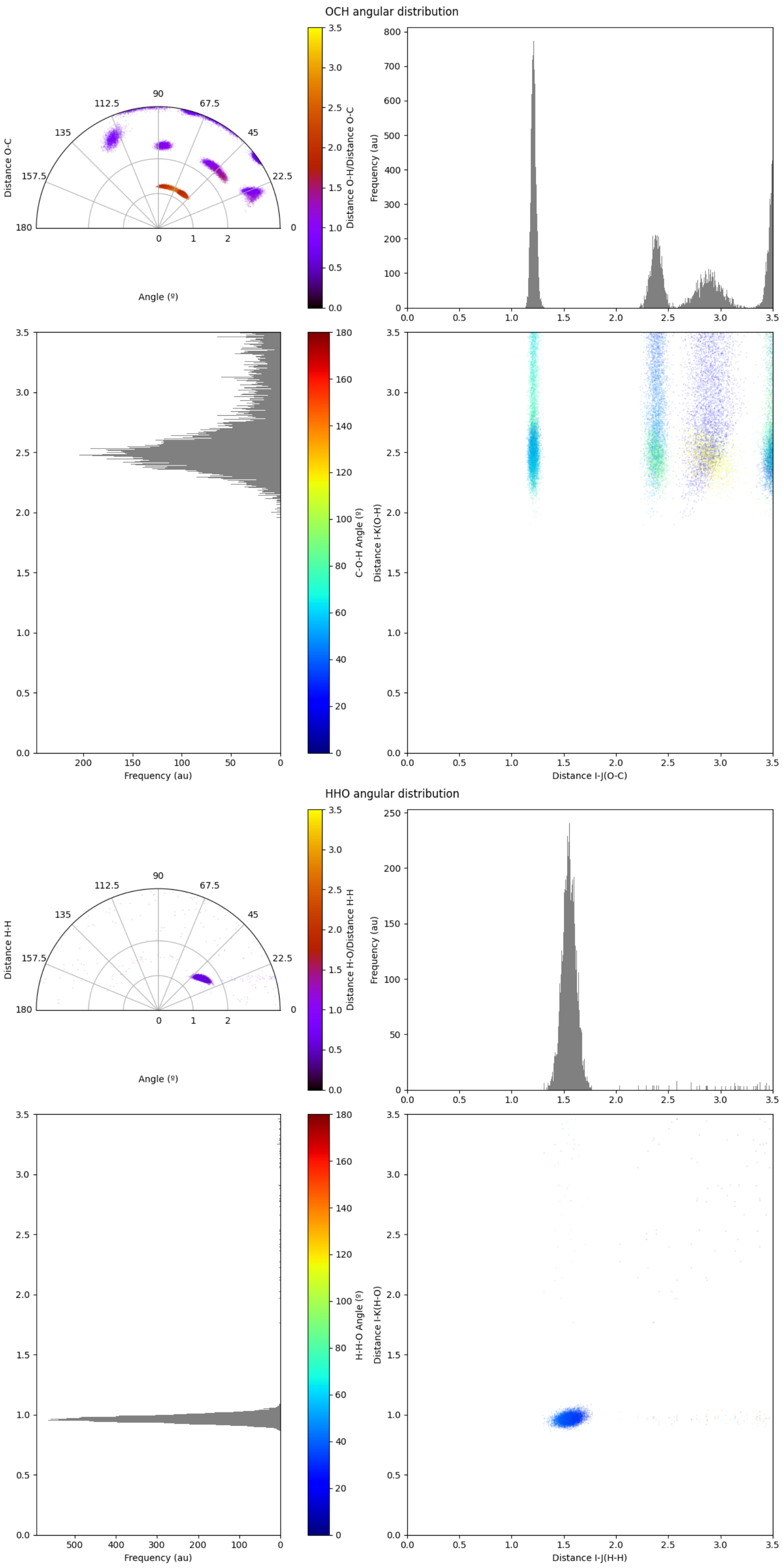

Figure 31: O-C-H and H-H-O angular distributions (500 K) of the thymine (top) and water dimer (bottom) molecules, using a cutoff radius of 3.5 Å, as sampled by the MD simulation.

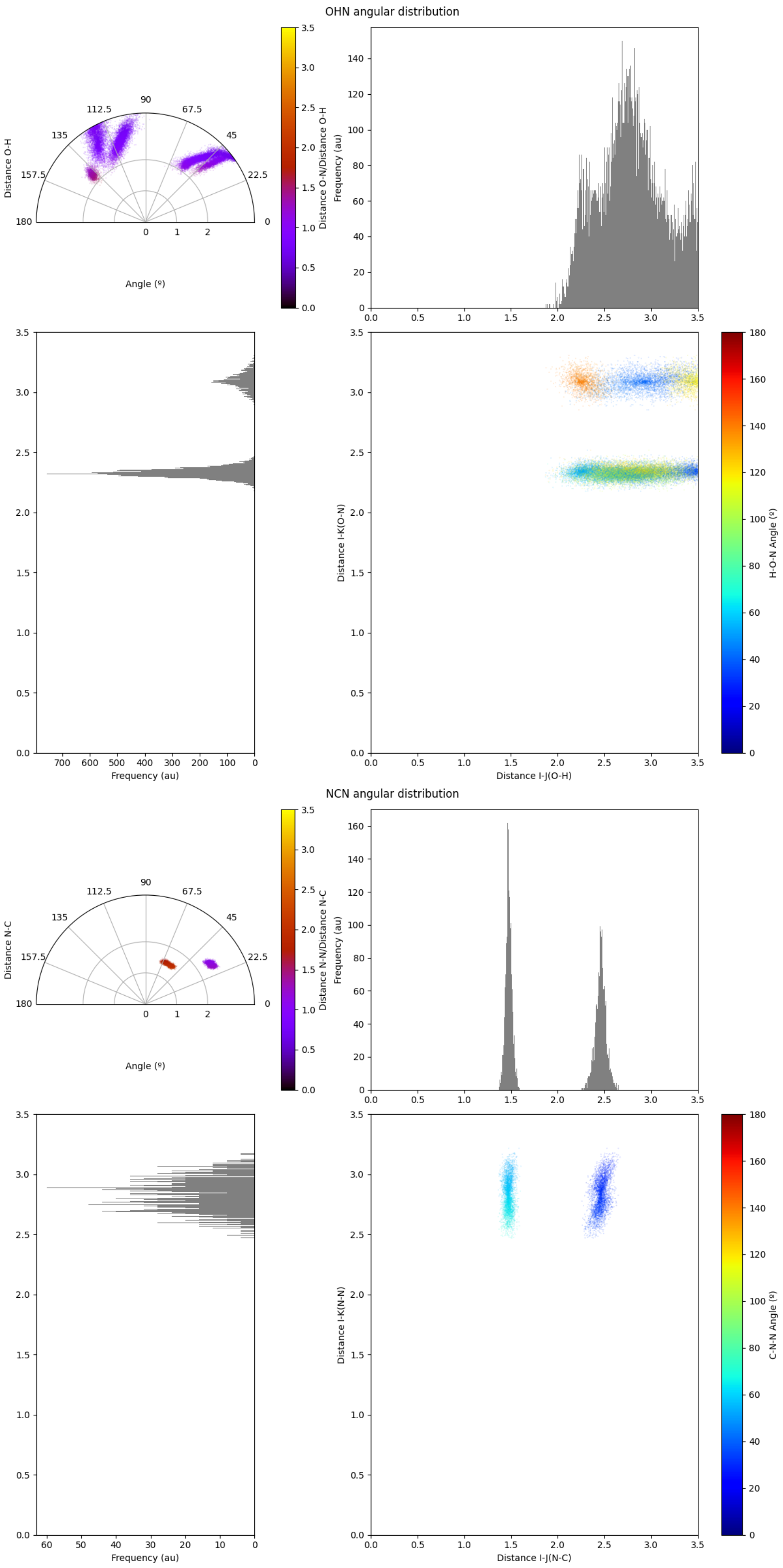

Figure 32: O-H-N and N-C-N angular distributions (500 K) of the caffeine (top) and diaminoethane (bottom) molecules, using a cutoff radius of 3.5 Å, as sampled by the NMS simulation.

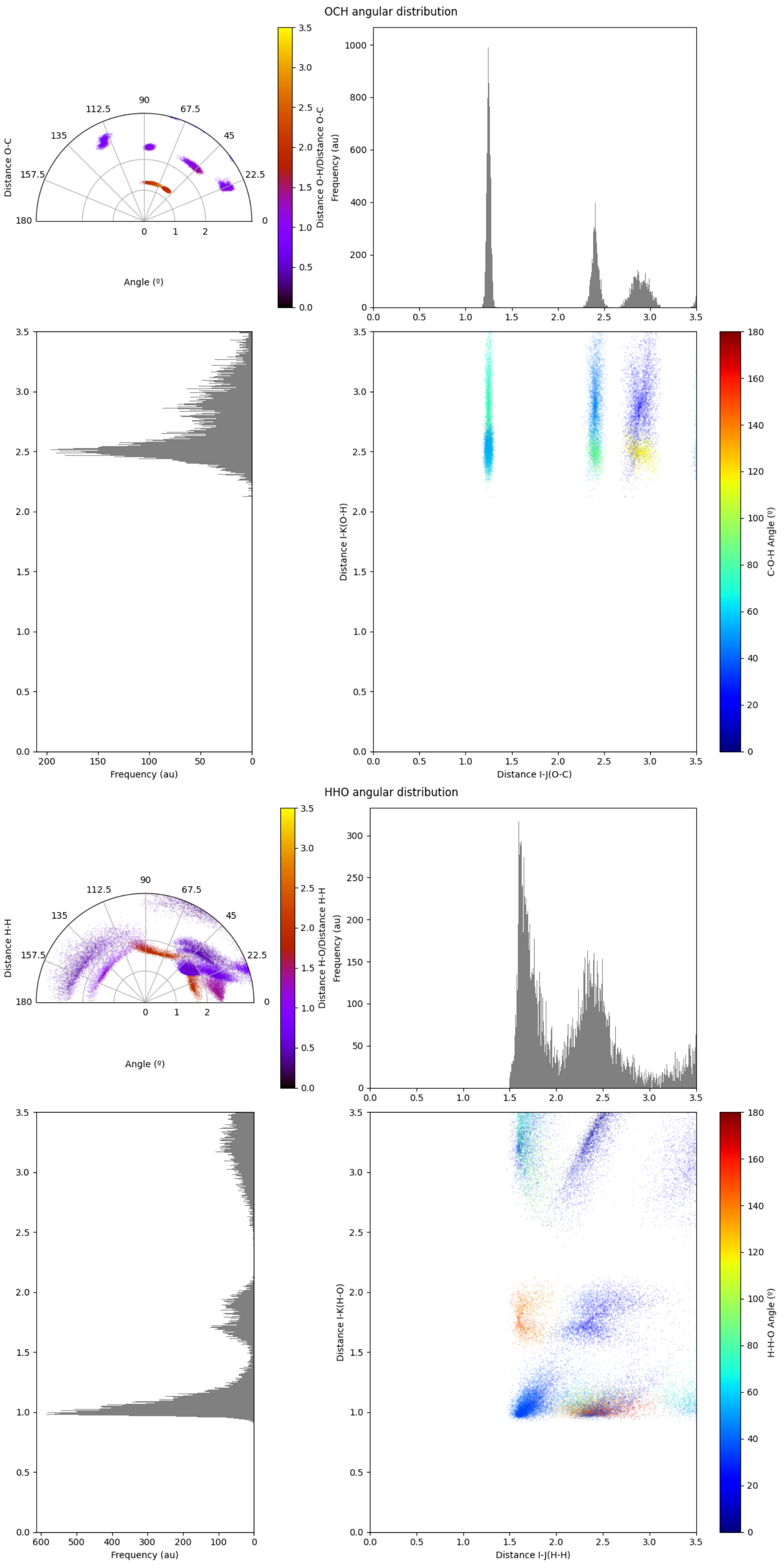

Figure 33: O-C-H and H-H-O angular distributions (500 K) of the thymine (top) and water dimer (bottom) molecules, using a cutoff radius of 3.5 Å, as sampled by the NMS simulation.

In consonance with the previously reported findings for the pairwise radial terms, both techniques appear to provide a similar sampling of the immediate angular spaces, with the exception, once again, of the particularly peculiar water dimer. However, it is crucial to be cautious when deriving strong conclusions from these and similar analyses, since both approaches may offer different conditioned chemical environments, even in spite of fairly similar isolated radial and angular distributions. As such, NMS and MD may be not be equally effective at capturing coupled phenomena, which cannot be easily represented from the distributions shown in this work.

8.3 The effect of temperature

We will now explore the effect of the temperature used to run the NMS or MD simulations on the extent with which the potential energy surface is sampled. For such a purpose, we have decided to employ two different molecules as prototypical case scenarios: the water molecule, owing to its simplicity which would ease the initial assessment of the influence of the temperature on the quality of the sampling, and 1,3,5-cyclohexanetriol. The latter represents a slightly more complex scenario, comprising a fluxional molecular backbone which displays well-defined and known conformers.

Let's start by getting a grasp of the effect of the temperature on the quality of the sampling procedure and, in turn, the resultant chemical features with the simpler scenario of the water monomer. The following figure gathers a representation of the conformational space sampled through the NMS and MD simulations of the water monomer at different temperatures, as represented by the mist-plots.

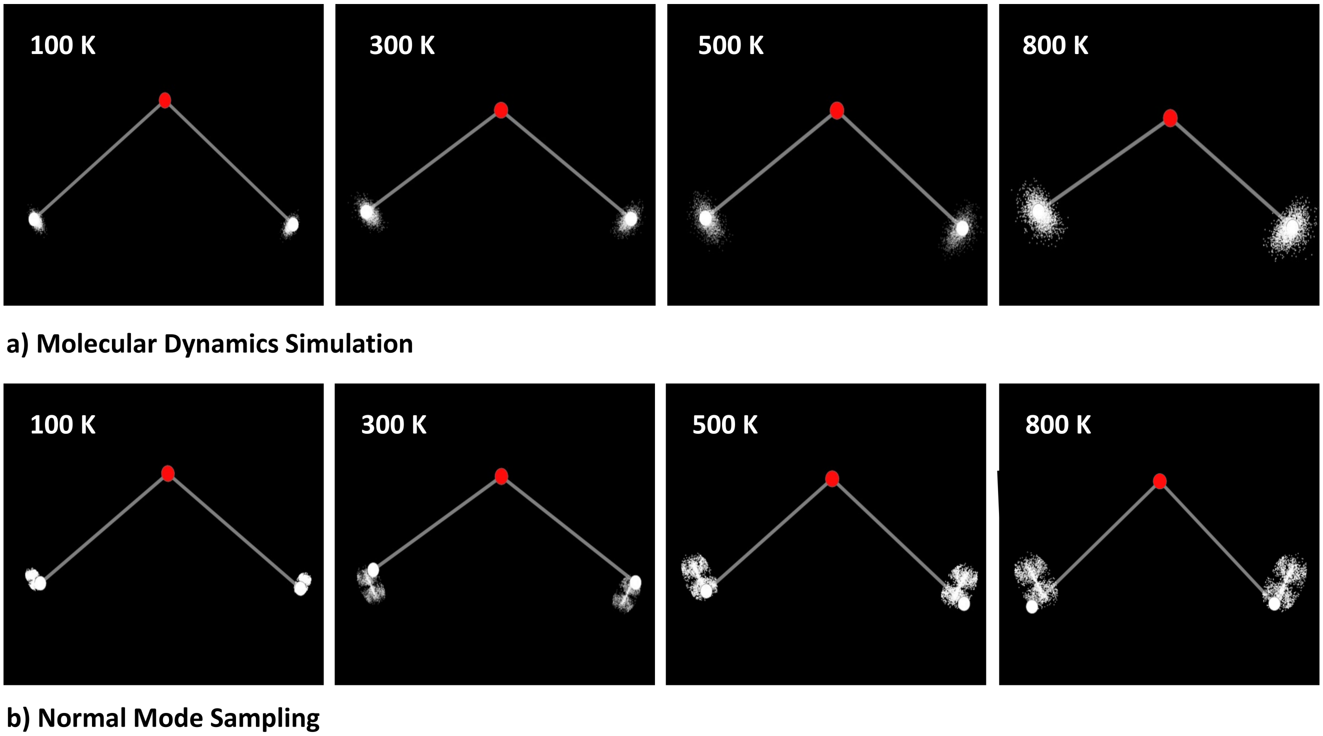

Figure 34: Mist-plots showing the conformational space sampled by the NMS and MD methodologies of the water molecule at different temperatures (100 K, 300 K, 500 K and 800 K). All the plots are centered with respect to a given reference atom. Images generated with Matplotlib[5].

Similarly, Tables 17 and 18 show the mean atomic displacements, along with their standard deviations, of the O and H atoms of the water monomer achieved by the NMS and MD sampling techniques at different temperatures. As can be seen from these tables and figures, increasing the temperature induces larger atomic displacements which, in turn, result in a more thorough exploration of the energy landscape. This is, in fact, directly evidenced from the monotonously increasing μ_{H} with the temperature, as shown in Tables 17 and 18. Consequently, the radial and angular chemical environments are expected to undergo a substantial widening with the increasing temperatures. As such, the use of moderately large temperatures is considered to be particularly beneficial as should result in more general ACSF features able to accurately describe the two- and three-body terms potentially encountered by the molecules throughout their near-equilibrium space.

Table 17: Evolution of the mean atomic displacements (μ) and standard deviations (σ) of the O and H atoms of the water molecule with the temperature employed throughout the Molecular Dynamics (MD) sampling of the PES. All values, corresponding the average for all the atoms of a given chemical element, are reported in Å. All displacements are reported relative to the O atom.

T	μ_O	μ_H	σ_O	σ_H
100	0.00	0.03	0.00	0.02
300	0.00	0.04	0.00	0.02
500	0.00	0.06	0.00	0.03
800	0.00	0.07	0.00	0.04

Table 18: Evolution of the mean atomic displacements (μ) and standard deviations (σ) of the O and H atoms of the water molecule with the temperature employed throughout the Normal Mode Sampling (NMS) of the PES. All values, corresponding the average for all the atoms of a given chemical element, are reported in Å. All displacements are reported relative to the O atom.

T	μ_O	μ_H	σ_O	σ_H
100	0.00	0.05	0.00	0.02
300	0.00	0.09	0.00	0.04
500	0.00	0.11	0.00	0.05
800	0.00	0.15	0.00	0.07

Likewise, the following figures gather the isolated O-H and H-H radial environments arising from the MD and NMS simulations of the water monomer at different temperatures. Black and red lines show the observed and reconstructed radial distributions, the latter arising from the sum of the individual clusters identified by the GMM models (shown in blue).

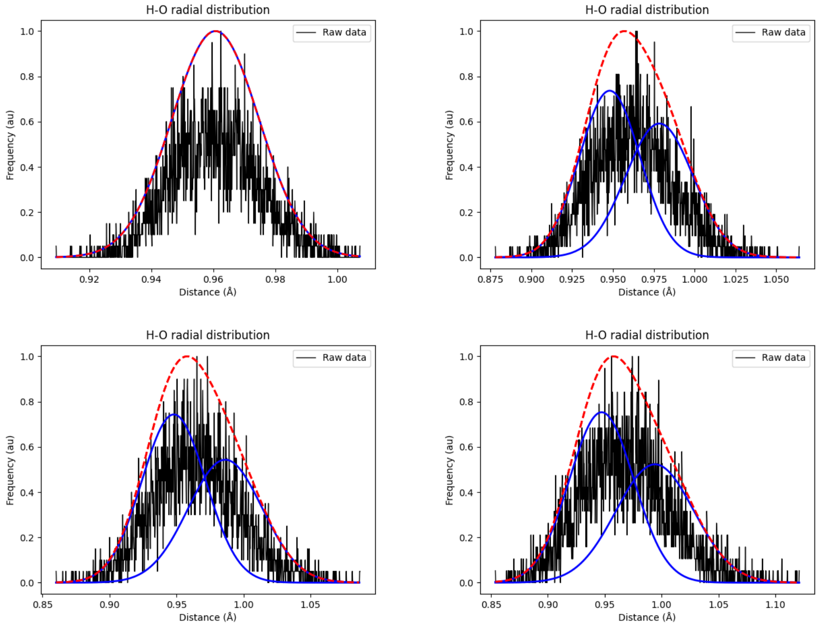

Figure 35: H-O radial distributions of the water molecule, using a cutoff radius of 7.0 Å, as sampled by the MD simulations at 100, 300, 500 and 800 K, respectively.

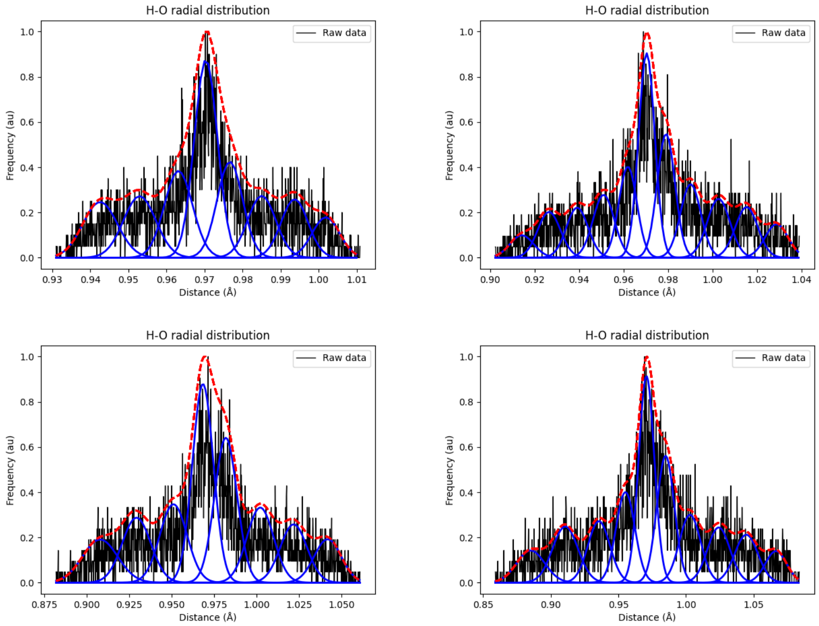

Figure 36: H-O radial distributions of the water molecule, using a cutoff radius of 7.0 Å, as sampled by the NMS simulations at 100, 300, 500 and 800 K, respectively.

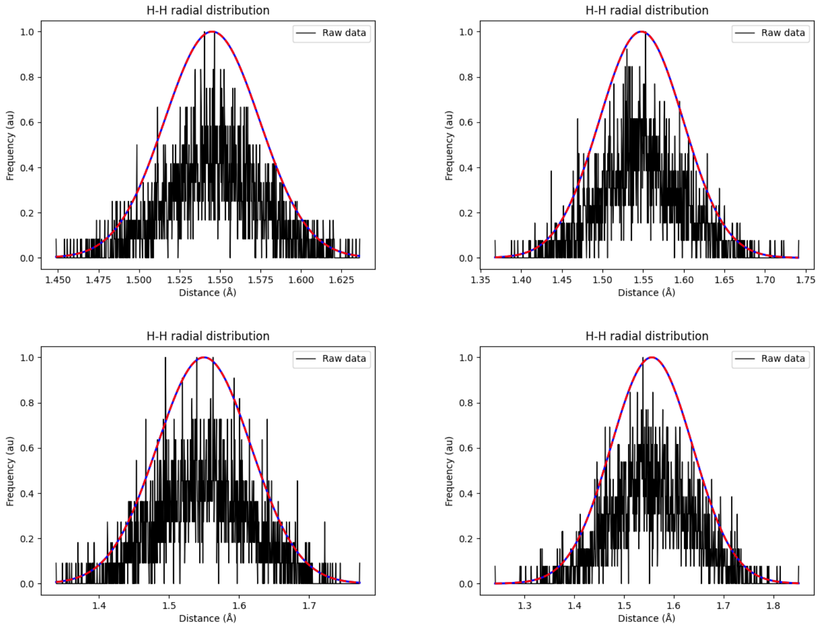

Figure 37: H-H radial distributions of the water molecule, using a cutoff radius of 7.0 Å, as sampled by the MD simulations at 100, 300, 500 and 800 K, respectively.

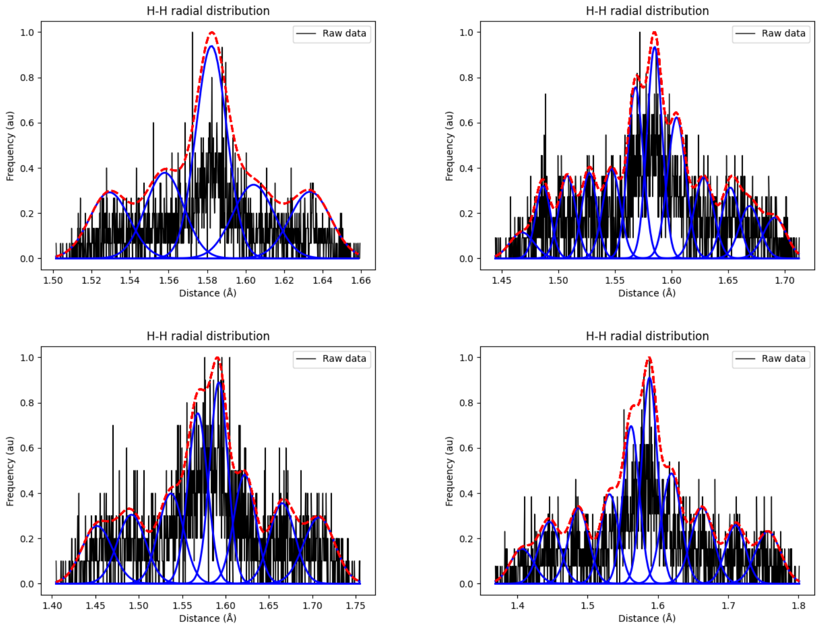

Figure 38: H-H radial distributions of the water molecule, using a cutoff radius of 7.0 Å, as sampled by the NMS simulations at 100, 300, 500 and 800 K, respectively.

First of all, it is worth mentioning that in this particular scenario, both NMS and MD simulations yield a fairly similar sampling of the radial spaces owing, mainly, to the simplicity of the system, with quite a reduced number of degrees of freedom. Moreover, and in agreement with our previous hypothesis, wider clusters emerge from the high temperature sampling of the local radial environments.

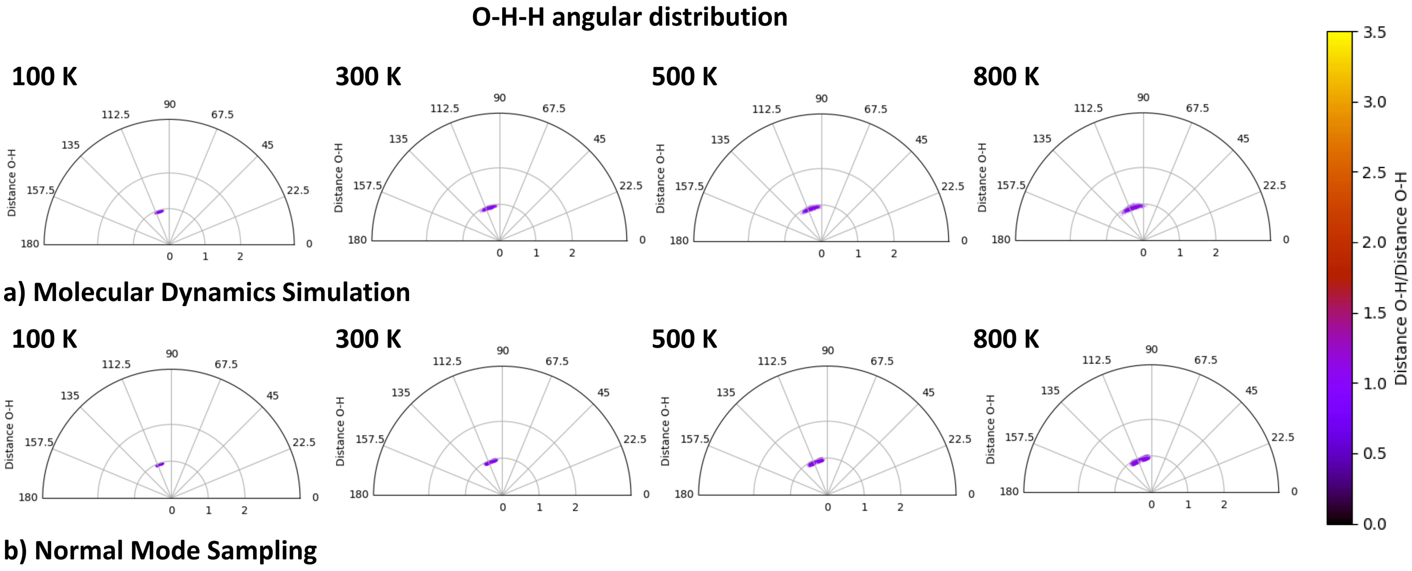

Figure 39: O-H-H angular distributions of the water molecule, using a cutoff radius of 3.5 Å, as sampled by the MD and NMS simulations at 100, 300, 500 and 800 K, respectively.

Actually, this effect becomes even more notorious from the sampling of the angular domains, as collected in Fig. 39, which clearly shows how a wider range of O-H-H angles is covered as the temperature used in the simulation is raised. On the contrary, a much more subtle effect is observed in the actual O-H and H-H bond distances, as indicated by the nearly monotonic ratio between these two values found across all the panels in the previous figure. Finally, both MD and NMS approaches afford an equivalent sampling of the angular space.

Let's now delve into the slightly more complex case of the 1,3,5-cyclohexanetriol molecule. The following figure gathers a representation of the conformational space sampled through the NMS and MD simulations of the 1,3,5-cyclohexanetriol monomer at different temperatures, as represented by the mist-plots.

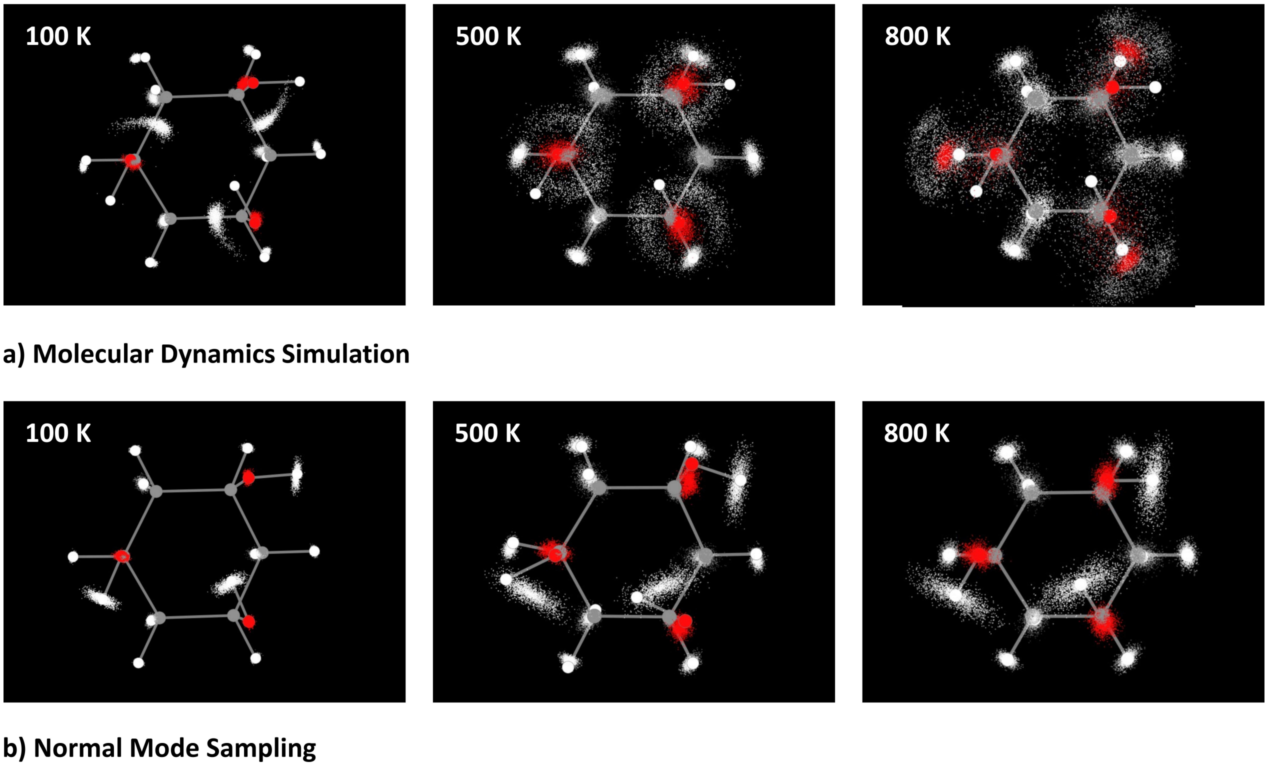

Figure 40: Mist-plots showing the conformational space sampled by the NMS and MD methodologies of the 1,3,5-cyclohexanetriol molecule at different temperatures (100 K, 500 K and 800 K). All the plots are centered with respect to a given reference atom. Images generated with Matplotlib[5].

Similarly, the following tables comprise the mean atomic displacements found throughout the NMS and MD simulations of 1,3,5-cyclohexanetriol molecule at different temperatures.

Table 19: Evolution of the mean atomic displacements (μ) and standard deviations (σ) of the C, H and O atoms of the 1,3,5-cyclohexanetriol molecule with the temperature employed throughout the Molecular Dynamics (MD) sampling of the PES. All values, corresponding the average for all the atoms of a given chemical element, are reported in Å.

T	μ_{C}	μ_{H}	μ_{O}	σ_{C}	σ_{H}	σ_{O}
100	0.10	0.44	0.15	0.03	0.08	0.05
500	0.14	0.48	0.27	0.06	0.20	0.13
800	0.47	1.36	1.66	0.27	0.62	0.83

Table 20: Evolution of the mean atomic displacements (μ) and standard deviations (σ) of the C, H and O atoms of the 1,3,5-cyclohexanetriol molecule with the temperature employed throughout the Normal Mode Sampling (NMS) of the PES. All values, corresponding the average for all the atoms of a given chemical element, are reported in Å.

T	μ_{C}	μ_{H}	μ_{O}	σ_{C}	σ_{H}	σ_{O}
100	0.03	0.08	0.07	0.02	0.05	0.04
500	0.10	0.30	0.26	0.05	0.13	0.10
800	0.17	0.43	0.27	0.06	0.16	0.12

As can be seen, considerably more interesting findings are observed in the slightly more complex case of 1,3,5-cyclohexanetriol. In agreement with the aforementioned trends, at low temperatures, the main C-C backbone of the molecule is equivalently sampled by both NMS and MD simulations, however MD provides a considerably more exhaustive sampling of the local environments of the OH and H substituents. As the temperature is increased, MD simulations are able to start sampling the different rotational degrees of freedom of the OH groups, unlike NMS which is bound to oscillate around the starting equilibrium geometry. Notably, at very large temperatures, the available thermal energy is large enough to overcome certain energetic barriers resulting in a dramatic shift in the 1,3,5-cyclohexanetriol conformation. This effect, corresponding to the transition between the two chair conformations of the cyclohexane scaffold, is only captured by the MD simulations. In fact, this can be readily seen from the top panel of the previous figure, which shows how the O atoms oscillate between two well-defined positions, attributed to both energy minima of the PES. Contrarily, the NMS trajectory keeps sampling the immediate vicinity to the starting species.

This transition between both energy minima is clearly reflected in the radial and angular distributions sampled throughout the simulations, gathered in the following figures. This becomes particularly notorious in the O-O radial distribution which shows how at 800 K a new cluster centered at about 4.5 Å , corresponding to the transition from axial to equatorial positions in the cyclohexane structure, emerges for the MD simulation.

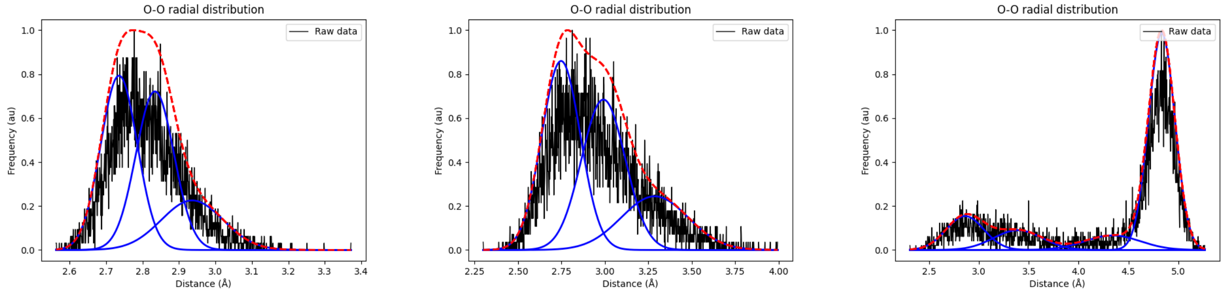

Figure 41: O-O radial distributions of the 1,3,5-cyclohexanetriol molecule, using a cutoff radius of 7.0 Å, as sampled by the MD simulations at 100, 500 and 800 K, respectively.

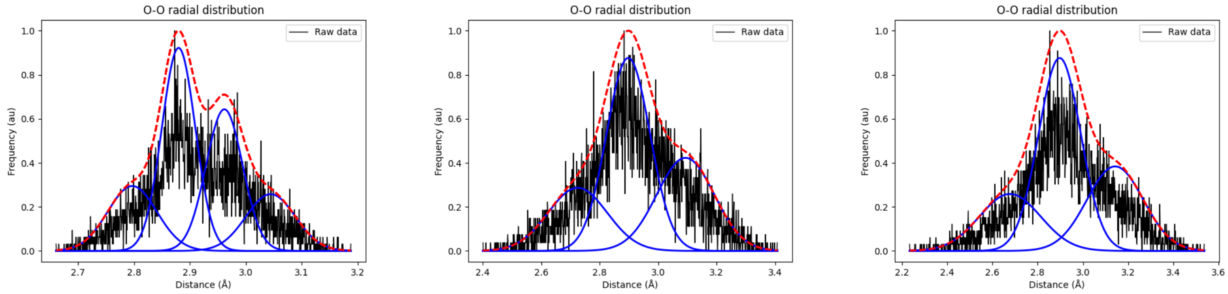

Figure 42: O-O radial distributions of the 1,3,5-cyclohexanetriol molecule, using a cutoff radius of 7.0 Å, as sampled by the NMS simulations at 100, 500 and 800 K, respectively.

Likewise, a specially dramatic change in the C-C-O angular distribution, comprised in Fig. 45 is found at 800 K, beyond which the MD simulations start to sample previously unexplored domains of the space. Interestingly enough, up to that point, both MD and NMS seem to sample relative similar distributions of the angular domains, resulting in similarly dispersed clusters at 300 K and 500 K.

In conclusion, the choice between Normal Mode Sampling (NMS) and Molecular Dynamics (MD) for sampling potential energy surfaces (PES) depends on the specific characteristics of the molecular system under investigation. NMS appears to be more suitable for intermolecular systems, where MD simulations may lead to unbinding issues. However, it is important to note that NMS often requires higher temperatures to achieve an equivalent sampling of the configuration space. On the other hand, MD excels in exploring various regions of the PES beyond the starting equilibrium well, making it particularly useful for investigating conformational changes involving rotations

and bending in flexible scaffolds. Notably, MD allows the exploration of diverse equilibria, whereas NMS is confined to the initial equilibrium geometry. To achieve comparable sampling, NMS may necessitate running multiple simulations from different equilibrium geometries. Ultimately, the decision between NMS and MD should be based on the specific characteristics and objectives of the molecular system being studied.

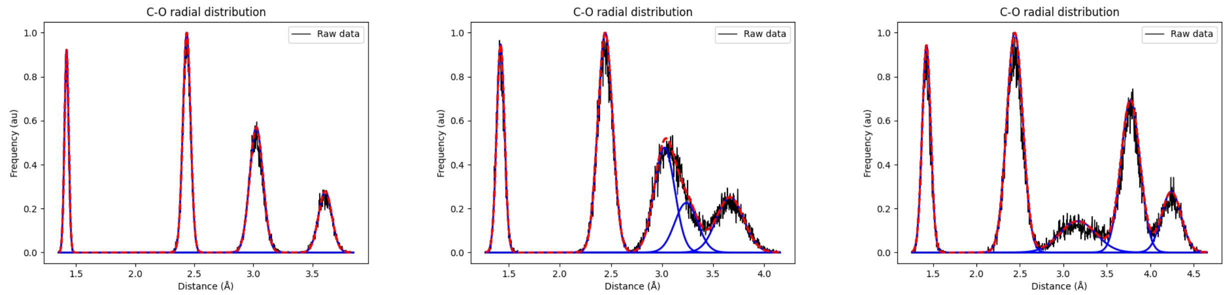

Figure 43: C-O radial distributions of the 1,3,5-cyclohexanetriol molecule, using a cutoff radius of 7.0 Å, as sampled by the MD simulations at 100, 500 and 800 K, respectively.

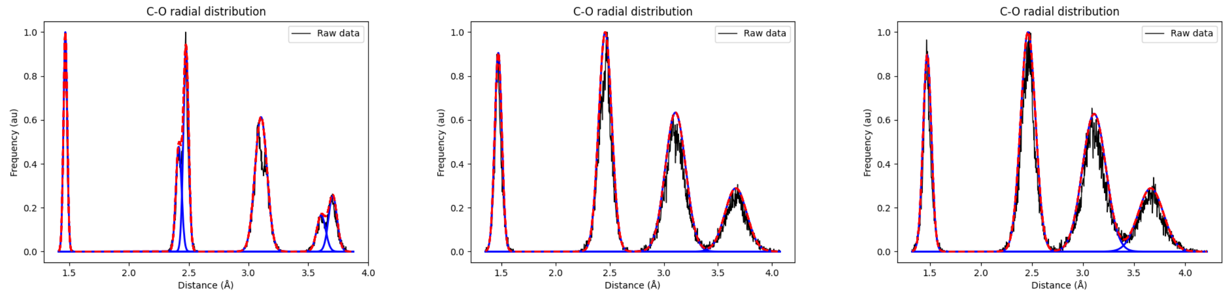

Figure 44: C-O radial distributions of the 1,3,5-cyclohexanetriol molecule, using a cutoff radius of 7.0 Å, as sampled by the NMS simulations at 100, 500 and 800 K, respectively.

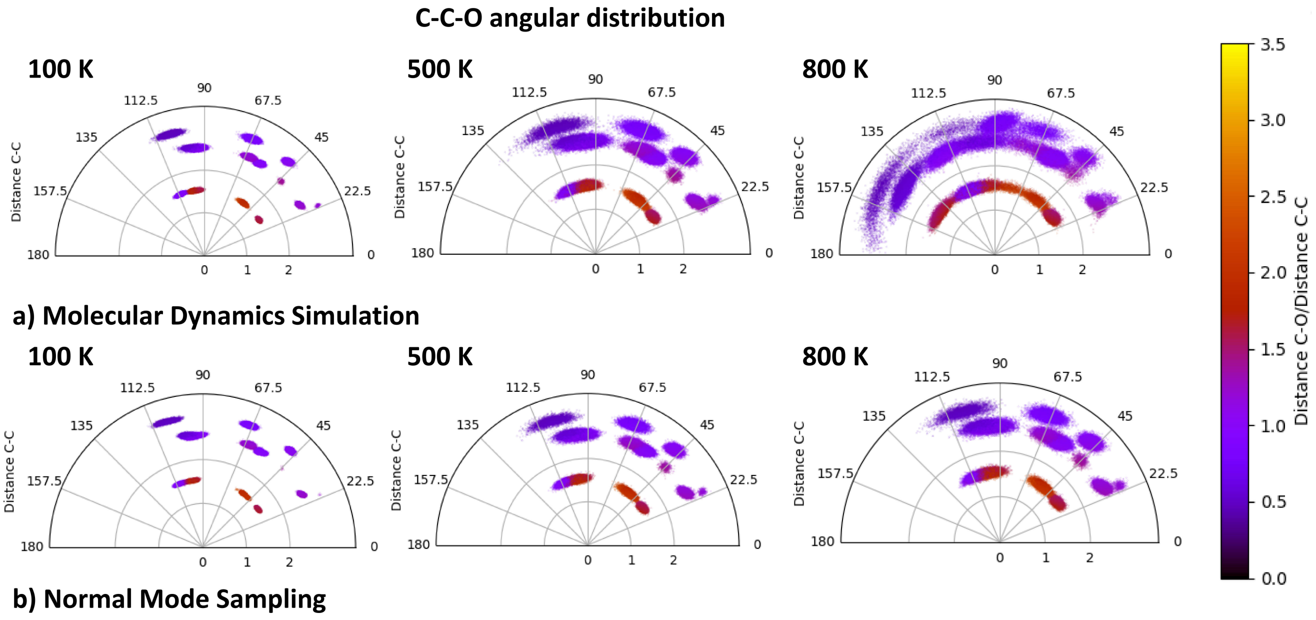

Figure 45: C-C-O angular distributions of the 1,3,5-cyclohexanetriol molecule, using a cutoff radius of 3.5 Å, as sampled by the MD and NMS simulations at 100, 500 and 800 K, respectively.

9 Identifying radial and angular environments in diverse chemical spaces

Finally, we have also decided to test the reliability and applicability of the here presented approach when presented with heterogeneous data, coming from the exploration of the potential energy landscape of different molecules. Despite this is not the main aim of our work, if successful, this would show the potential validity of our approximation to explore not only the conformational space of a system but the considerably more intricate chemical space. For such a purpose we have decided to rely on the NNAIMQ database, introduced some years ago by some of us.[15] The latter gathers a large collection (about 46 000 geometries) of heterogeneous C, H, O and N containing molecules sampled from the gas-phase near-equilibrium CHON chemical space. As such, it comprises an ideal scenario to test our approach.

Given the considerable size of the NNAIMQ database, along with the large computational cost involved in the exploration of the angular spaces, we have decided to run the GMM clustering technique on a tiny fraction of the whole database. The latter, randomly sampled from the whole pool, gathers a total of 3865 CHON molecules. Unless otherwise specified, the exploration of the radial and angular domains was performed on every frame (`trj_step=1`). The maximum number of radial and angular cluster was set to 15 and 20, respectively. For the exploration of the angular space the top 75% of the functions were selected out from the ideal number of components, found through the `gmm_crit="bicconv"` approach using `percbic=30, percdiff=40`.

9.1 GMM exploration of the CHON chemical space

Let's start by exploring the ability of our approach to decompose the radial and and angular spaces belonging to different chemical spaces. For the radial environments, the tailor-made spatial distribution, without further decompositions or auxiliary functions, was employed with a cutoff radius of 7.0 Å. On the other hand, the latter was reduced to a more moderate value of 3.5 Å for the angular environments.

Radial Environments

The following figures show the identified clusters within the radial environments of the C atoms in the explored CHON space. Black and red lines show the observed and reconstructed radial distributions, the latter arising from the sum of the individual clusters identified by the GMM models (shown in blue).

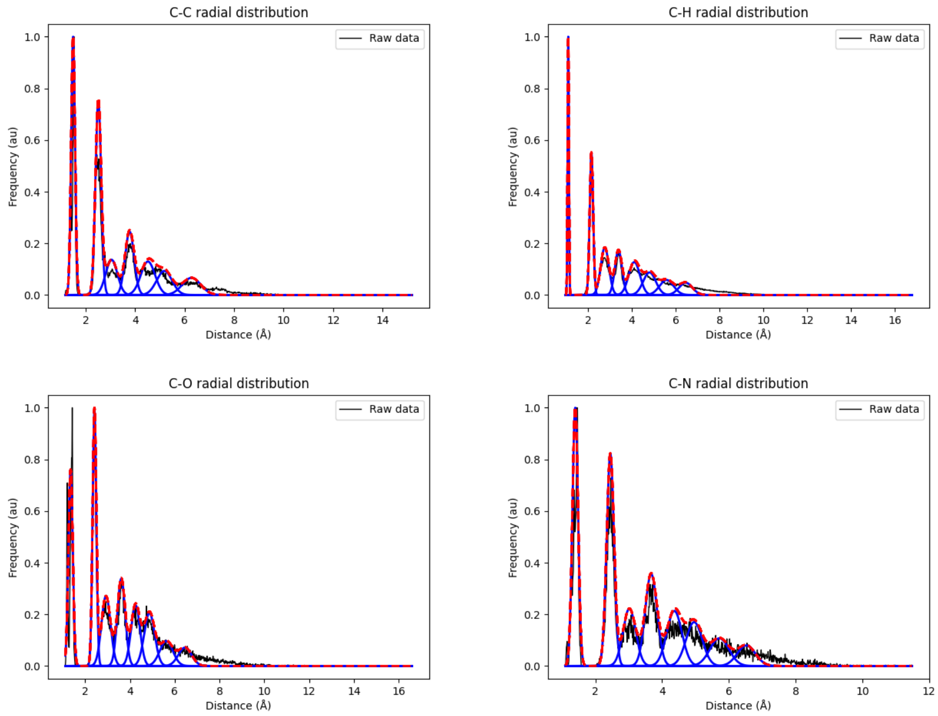

Figure 46: Radial environments of the C atoms in the CHON subset, using a cutoff radius of 7.0 Å.

The following figures show the identified clusters within the radial environments of the H atoms in the explored CHON space. Black and red lines show the observed and reconstructed radial distributions, the latter arising from the sum of the individual clusters identified by the GMM models (shown in blue).

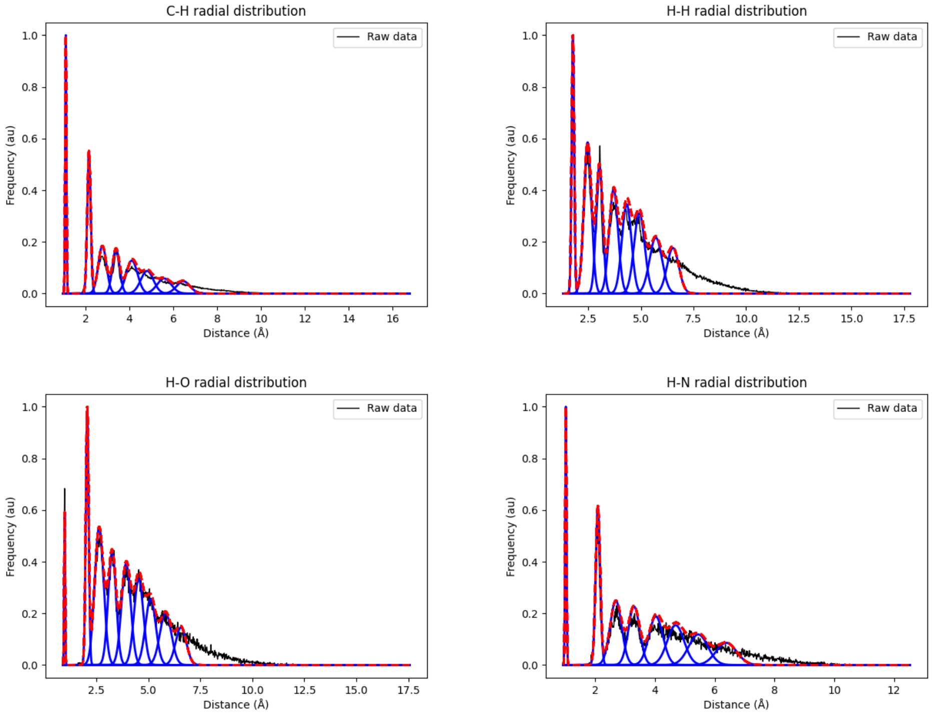

Figure 47: Radial environments of the H atoms in the CHON subset, using a cutoff radius of 7.0 Å.

The following figures show the identified clusters within the radial environments of the O atoms in the explored CHON space. Black and red lines show the observed and reconstructed radial distributions, the latter arising from the sum of the individual clusters identified by the GMM models (shown in blue).

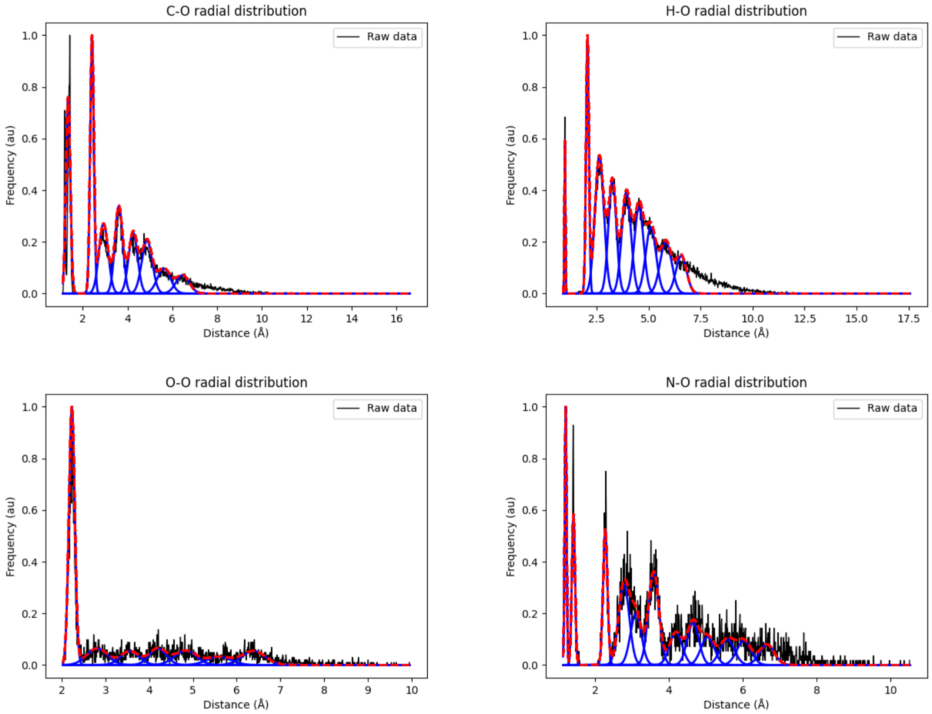

Figure 48: Radial environments of the O atoms in the CHON subset, using a cutoff radius of 7.0 Å.

The following figures show the identified clusters within the radial environments of the N atoms in the explored CHON space. Black and red lines show the observed and reconstructed radial distributions, the latter arising from the sum of the individual clusters identified by the GMM models (shown in blue).

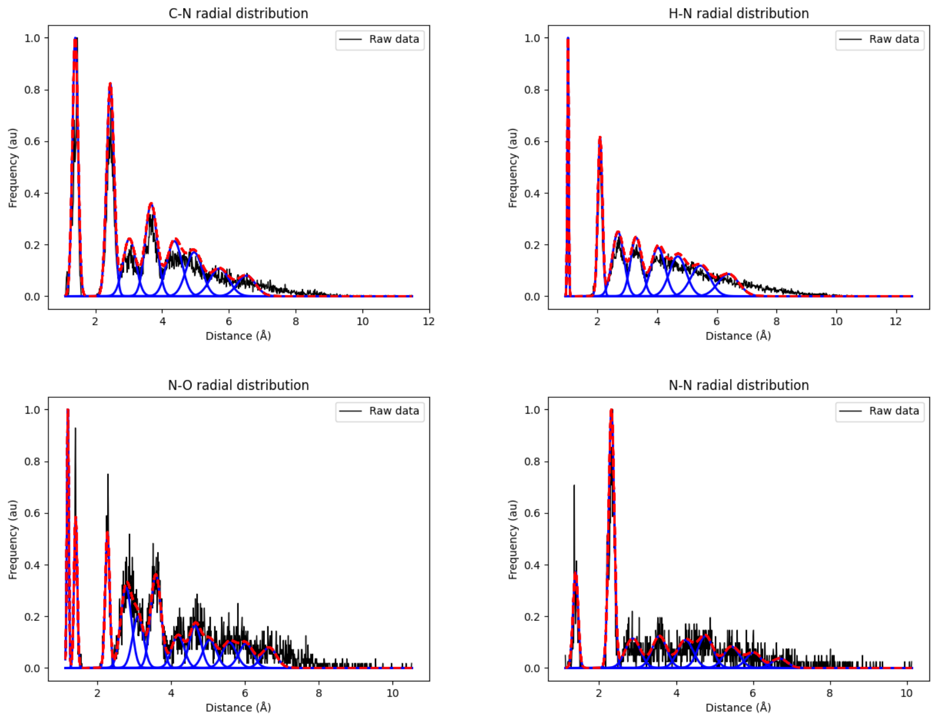

Figure 49: Radial environments of the N atoms in the CHON subset, using a cutoff radius of 7.0 Å.

As can be seen from the previous figures, our approach is perfectly able to explore the radial environments of a heterogeneous chemical space, resulting in a clear-cut decomposition even when arising from different potential energy surfaces. Additionally, a notably distinctive feature, typically absent when exploring the conformational space of a single molecule distribution as demonstrated earlier, emerges. To be more specific, while short distances reveal well-defined and fairly localized clusters in the radial distribution, a progressively decreasing and less defined cluster is commonly observed at longer distances. This finding can be intuitively explained: most CHON organic scaffolds consist of a plethora of common functional groups which are frequently found through the chemical space. This effectively imposes certain constraints in the diversity of the immediate chemical contacts which results in a well-defined short-range radial distribution. Instead, at longer distances the diversity of chemical contacts grows rapidly which yields much less defined medium-to-long range radial distributions. Furthermore, following this rationale, one would anticipate that a higher radial resolution proves particularly beneficial at short range, whereas lower accuracies would generally suffice to describe chemical contacts in the far range. Interestingly enough, this can be readily seen in the self-tuned radial features which tend to become wider with the distance to the reference atomic center.

Angular Environments

The following figures show the identified clusters within the angular environments of the C atoms in the explored CHON space.

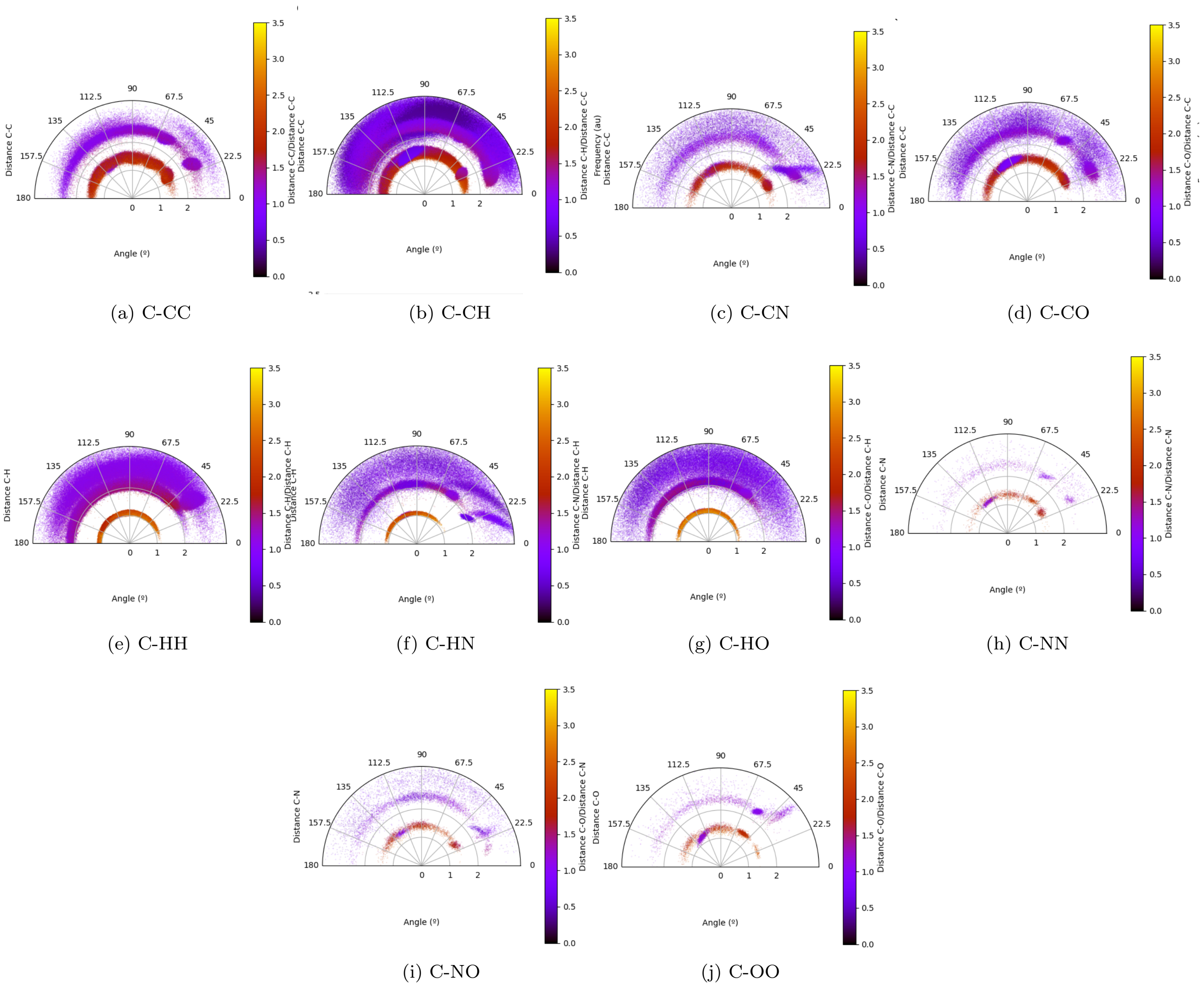

(a) C-CC (b) C-CH (c) C-CN (d) C-CO

(e) C-HH (f) C-HN (g) C-HO (h) C-NN

(i) C-NO (j) C-OO

Figure 50: Angular environments of the C atoms in the CHON subset, using a cutoff radius of 3.5 Å.

The following figures show the identified clusters within the angular environments of the H atoms in the explored CHON space.

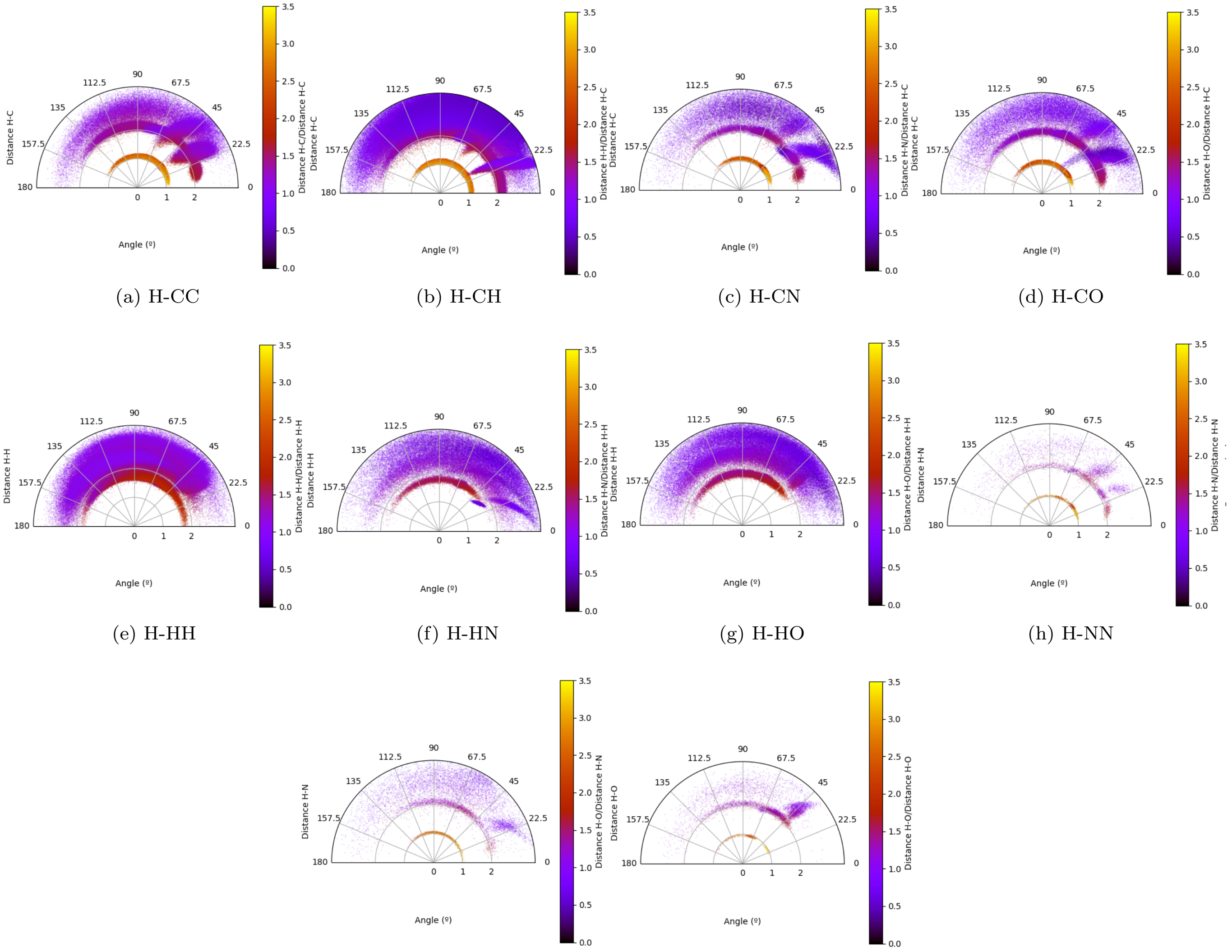

(a) H-CC (b) H-CH (c) H-CN (d) H-CO

(e) H-HH (f) H-HN (g) H-HO (h) H-NN

(i) H-NO (j) H-OO

Figure 51: Angular environments of the H atoms in the CHON subset, using a cutoff radius of 3.5 Å.

The following figures show the identified clusters within the angular environments of the O atoms in the explored CHON space.

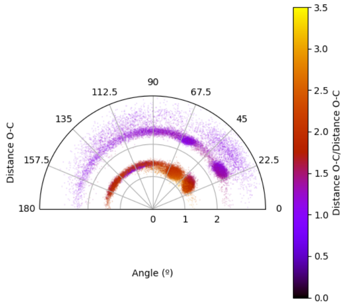

(a) O-CC

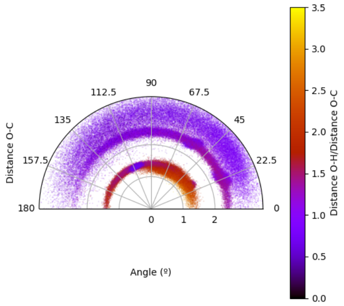

(b) O-CH

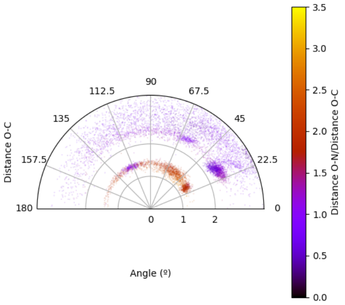

(c) O-CN

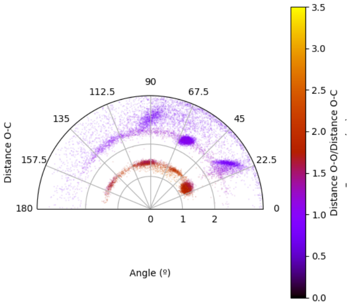

(d) O-CO

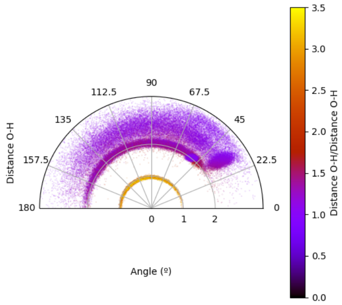

(e) O-HH

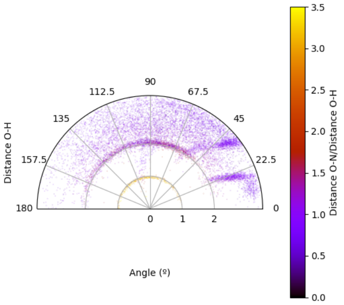

(f) O-HN

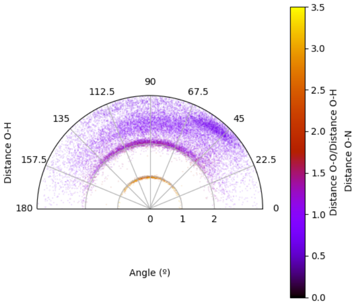

(g) O-HO

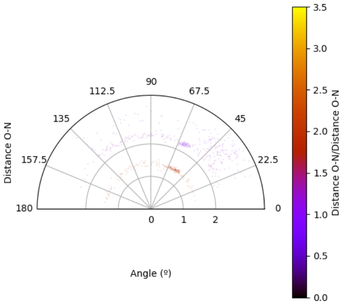

(h) O-NN

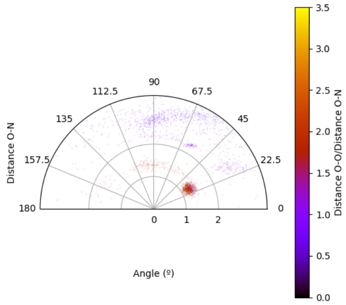

(i) O-NO

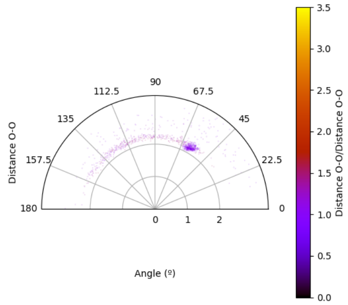

(j) O-OO

Figure 52: Angular environments of the O atoms in the CHON subset, using a cutoff radius of 3.5 Å.

The following figures show the identified clusters within the angular environments of the N atoms in the explored CHON space.

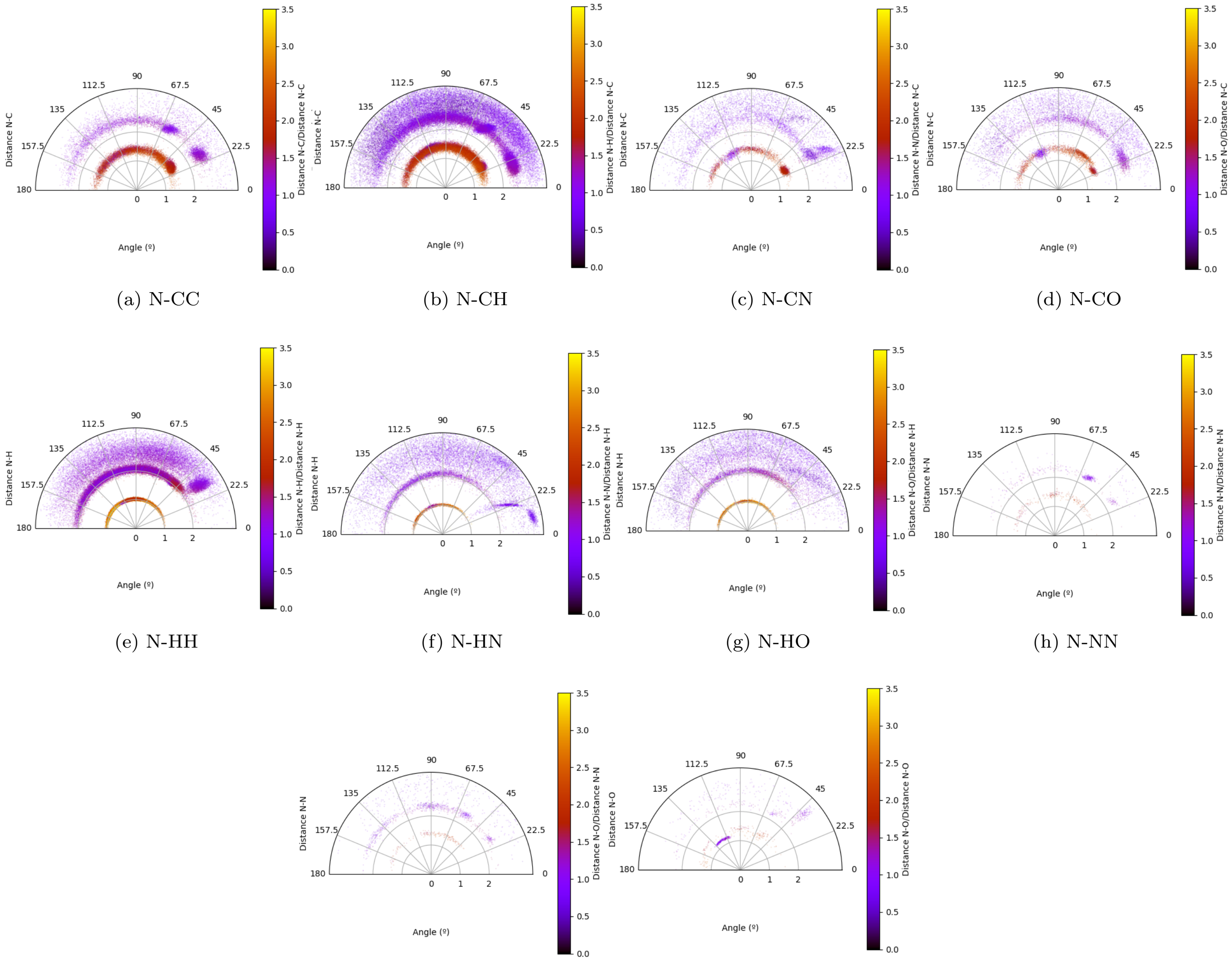

(a) N-CC (b) N-CH (c) N-CN (d) N-CO (e) N-HH (f) N-HN (g) N-HO (h) N-NN (i) N-NO (j) N-OO

Figure 53: Angular environments of the N atoms in the CHON subset, using a cutoff radius of 3.5 Å.

Analogously to the scenario found for the radial environments, our unsupervised clustering technique is capable of adequately identifying the most frequent angular environments encountered throughout the explored here CHON chemical space. Additionally, it is interesting to notice that the aforementioned blurring of the chemical environments with the distance becomes even more pronounced for the three-body interactions. In fact, the latter seem to exhibit highly dispersed distributions anywhere beyond the 2 Å threshold.

Altogether, these findings evidence the suitability of our approach to deal with heterogeneous databases, capable of simultaneously exploring different potential energy landscapes of a diverse chemical space.

9.2 Performance of the self-optimized features

After showing the ability of our approach to efficiently explore and decompose intricate chemical spaces corresponding to different molecules, we will now briefly explore the quality of the resultant features. For the sake of simplicity, we will train plain FFNN models, as discussed in previous sections, to predict the QTAIM atomic charges of the NNAIMQ database.[15] The accuracy of the resultant models will be compared to that obtained with a careful selection of hand-crafted descriptors, manually optimized in previous work.[15]

In order to achieve a thorough exploration of the radial spaces, the `binary` spatial distribution scheme was used throughout with $\alpha = 3$ and $\beta = 1$, owing to their outstanding performance found throughout the text. Similarly, the regular tailor-made approach was used to tune and distribute in space the angular ACSF functions. On the other hand, two different cutoff radii were explored, namely 7.0 and 10.0 Å, and 3.5 and 10.0 Å for the radial and angular environments, respectively. The first one (7.0 and 3.5 Å) was selected based on the thorough analysis performed throughout the manuscript. On the other hand, the considerably longer cutoff radius of 10.0 Å was used to match that employed through the manual selection of the features.[15]

Radial ACSF with a 7.0 Å cutoff

The following figures show the self-tuned radial ACSF features within the CHON chemical space using a cutoff radius of 7.0 Å. Black and red lines show the observed and reconstructed radial distributions, the latter arising from the sum of the individual clusters identified by the GMM models (shown in blue).

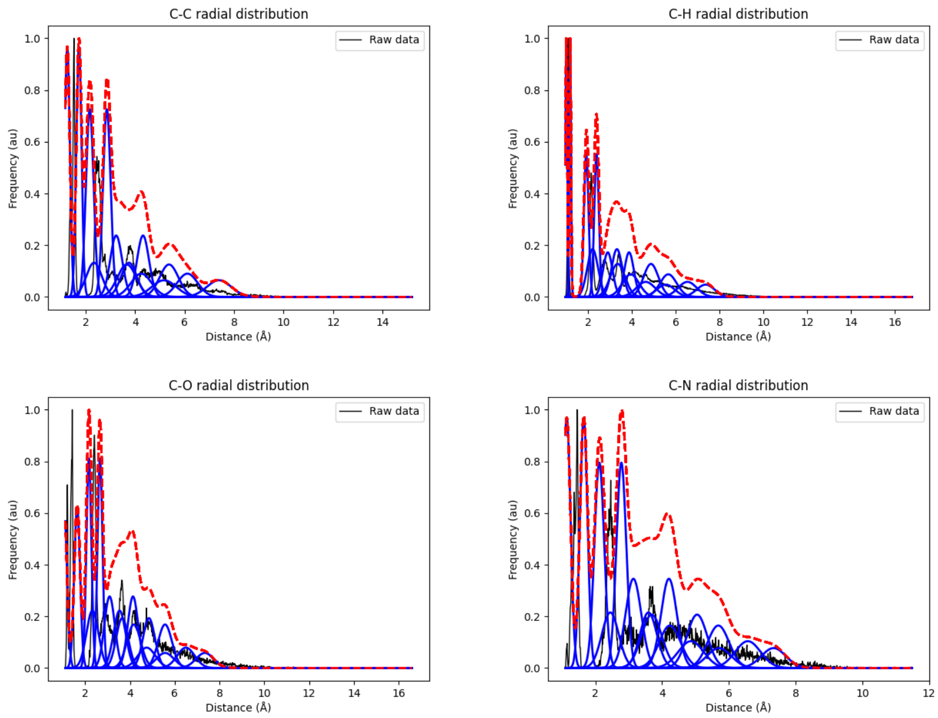

Figure 54: Optimized radial ACSF of the C atoms in the CHON subset, using a cutoff radius of 7.0 Å.

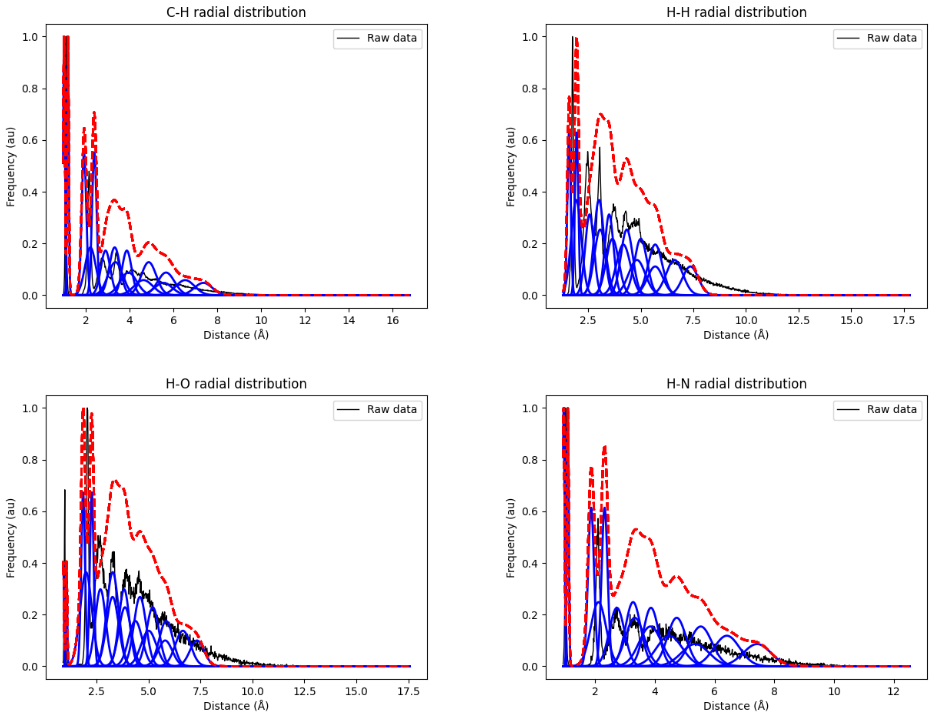

Figure 55: Optimized radial ACSF of the H atoms in the CHON subset, using a cutoff radius of 7.0 Å.

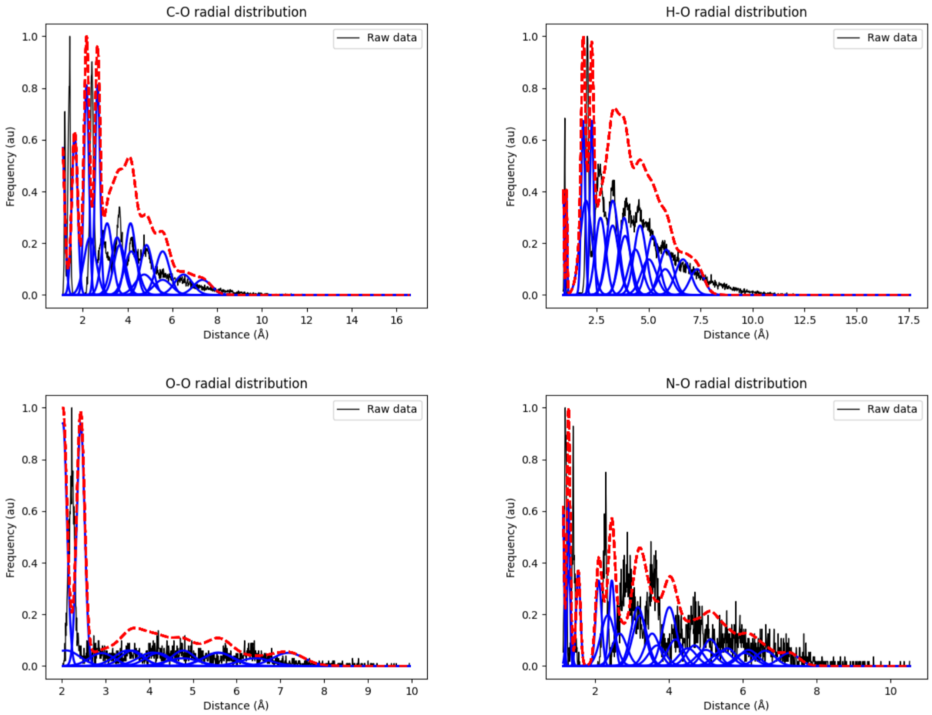

Figure 56: Optimized radial ACSF of the O atoms in the CHON subset, using a cutoff radius of 7.0 Å.

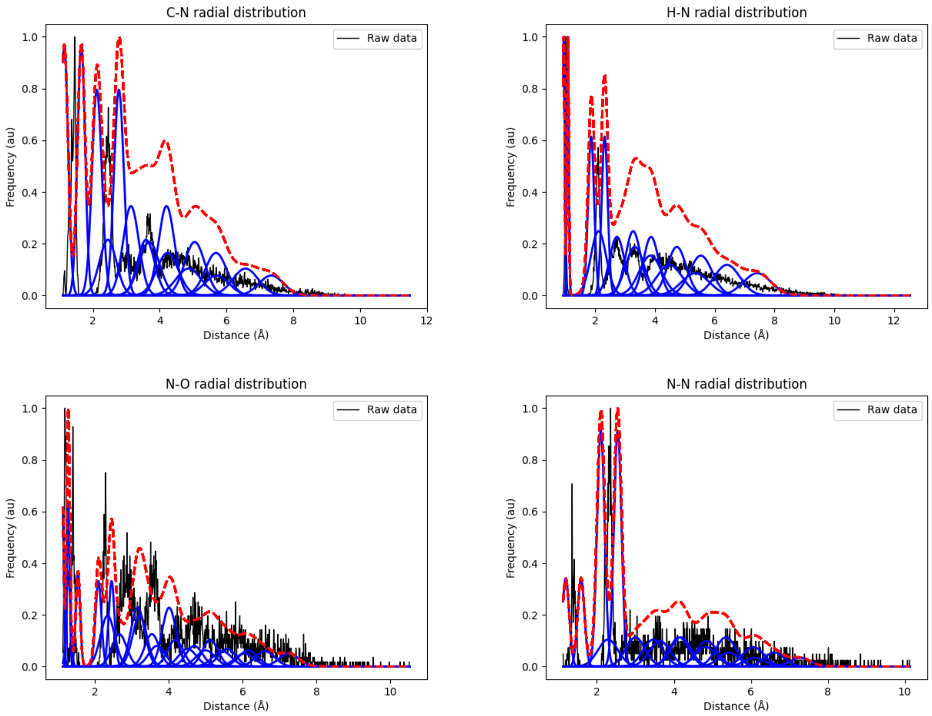

Figure 57: Optimized radial ACSF of the N atoms in the CHON subset, using a cutoff radius of 7.0 Å.

Radial ACSF with a 10.0 Å cutoff

The following figures show the self-tuned radial ACSF features within the CHON chemical space using a cutoff radius of 10.0 Å. Black and red lines show the observed and reconstructed radial distributions, the latter arising from the sum of the individual clusters identified by the GMM models (shown in blue).

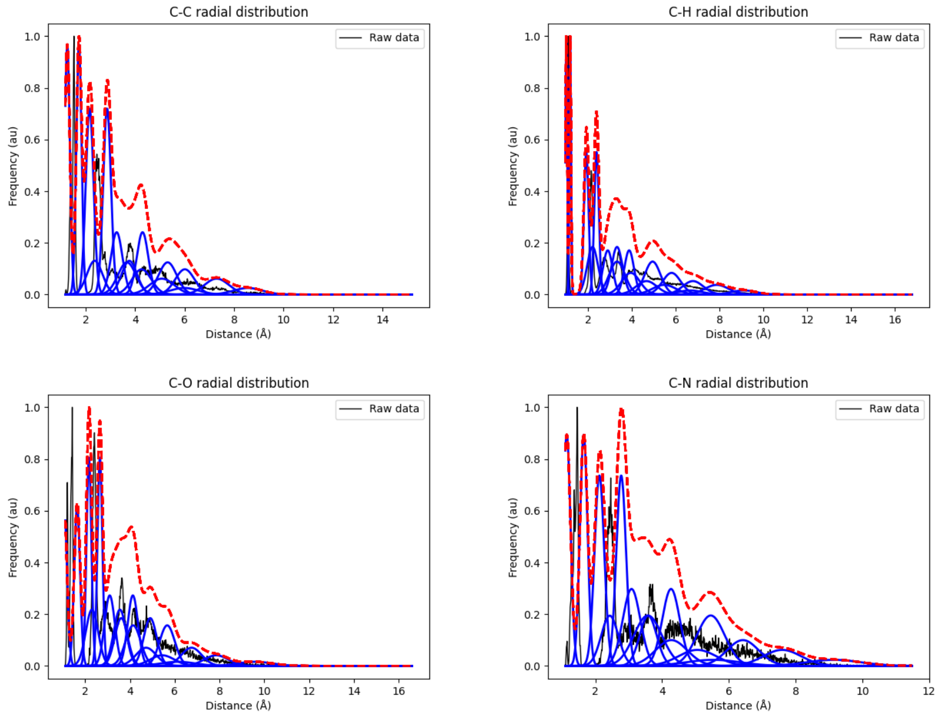

Figure 58: Optimized radial ACSF of the C atoms in the CHON subset, using a cutoff radius of 10.0 Å.

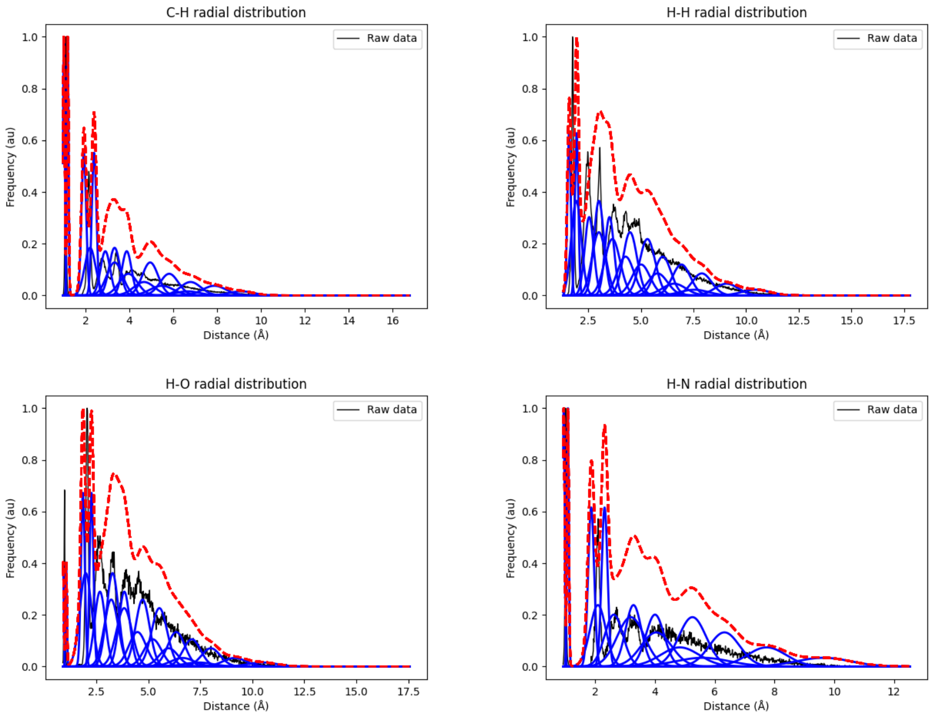

Figure 59: Optimized radial ACSF of the H atoms in the CHON subset, using a cutoff radius of 10.0 Å.

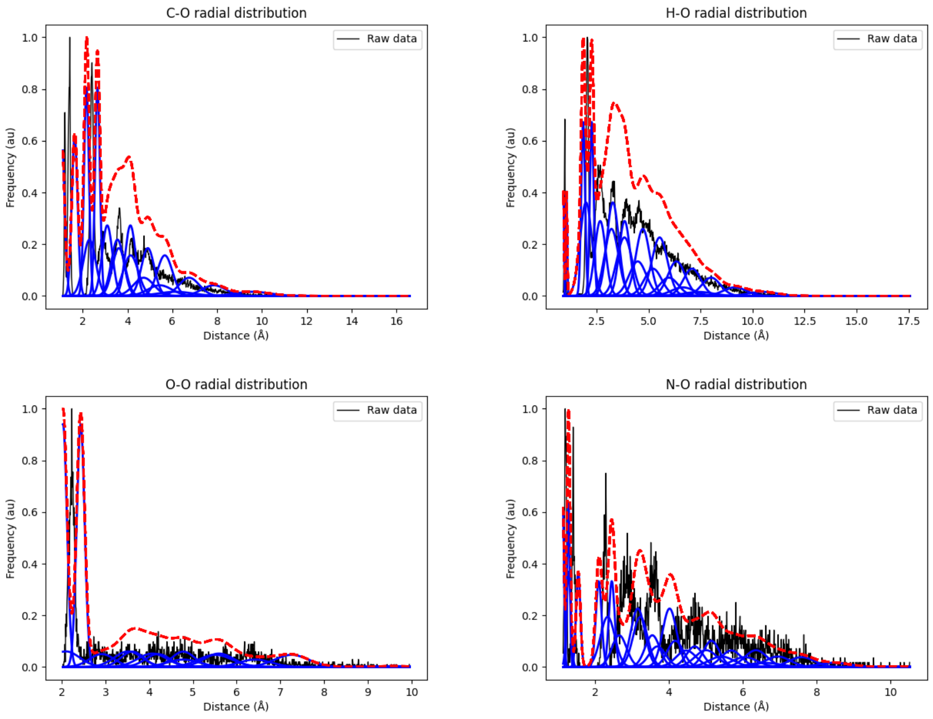

Figure 60: Optimized radial ACSF of the O atoms in the CHON subset, using a cutoff radius of 10.0 Å.

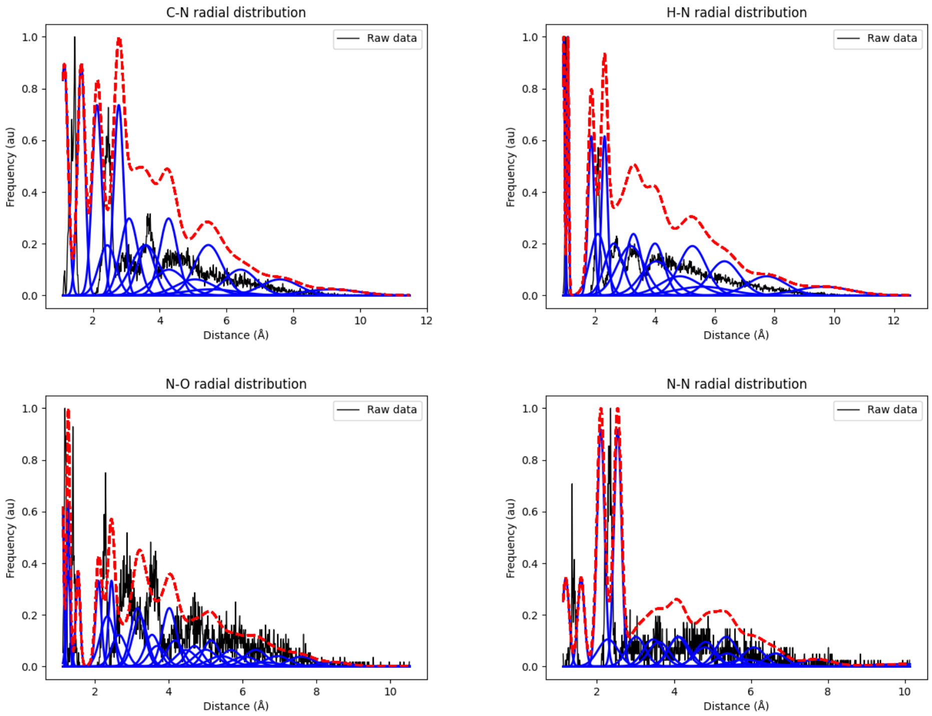

Figure 61: Optimized radial ACSF of the N atoms in the CHON subset, using a cutoff radius of 10.0 Å.

Angular ACSF with a 3.5 Å cutoff

The following figures show the self-tuned angular ACSF features within the CHON chemical space using a cutoff radius of 3.5 Å.

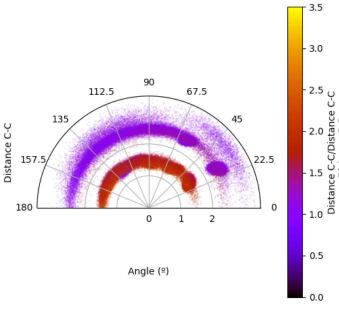

(a) C-CC

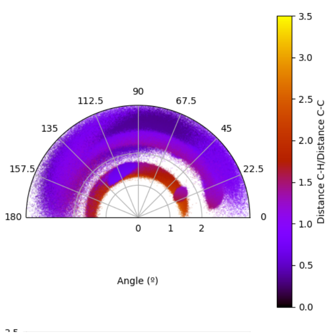

(b) C-CH

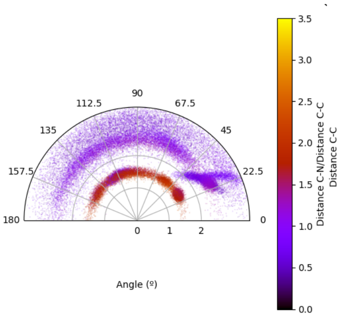

(c) C-CN

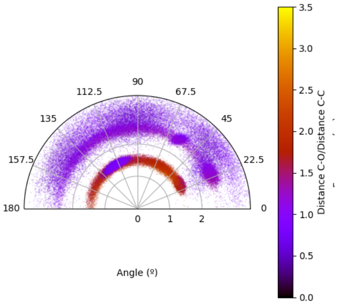

(d) C-CO

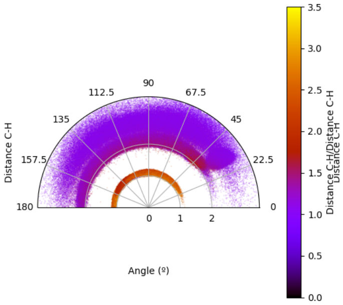

(e) C-HH

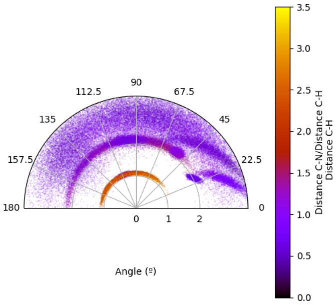

(f) C-HN

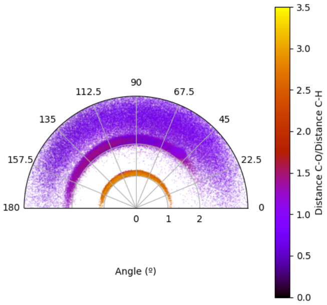

(g) C-HO

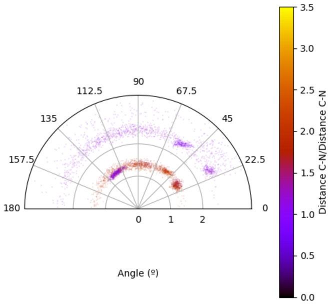

(h) C-NN

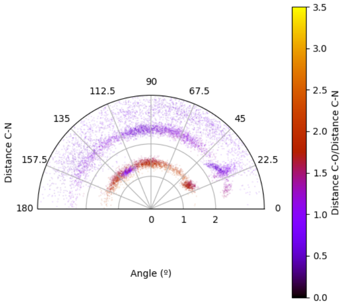

(i) C-NO

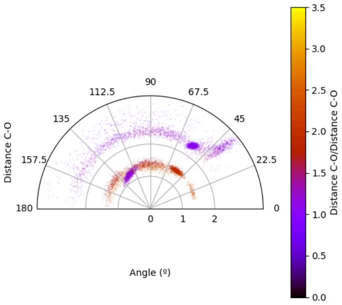

(j) C-OO

Figure 62: Optimized angular ACSF of the C atoms in the CHON subset, using a cutoff radius of 3.5 Å.

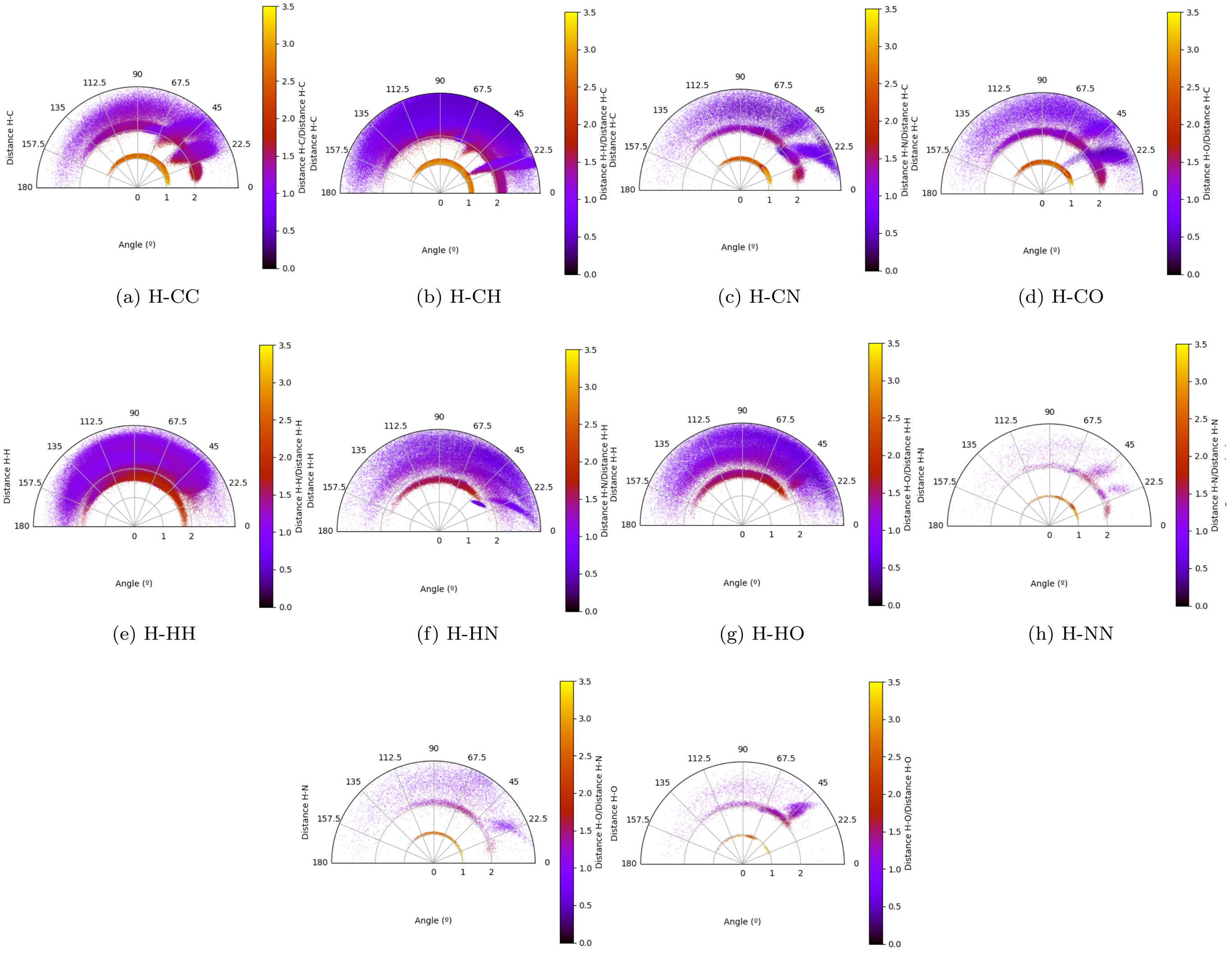

(a) H-CC (b) H-CH (c) H-CN (d) H-CO

(e) H-HH (f) H-HN (g) H-HO (h) H-NN

(i) H-NO (j) H-OO

Figure 63: Optimized angular ACSF of the H atoms in the CHON subset, using a cutoff radius of 3.5 Å.

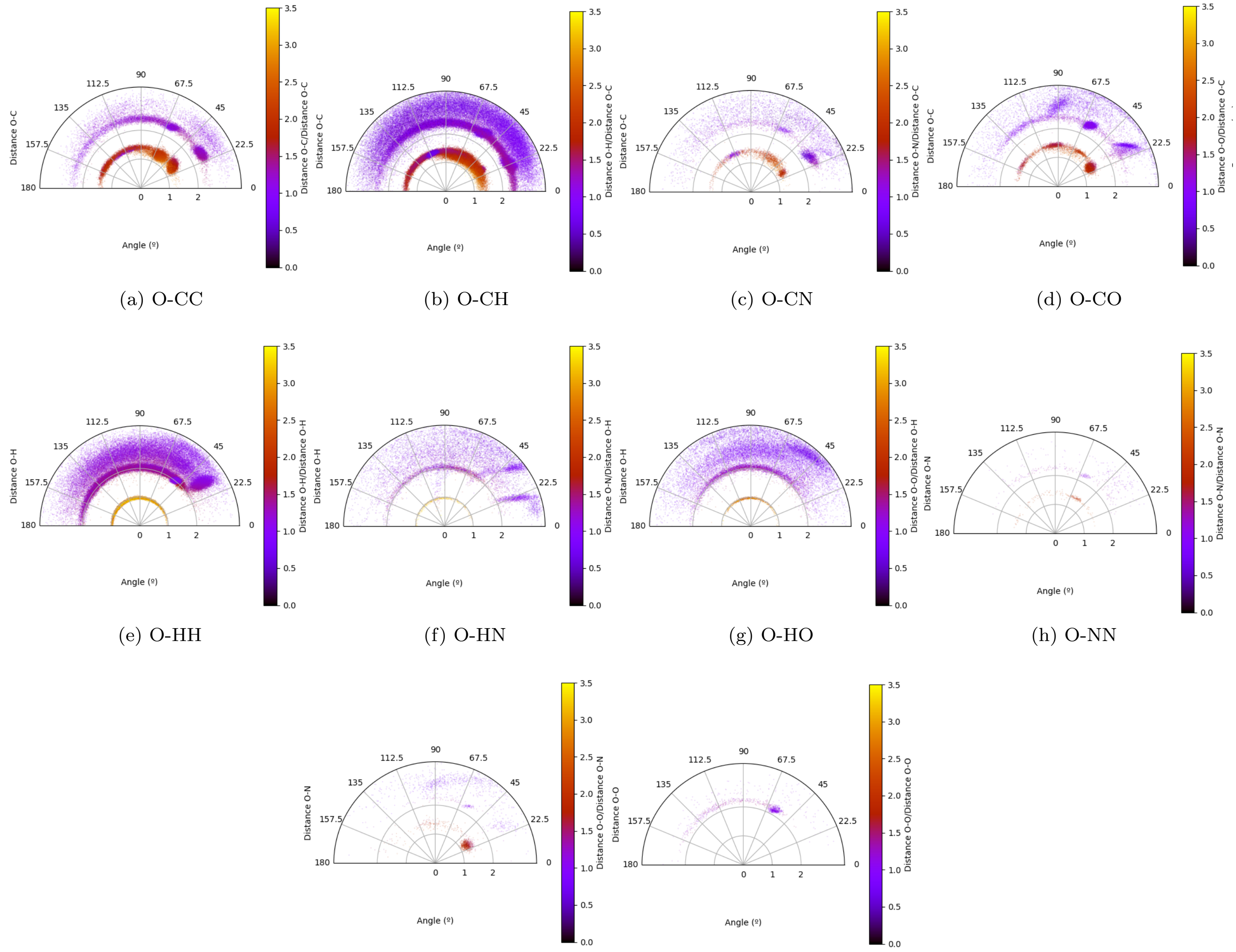

(a) O-CC (b) O-CH (c) O-CN (d) O-CO

(e) O-HH (f) O-HN (g) O-HO (h) O-NN

(i) O-NO (j) O-OO

Figure 64: Optimized angular ACSF of the O atoms in the CHON subset, using a cutoff radius of 3.5 Å.

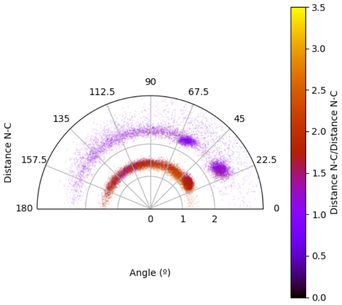

(a) N-CC

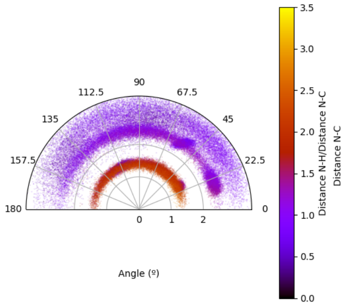

(b) N-CH

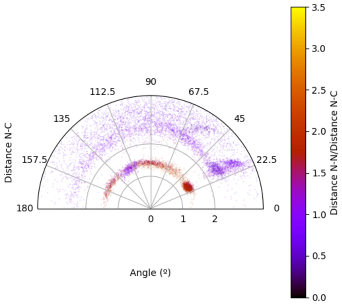

(c) N-CN

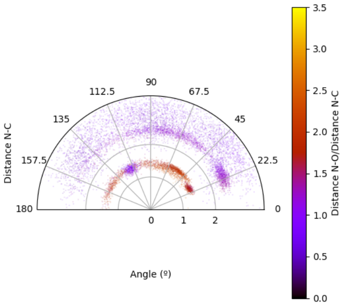

(d) N-CO

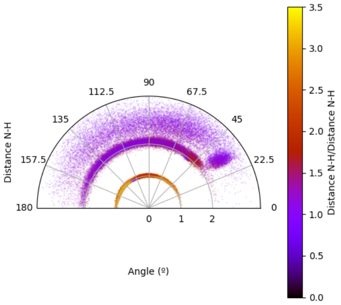

(e) N-HH

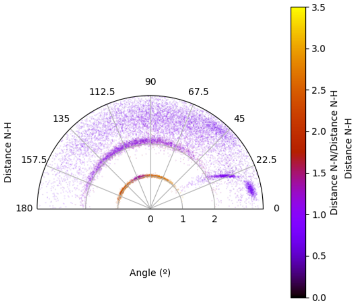

(f) N-HN

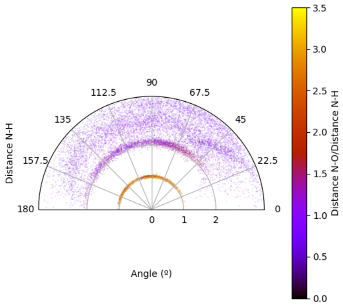

(g) N-HO

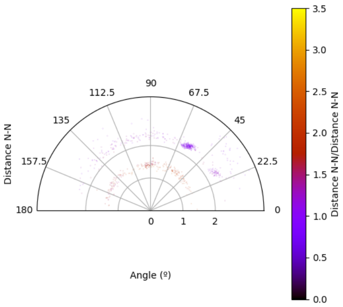

(h) N-NN

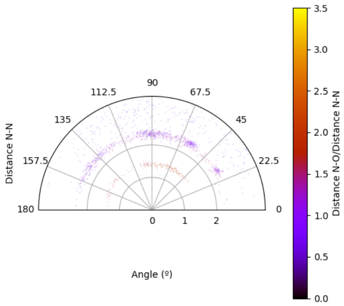

(i) N-NO

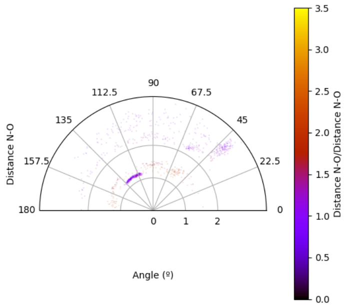

(j) N-OO

Figure 65: Optimized angular ACSF of the N atoms in the CHON subset, using a cutoff radius of 3.5 Å.

Angular ACSF with a 10.0 Å cutoff

The following figures show the self-tuned angular ACSF features within the CHON chemical space using a cutoff radius of 10.0 Å.

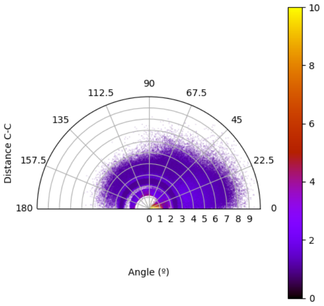

(a) C-CC

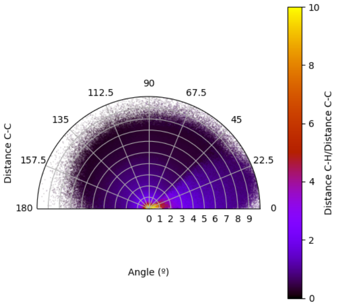

(b) C-CH

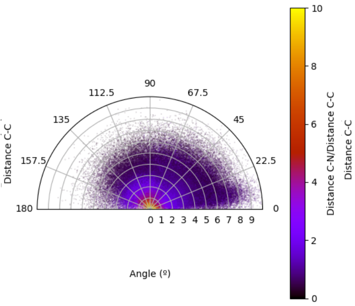

(c) C-CN

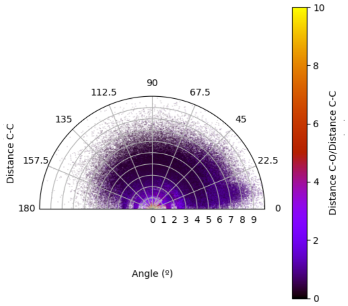

(d) C-CO

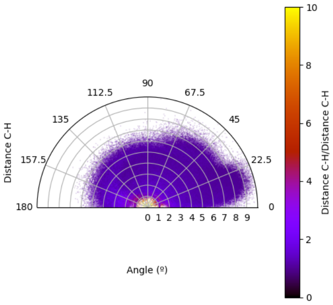

(e) C-HH

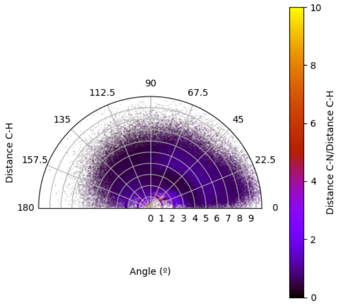

(f) C-HN

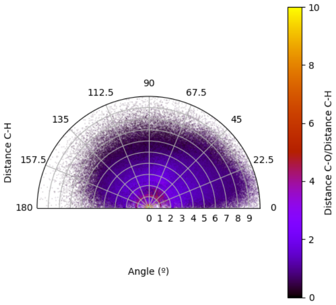

(g) C-HO

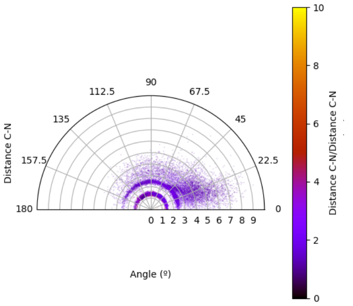

(h) C-NN

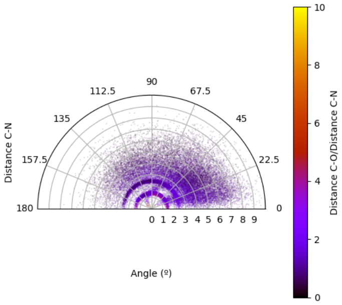

(i) C-NO

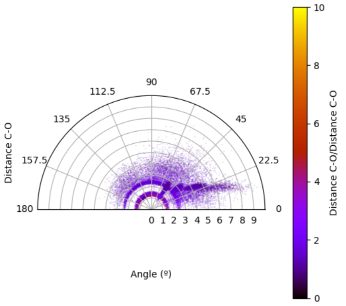

(j) C-OO

Figure 66: Optimized angular ACSF of the C atoms in the CHON subset, using a cutoff radius of 10.0 Å.

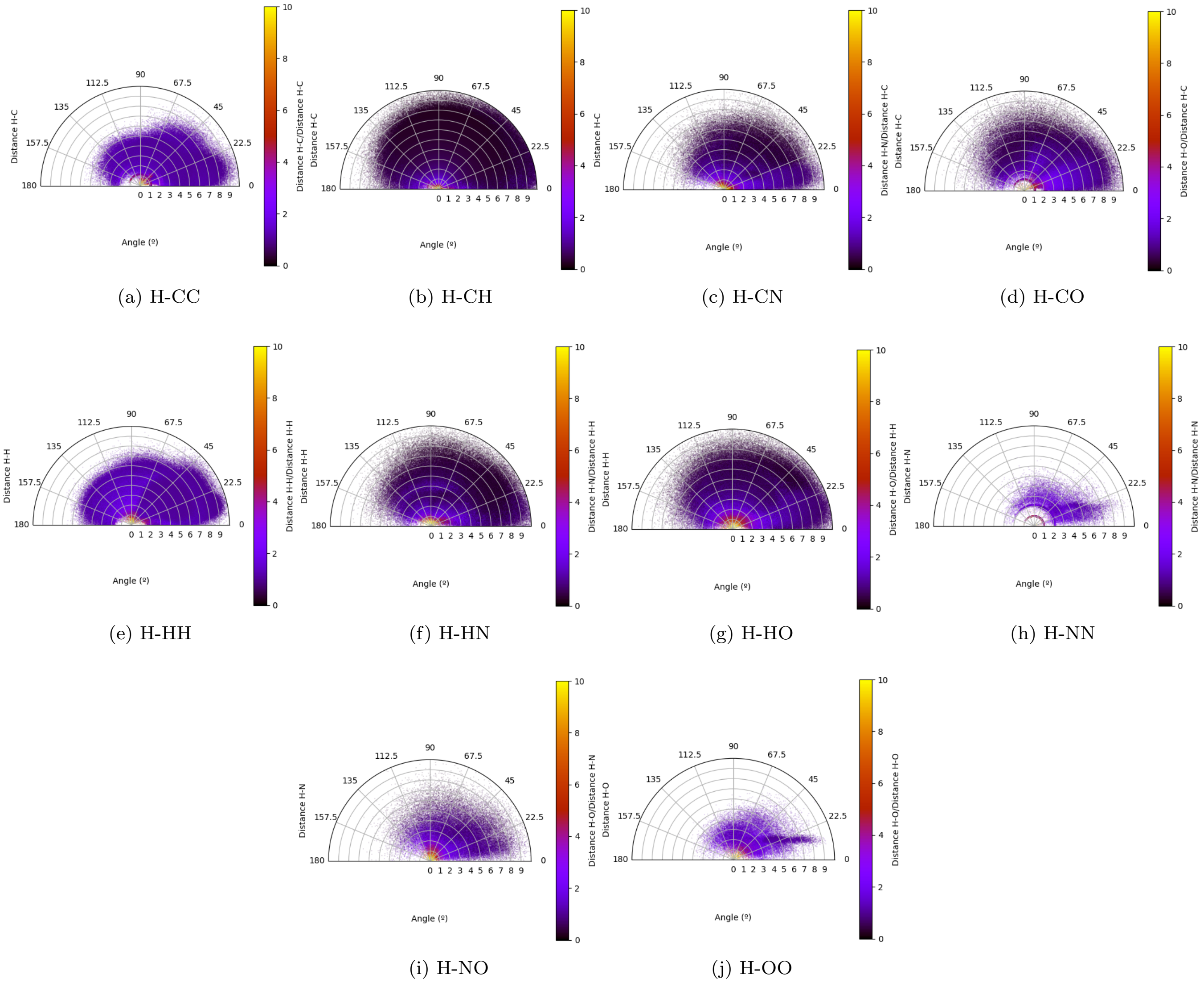

(a) H-CC (b) H-CH (c) H-CN (d) H-CO (e) H-HH (f) H-HN (g) H-HO (h) H-NN (i) H-NO (j) H-OO

Figure 67: Optimized angular ACSF of the H atoms in the CHON subset, using a cutoff radius of 10.0 Å.

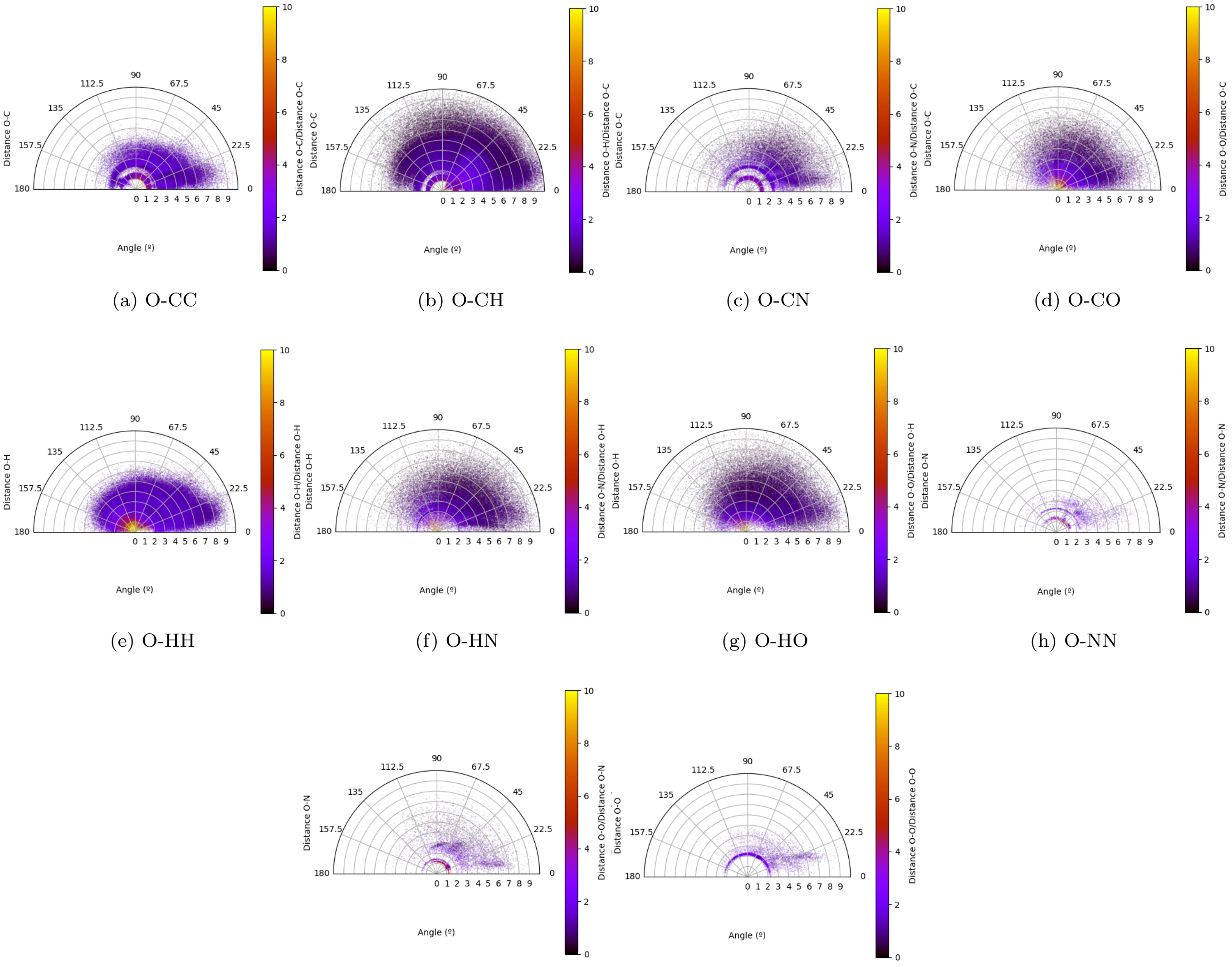

(a) O-CC

(b) O-CH

(c) O-CN

(d) O-CO

(e) O-HH

(f) O-HN

(g) O-HO

(h) O-NN

(i) O-NO

(j) O-OO

Figure 68: Optimized angular ACSF of the O atoms in the CHON subset, using a cutoff radius of 10.0 Å.

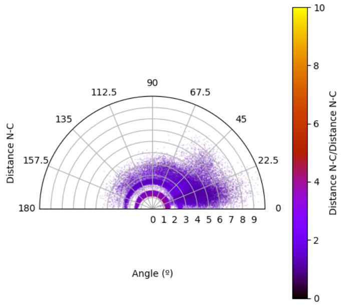

(a) N-CC

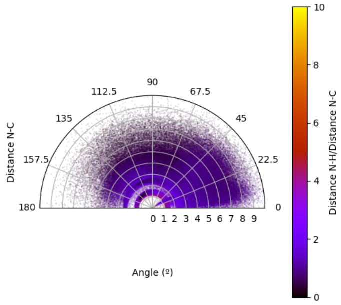

(b) N-CH

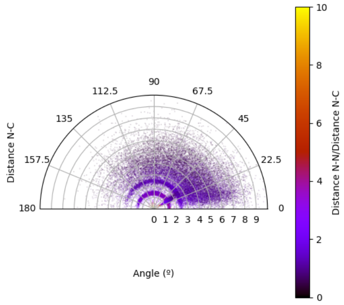

(c) N-CN

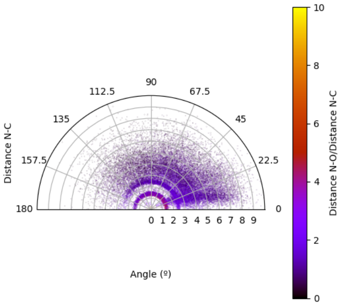

(d) N-CO

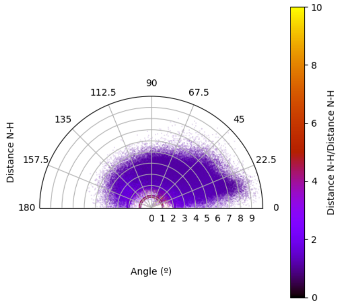

(e) N-HH

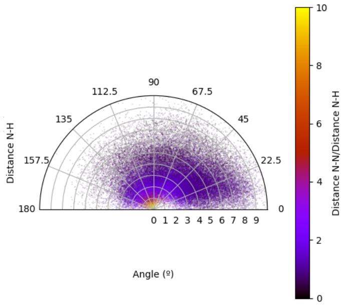

(f) N-HN

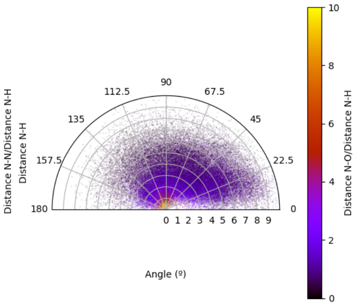

(g) N-HO

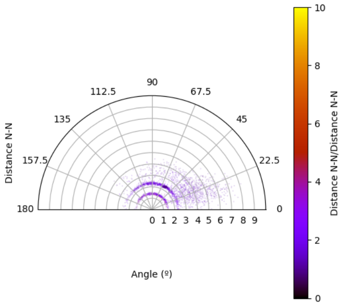

(h) N-NN

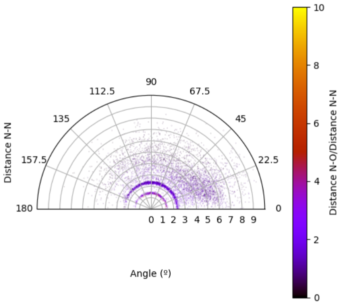

(i) N-NO

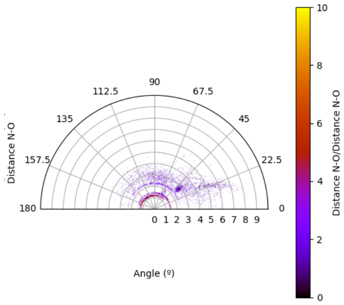

(j) N-OO

Figure 69: Optimized angular ACSF of the N atoms in the CHON subset, using a cutoff radius of 10.0 Å.

We now finally check the quality of the resultant ACSF features by training FFNN models to predict the atomic charges of the whole NNAIMQ database.[15] As such, FFNN models were trained using the previously optimized radial and angular ACSF functions. For the sake of convenience, and in order to achieve a fair comparison with the previously reported results,[15] all models were trained in an element-wise way. As for the architecture, 3 layers, interfaced through hyperbolic tangent activation functions were employed, while a linear scaling was used for the very last output layer. A total of 20 neurons was used in each of the hidden layers to match the configuration used in the original work.[15] The remaining parameters were left unmodified with respect to the indications given in Section. 3. For each element, the database was randomly split in 70 %, 20 % and 10 % subsets for training, validation and testing, respectively. As for the features, different combinations of the optimized here radial and angular ACSF functions, specified in each case, were used to train the models.

Radial (7.0 Å) + Angular (3.5 Å)

The following table comprises the error metrics made by the FFNN models trained to predict the QTAIM atomic charges of the whole NNAIMQ database,[15] using a combination of self-tuned radial and angular ACSFs with a cutoff radius of 7.0 Å and 3.5 Å , respectively.

Table 21: Performance, reported in terms of the MAE and RMSE error metrics in electrons, of the FFNN models trained to predict the QTAIM atomic charges of the NNAIMQ database.[15] All models were trained with the self-optimized radial and angular ACSF features using a cutoff radius of 7.0 and 3.5 Å, respectively.

Element	MAE			RMSE		
	Train	Test	Val	Train	Test	Val
C	0.012	0.012	0.012	0.020	0.020	0.020
H	0.007	0.008	0.008	0.010	0.011	0.011
O	0.009	0.010	0.010	0.013	0.014	0.014
N	0.020	0.024	0.024	0.027	0.033	0.032

Radial (10.0 Å) + Angular (3.5 Å)

The following table comprises the error metrics made by the FFNN models trained to predict the QTAIM atomic charges of the whole NNAIMQ database,[15] using a combination of self-tuned radial and angular ACSFs with a cutoff radius of 10.0 Å and 3.5 Å , respectively.

Table 22: Performance, reported in terms of the MAE and RMSE error metrics in electrons, of the FFNN models trained to predict the QTAIM atomic charges of the NNAIMQ database.[15] All models were trained with the self-optimized radial and angular ACSF features using a cutoff radius of 10.0 and 3.5 Å, respectively.

Element	MAE			RMSE		
	Train	Test	Val	Train	Test	Val
C	0.012	0.012	0.012	0.019	0.019	0.019
H	0.008	0.008	0.008	0.011	0.011	0.011
O	0.010	0.011	0.011	0.014	0.015	0.015
N	0.020	0.023	0.024	0.026	0.032	0.032

Radial (7.0 Å) + Angular (10.0 Å)

The following table comprises the error metrics made by the FFNN models trained to predict the QTAIM atomic charges of the whole NNAIMQ database,[15] using a combination of self-tuned radial and angular ACSFs with a cutoff radius of 7.0 Å and 10.0 Å , respectively.

Table 23: Performance, reported in terms of the MAE and RMSE error metrics in electrons, of the FFNN models trained to predict the QTAIM atomic charges of the NNAIMQ database.[15] All models were trained with the self-optimized radial and angular ACSF features using a cutoff radius of 7.0 and 10.0 Å, respectively.

Element	MAE			RMSE		
	Train	Test	Val	Train	Test	Val
C	0.014	0.014	0.014	0.021	0.022	0.022
H	0.009	0.009	0.009	0.012	0.012	0.012
O	0.011	0.012	0.012	0.015	0.017	0.016
N	0.023	0.027	0.027	0.030	0.035	0.035

Radial (10.0 Å) + Angular (10.0 Å)

The following table comprises the error metrics made by the FFNN models trained to predict the QTAIM atomic charges of the whole NNAIMQ database,[15] using a combination of self-tuned radial and angular ACSFs with a cutoff radius of 10.0 Å and 10.0 Å , respectively.

Table 24: Performance, reported in terms of the MAE and RMSE error metrics in electrons, of the FFNN models trained to predict the QTAIM atomic charges of the NNAIMQ database.[15] All models were trained with the self-optimized radial and angular ACSF features using a cutoff radius of 10.0 and 10.0 Å, respectively.

Element	MAE			RMSE		
	Train	Test	Val	Train	Test	Val
C	0.014	0.014	0.014	0.021	0.022	0.022
H	0.008	0.008	0.008	0.012	0.012	0.012
O	0.011	0.012	0.012	0.015	0.016	0.016
N	0.023	0.026	0.027	0.030	0.035	0.035

Fine-tuned hand-crafted features

The following table comprises the error metrics made by the FFNN models trained to predict the QTAIM atomic charges of the whole NNAIMQ database,[15] using the fine-tuned selection of hand-crafted ACSF features. The latter were gathered from the original NNAIMQ work.[15]

Table 25: Performance, reported in terms of the MAE and RMSE error metrics in electrons, of the FFNN models trained to predict the QTAIM atomic charges of the NNAIMQ database,[15] using the fine-tuned hand crafted features. The data was gathered from the literature.[15]

Element	MAE		RMSE	
	Train	Test	Train	Test
C	0.010	0.010	0.015	0.015
H	0.007	0.007	0.009	0.009
O	0.008	0.009	0.011	0.011
N	0.015	0.016	0.020	0.022

Finally, the following figure gathers the evolution of the average training MAEs made in the prediction of the QTAIM atomic charges of the NNAIMQ database with FFNN models trained using different features.

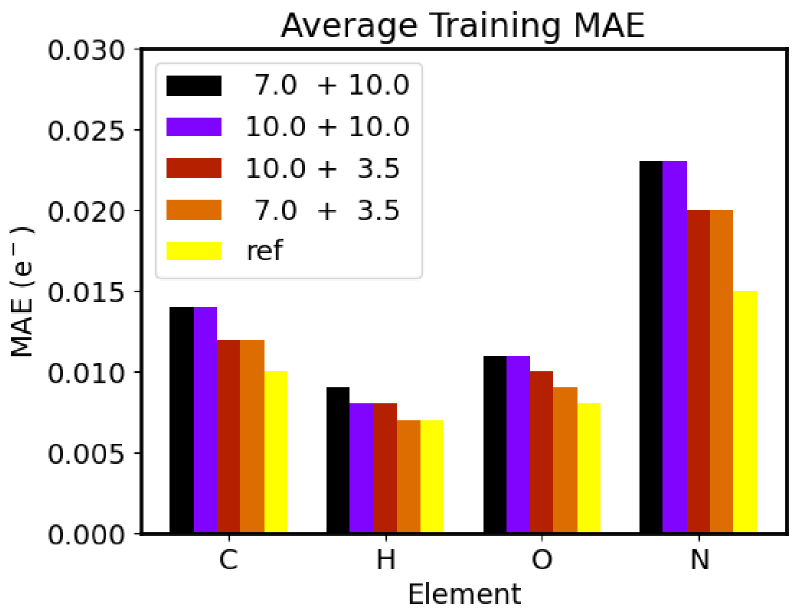

Figure 70: Average training MAE, in electrons, of the FFNN models trained to predict the QTAIM atomic charges of the whole NNAIMQ database using different features. The label *ref* is used to refer to the reference values obtained from the literature[15] and corresponding to a fine-tune manual selection of the features. On the other hand, for the remaining self-tuned features the labels show the radial and angular cutoff radii employed for the automatic selection.

As can be seen from the previous tables and figure, our self-tuned features are able to achieve, in the general case, similar performances to that afforded by the hand-crafted selection of ACSF functions. This result is quite remarkable on its own as it proves that our unsupervised ML technique effortlessly selects a collection of chemical features of comparable quality to handcrafted ones. Otherwise, the latter would require a tedious and non-trivial manual selection, which can become a major bottleneck in the development of accurate chemical ML models. It is also worth stressing that, for the sake of simplicity, the exploration of the CHON chemical space has been confined to a small fraction of the overall NNAIMQ database. As such, the resulting features are likely to be under-optimized for the actual chemical diversity encountered by the FFNN models which are instead trained on the whole database. Furthermore, we anticipate that decomposing the chemical space arising from different molecules will necessitate a significantly larger number of structures compared to exploring the conformational space of a single molecule. As a result, these features may potentially underestimate the actual performance of our approach, which could be further improved by means of a more extensive sampling of the space being, however, beyond the scope of this work.

Actually these trends are directly reflected in Fig. 70 which shows how the prediction accuracy of N atoms is far from that achieved by the reference results. This findings arises form the lower abundance of N atoms in the employed here database which reduces both the number of training data as well as the number of N environments seen by the GMM models. As such, the resultant N ACSF features are of considerably lower quality than those found for the remaining elements. Finally, it is also worth mentioning that the combination of cutoff radii of 7.0 and 3.5 Å for the radial and angular domains affords the best results, in agreement with the trends found throughout the main manuscript.

All things considered, these results underscore the appropriateness of our approach in distilling accurate and valuable local chemical descriptors for diverse molecular systems. The resulting features exhibit remarkable quality, demonstrating comparable performance to manually fine-tuned descriptor parameters, even when provided with only a small portion of the entire database. Consequently, this strategy represents a significant advancement in the automation of chemical feature creation, mitigating the costs and biases inherent in the tedious manual (conventional) approach.

References

[1] M. J. Frisch, G. W. Trucks, H. B. Schlegel, G. E. Scuseria, M. A. Robb, J. R. Cheeseman, G. Scalmani, V. Barone, B. Mennucci, G. A. Petersson, H. Nakatsuji, M. Caricato, X. Li, H. P. Hratchian, A. F. Izmaylov, J. Bloino, G. Zheng, J. L. Sonnenberg, M. Hada, M. Ehara, K. Toyota, R. Fukuda, J. Hasegawa, M. Ishida, T. Nakajima, Y. Honda, O. Kitao, H. Nakai, T. Vreven, J. A. Montgomery, Jr., J. E. Peralta, F. Ogliaro, M. Bearpark, J. J. Heyd, E. Brothers, K. N. Kudin, V. N. Staroverov, R. Kobayashi, J. Normand, K. Raghavachari, A. Rendell, J. C. Burant, S. S. Iyengar, J. Tomasi, M. Cossi, N. Rega, J. M. Millam, M. Klene, J. E. Knox, J. B. Cross, V. Bakken, C. Adamo, J. Jaramillo, R. Gomperts, R. E. Stratmann, O. Yazyev, A. J. Austin, R. Cammi, C. Pomelli, J. W. Ochterski, R. L. Martin, K. Morokuma, V. G. Zakrzewski, G. A. Voth, P. Salvador, J. J. Dannenberg, S. Dapprich, A. D. Daniels, O. Farkas, J. B. Foresman, J. V. Ortiz, J. Cioslowski and D. J. Fox, *Gaussian 09 Revision E.01*, Gaussian Inc. Wallingford CT 2009.

[2] T. A. Keith, *AIMALL; TK Gristmill Software: Overland Park KS, USA, 2019.*

[3] A. Martín Pendás and E. Francisco, *Promolden. A QTAIM/IQA code (Avaliable from the authors upon request).*

[4] W. Kabsch, *Acta Crystallogr., Sect. A.*, 1976, **32**, 922–923.

[5] J. D. Hunter, *Comput. Sci. Eng.*, 2007, **9**, 90–95.

[6] F. Chollet, *Keras*, https://keras.io, 2015.

[7] M. Abadi, A. Agarwal, P. Barham, E. Brevdo, Z. Chen, C. Citro, G. S. Corrado, A. Davis, J. Dean, M. Devin, S. Ghemawat, I. Goodfellow, A. Harp, G. Irving, M. Isard, Y. Jia, R. Jozefowicz, L. Kaiser, M. Kudlur, J. Levenberg, D. Mané, R. Monga, S. Moore, D. Murray, C. Olah, M. Schuster, J. Shlens, B. Steiner, I. Sutskever, K. Talwar, P. Tucker, V. Vanhoucke, V. Vasudevan, F. Viégas, O. Vinyals, P. Warden, M. Wattenberg, M. Wicke, Y. Yu and X. Zheng, *TensorFlow: Large-Scale Machine Learning on Heterogeneous Systems*, 2015, https://www.tensorflow.org/, Software available from tensorflow.org.

[8] J. S. Smith, O. Isayev and A. E. Roitberg, *Chem. Sci.*, 2017, **8**, 3192–3203.

[9] P. Virtanen, R. Gommers, T. E. Oliphant, M. Haberland, T. Reddy, D. Cournapeau, E. Burovski, P. Peterson, W. Weckesser, J. Bright, S. J. van der Walt, M. Brett, J. Wilson, K. J. Millman, N. Mayorov, A. R. J. Nelson, E. Jones, R. Kern, E. Larson, C. J. Carey, İ. Polat, Y. Feng, E. W. Moore, J. VanderPlas, D. Laxalde, J. Perktold, R. Cimrman, I. Henriksen, E. A. Quintero, C. R. Harris, A. M. Archibald, A. H. Ribeiro, F. Pedregosa, P. van Mulbregt and SciPy 1.0 Contributors, *Nat. Methods*, 2020, **17**, 261–272.

[10] P. Jylänki, J. Vanhatalo and A. Vehtari, *J. Mach. Learn. Res.*, 2011, **12**, 3227–3257.

[11] B. K. Isamura and P. L. Popelier, *AIP Adv.*, 2023, **13**, 095202.

[12] B. K. Isamura and P. L. Popelier, *Artif. Intell. Chem.*, 2023, **1**, 100021.

[13] S. Mirjalili, S. M. Mirjalili and A. Lewis, *Adv. Eng. Softw.*, 2014, **69**, 46–61.

[14] M. J. Burn and P. L. A. Popelier, *J. Chem. Phys.*, 2020, **153**, 054111.

[15] M. Gallegos, J. M. Guevara-Vela and Á. Martín Pendás, *J. Chem. Phys.*, 2022, **156**, 014112.
